# scRNA-seq assessment of the human lung, spleen, and esophagus tissue stability after cold preservation

**DOI:** 10.1186/s13059-019-1906-x

**Published:** 2019-12-31

**Authors:** E. Madissoon, A. Wilbrey-Clark, R. J. Miragaia, K. Saeb-Parsy, K. T. Mahbubani, N. Georgakopoulos, P. Harding, K. Polanski, N. Huang, K. Nowicki-Osuch, R. C. Fitzgerald, K. W. Loudon, J. R. Ferdinand, M. R. Clatworthy, A. Tsingene, S. van Dongen, M. Dabrowska, M. Patel, M. J. T. Stubbington, S. A. Teichmann, O. Stegle, K. B. Meyer

**Affiliations:** 1 Wellcome Sanger Institute, Wellcome Genome Campus, Hinxton, Cambridgeshire, CB10 1SA UK; 2 European Molecular Biology Laboratory - European Bioinformatics Institute (EMBL-EBI), Wellcome Genome Campus, Hinxton, Cambridgeshire, CB10 1SD UK; 3 Department of Surgery, University of Cambridge and NIHR Cambridge Biomedical Research Centre, Cambridge, CB2 0QQ UK; 4 MRC Cancer Unit, Hutchison-MRC Research Centre, University of Cambridge, Cambridge, CB2 0XZ UK; 5 Molecular Immunology Unit, Department of Medicine, Cambridge, CB2 0QQ UK; 6 10x Genomics Inc., 6230 Stoneridge Mall Road, Pleasanton, CA 94588 USA

**Keywords:** Single-cell RNA sequencing, Human, Spleen, Esophagus, Lung, Ischemic time

## Abstract

**Background:**

The Human Cell Atlas is a large international collaborative effort to map all cell types of the human body. Single-cell RNA sequencing can generate high-quality data for the delivery of such an atlas. However, delays between fresh sample collection and processing may lead to poor data and difficulties in experimental design.

**Results:**

This study assesses the effect of cold storage on fresh healthy spleen, esophagus, and lung from ≥ 5 donors over 72 h. We collect 240,000 high-quality single-cell transcriptomes with detailed cell type annotations and whole genome sequences of donors, enabling future eQTL studies. Our data provide a valuable resource for the study of these 3 organs and will allow cross-organ comparison of cell types.

We see little effect of cold ischemic time on cell yield, total number of reads per cell, and other quality control metrics in any of the tissues within the first 24 h. However, we observe a decrease in the proportions of lung T cells at 72 h, higher percentage of mitochondrial reads, and increased contamination by background ambient RNA reads in the 72-h samples in the spleen, which is cell type specific.

**Conclusions:**

In conclusion, we present robust protocols for tissue preservation for up to 24 h prior to scRNA-seq analysis. This greatly facilitates the logistics of sample collection for Human Cell Atlas or clinical studies since it increases the time frames for sample processing.

## Background

High-throughput single-cell RNA sequencing (scRNA-seq) techniques have developed rapidly in recent years, making it feasible to generate transcriptional profiles from thousands of cells in parallel [1–7]. This technology has deepened our understanding of the cell types within tissues, their interactions, and cellular states [1, 4, 8–16]. It is also a cornerstone of the Human Cell Atlas Project (HCA [17–19];), a large collaborative initiative which aims to identify every cell type in the human body. Human samples present particular logistical challenges: the clinic may be distant from the processing lab and tissue can become available at short-notice and/or at inconvenient times. These scenarios necessitate a fast, simple method of preserving samples that requires minimal processing at the clinic.

To address the logistical difficulties and rapid transcriptional changes/stress responses observed upon tissue dissociation [20, 21] or storage [22], a range of cell freezing or fixation methods have been developed. Guillaumet-Adkins et al. [23] demonstrate that although viability is reduced, the transcriptional profiles from cultured cells or minced mouse tissue biopsies cryopreserved with DMSO are not significantly altered. However, some cell types are more vulnerable to freezing than others, for example, in human endometrium biopsies, stromal cells survive freezing better than epithelial cells [24]. Fixation of cells with traditional crosslinking fixatives [25], reversible crosslinkers [26], non-crosslinking alternatives such as methanol [27], and other novel stabilization reagents [28] has also been tested. Fixation halts transcriptional change and stabilizes cell types, although it usually creates 3′ bias. Thus far, these agents have been tested on dissociated cells, or at best minced tissues, rather than intact tissue pieces. Unfortunately, dissociation prior to transportation is often not practical with human clinical samples and dissociating preserved/fixed tissue pieces using traditional mechanical or enzymatic dissociation methods is often challenging.

Hypothermic preservation of intact tissues, as used during organ transplant procedures, has been optimized to reduce the effects of ischemia (lack of blood supply) and hypoxia (oxygen deficiency) during storage at 4 °C [29]. Clinically, the kidneys are transplanted with a median cold ischemic time of 13 h and maximum around 35 h; the lungs with median 6.4 h and maximum 14 h. However, the human kidney and pancreas maintain their function even after 72 h storage in the University of Wisconsin solution, and the liver for up to 30 h [30]. Wang et al. [31] demonstrated that intact mouse kidneys could be stored in HypoThermosol FRS media for up to 72 h before dissociation and scRNA-seq without altering the transcriptomic profile or cellular heterogeneity of kidney-resident immune cells. Considering human tissue research, this method has major advantages. Firstly, it requires no processing of the sample at the collection site; the clinician can immerse an intact piece of tissue in cold HypoThermosol FRS solution and store or ship this on ice to the receiving laboratory, where all other tissue processing can take place. This can be done in a standardized and reproducible way. Secondly, it utilizes a commercially available, chemically defined, non-toxic, and ready-to-use hypothermic preservation solution, designed to mimic clinical organ preservation.

One limitation of the Wang et al. study, however, was that it only studied murine kidney. To provide maximum utility for human research, scRNA-seq from multiple human organs with different ischemic sensitivities is required. Ferreira et al. [22] saw organ-related variation in the number of genes that changed expression with post-mortem interval (warm ischemia) in Genotype-Tissue Expression (GTEx) project bulk RNA-seq [32]. For example, the spleen showed relatively little change, whereas the esophagus mucosa and lung altered their transcriptional profiles more significantly; the esophagus showing a response that peaked and declined, whereas the lung had a more sustained gene expression change. GTEx data [32] also demonstrates non-random, transcript-dependent changes in post-mortem RNA degradation and apparent gene expression [33, 34].

In this study, we aimed to identify a method of tissue preservation that would stabilize intact human tissue samples for scRNA-seq but that requires minimal processing at the clinic and allows sample transportation time. In order to contribute to the Human Cell Atlas, we tested the method on three human primary tissues expected to have different sensitivities to ischemia [22]: spleen (most stable), esophagus mucosa, and lung (least stable) [22]. These tissues contain cell types ranging from immune cells to keratinocytes. Samples were obtained from deceased organ donors and rapidly perfused with cold organ preservation solution following death. Our dataset of 240,000 single cells includes the largest published datasets on human esophagus and spleen to date, which we provide in an easy to browse data portal: www.tissuestabilitycellatlas.org. We show that storing intact tissue pieces from these 3 organs at 4 °C in HypoThermosol FRS for 24 h, or in most cases 72 h, had little effect on the transcriptomic profile as determined by bulk and 10x Genomics 3′ single-cell RNA sequencing. The diversity of populations observed in scRNA-seq data was maintained over time. This protocol should be easily adopted by many clinical sites and permits at least a 24-h time window for shipping of samples to collaborators, therefore increasing accessibility to fresh human tissue for research.

## Results and discussion

### Good scRNA-seq data quality after cold storage

We obtained the lung, esophagus, and spleen samples from 12 organ donors (Additional file 2: Table S1). The transplant surgeon assessed each organ as overall healthy in appearance. Whole genome sequencing (WGS) was carried out for each individual, confirming that none of the study participants displayed gross genomic abnormalities (Additional file 1: Figure S1). Furthermore, for each donor, histology sections were produced from the 12- or 24-h time points of each tissue, stained with hematoxylin and eosin, and assessed by a pathologist (Additional file 1: Figure S2). This confirmed all tissue sections as healthy, except one donor with possible lung hypertension. Heterogeneity between tissue sections, for example, the presence of glands, and amount of inflammation in some sections (Additional file 1: Figure S2), is likely to impact profiling by scRNA-seq.

**Fig. 1:**
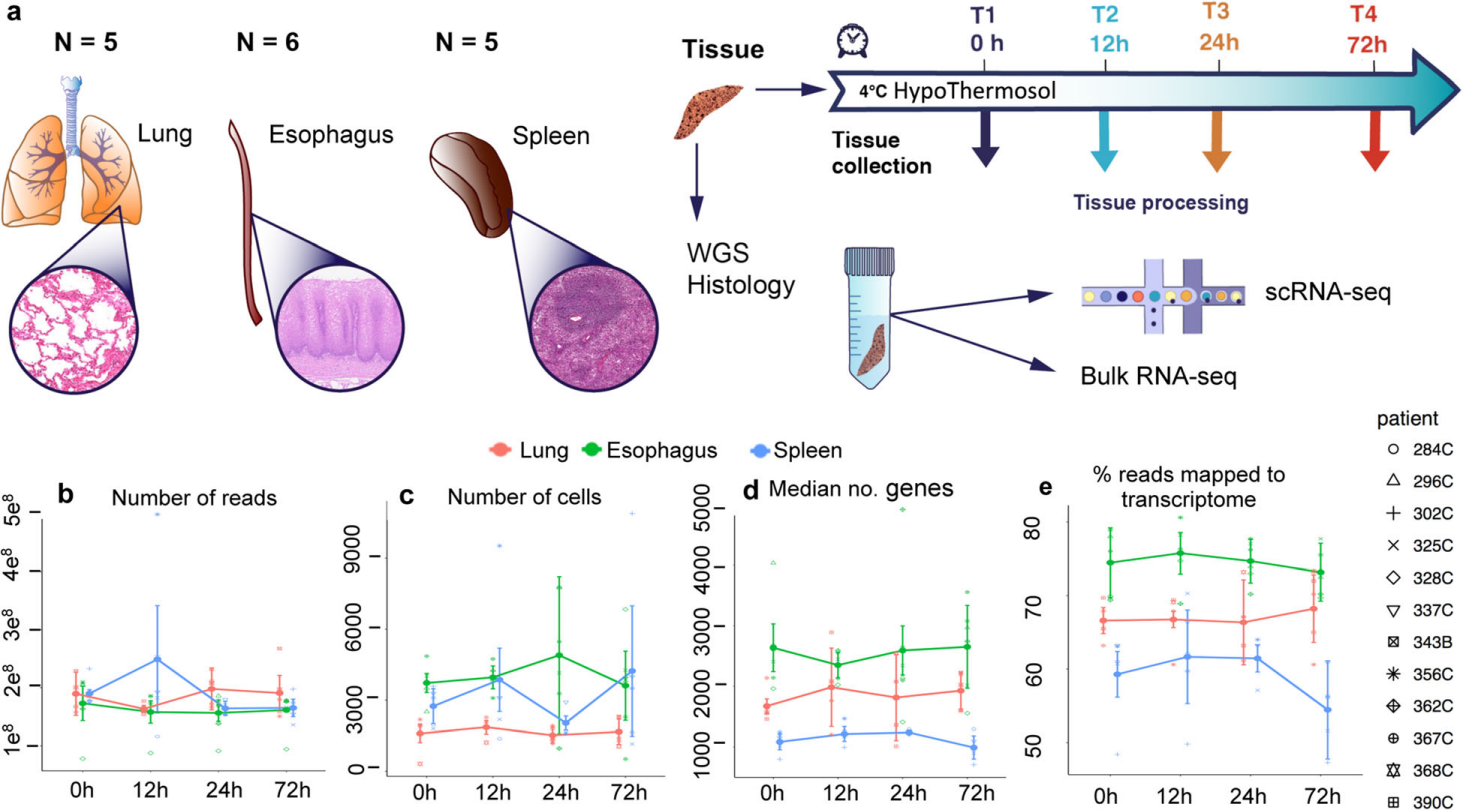
scRNA-seq quality metrics remain stable for at least 24 h of cold storage. **a** Experimental design: samples from the lung, esophagus, and spleen were collected from 5 or 6 donors each and stored as whole organ pieces at 4 °C for different time points prior to tissue processing for scRNA-seq and bulk RNA-seq. **b**–**e** Change of quality metrics of scRNA-seq data obtained with time, showing the **b** number of reads per sample, **c** number of cells per sample, **d** median number of genes detected per cell, and **e** number of genes confidently mapped to the transcriptome

Samples of lung parenchyma, esophagus mid-region, and spleen (*n* ≥ 5; experimental design, Fig. 1a) were placed into 4 °C HypoThermosol FRS solution immediately after collection (within 2 h of cessation of circulation with cold perfusion) and were kept at 4 °C until used for scRNA-seq. For the majority of lung donors (*n* = 5), tissue pieces were also flash frozen at the clinic (earliest possible time point), before transport to the processing site for bulk RNA sequencing. Following transport, fresh samples were either dissociated immediately (T0) or stored at 4 °C for 12, 24, or 72 h cold ischemic time prior to processing to single-cell suspension (Fig. 1b, Additional file 2: Table S1). The T0 time point varied depending on the length of the organ transplant procedure, time required to collect samples in the clinic, and speed of courier delivery (on average 4 h of cold ischemia from cessation of circulation to receipt of tissue at the processing laboratory). Other time points were processed at 12 h, 24 h, and 72 h after T0. Cells were analyzed by 10X 3′ v2 scRNA-seq (Fig. 1c), and the number of cells obtained for each sample is given in Additional file 3: Table S2. At each time point, tissue pieces were also flash frozen for bulk RNA-seq analysis.

After alignment and normalization of scRNA-seq data, quality control metrics were assessed for all samples (Fig. 1, Additional file 1: Figure S3). The number of reads per sample, number of cells per sample, median number of genes per cell, and other quality metrics did not change significantly over time for the lung and esophagus, but we did observe changes in the spleen at the 72-h time point (Fig. 1b–d, Additional file 1: Figure S3). The percentage of reads confidently (QC = 255) mapped to the transcriptome was stable for all samples except for the spleen at 72 h (Fig. 1.e). RNA quality was good and did not change with time in any of the tissues (Additional file 4: Table S3; RIN > 7 for the majority of bulk RNA seq samples, with the exception of four lower quality outliers in spleen mainly from a single donor). We conclude that in terms of quality metrics, we do not detect changes that are associated with the length of cold storage within 24 h of cold ischemic time.

### Reduced scRNA-seq data quality by 72 h in the spleen

**Fig. 2:**
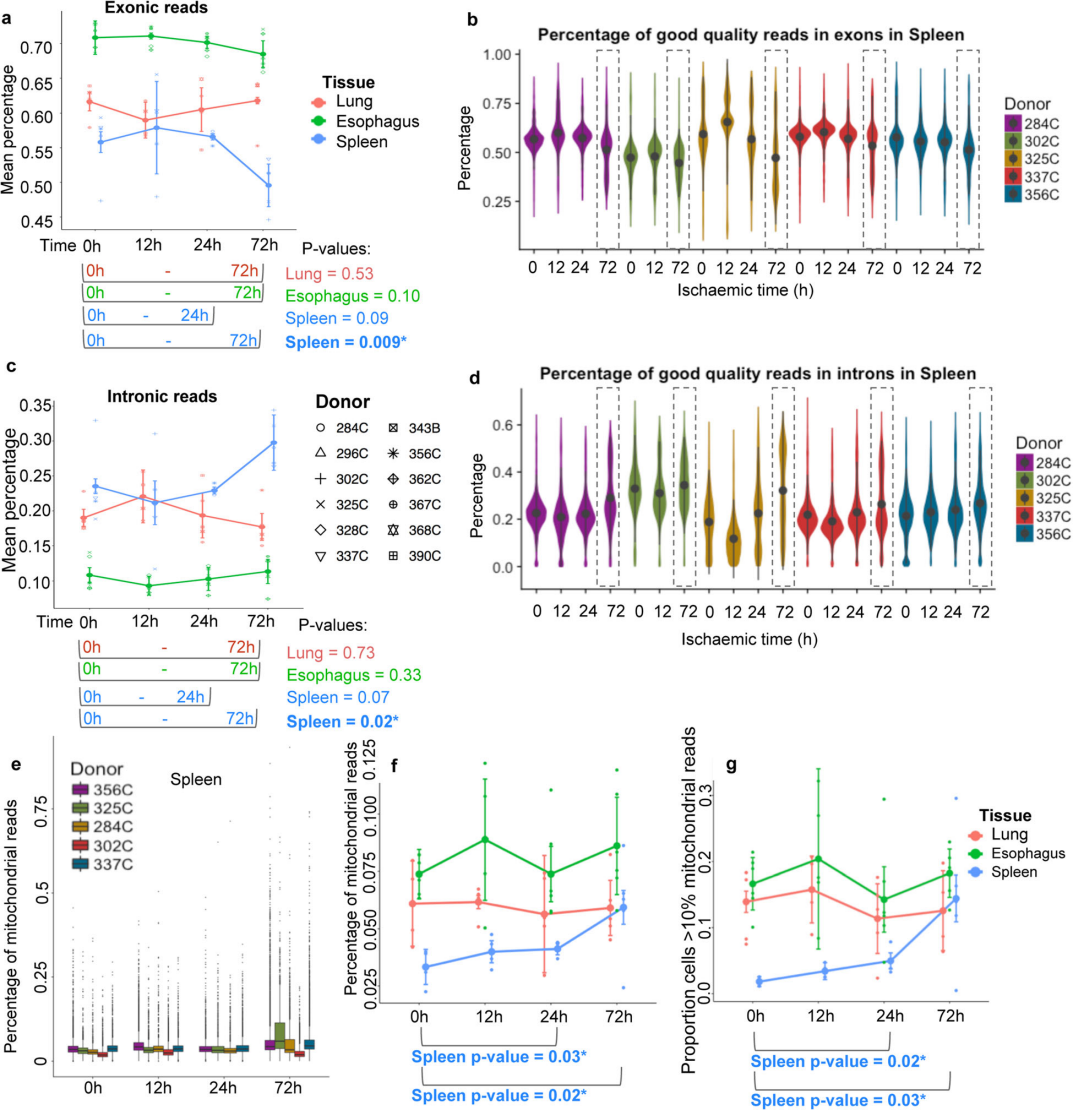
Exploration of loss of data quality with time in the spleen compared to other organs. **a** Violin plot of good quality reads mapped to exons in the spleen, **b** mean percentage of good quality exonic reads in all organs, **c** violin plot of good quality reads per exon in the spleen, **d** mean percentage of intronic reads across all organs, **e** box plot of percentage of mitochondrial reads in the spleen, **f** mean percentage of mitochondrial reads across all organs, and **e** percentage of cells with greater than 10% mitochondrial reads. The tissue of origin is indicated by color

While the majority of quality metrics did not change with time, we further studied the observed decline in confidently mapped reads in the spleen. We identified a statistically significant decrease in the percentage of reads in exons that was only observed in the spleen (Fig. 2a, b). Additionally, the percentage of reads in introns increased with storage time in the spleen, but not the lung and esophagus (Fig. 2c, d). The change in proportion of good quality reads in the spleen at 72 h (Fig. 2b, c) may lead to cell type-specific differences that are further explored later. This skewing between intronic and exonic reads becomes even more apparent when only the top and bottom quartile of cells (with respect to intronic and exonic alignment) are examined over time (Additional file 1: Figure S4). This result implies that non-spliced reads are more stable to degradation.

We next assessed the proportion of mitochondrial reads. This is a commonly used quality metric indicative of cellular stress [35], for example, induced by tissue dissociation or storage. Cells with high percentages of mitochondrial reads are generally excluded from analysis [36]. In our data, the fraction of mitochondrial reads was low, with no significant change in proportion, except in the spleen where mitochondrial reads increase by 72 h in 4 out of 5 donors (Fig. 2e, f). This is also apparent when examining the number of cells with mitochondrial percentage higher than 10%, which significantly increases with time in the spleen only (Fig. 2g).

### Effect of time on doublet rates and empty droplets

Doublet scores were calculated for each cell in every sample, and these did not change with time for any of the three tissues (Additional file 1: Figure S5).

**Fig. 3:**
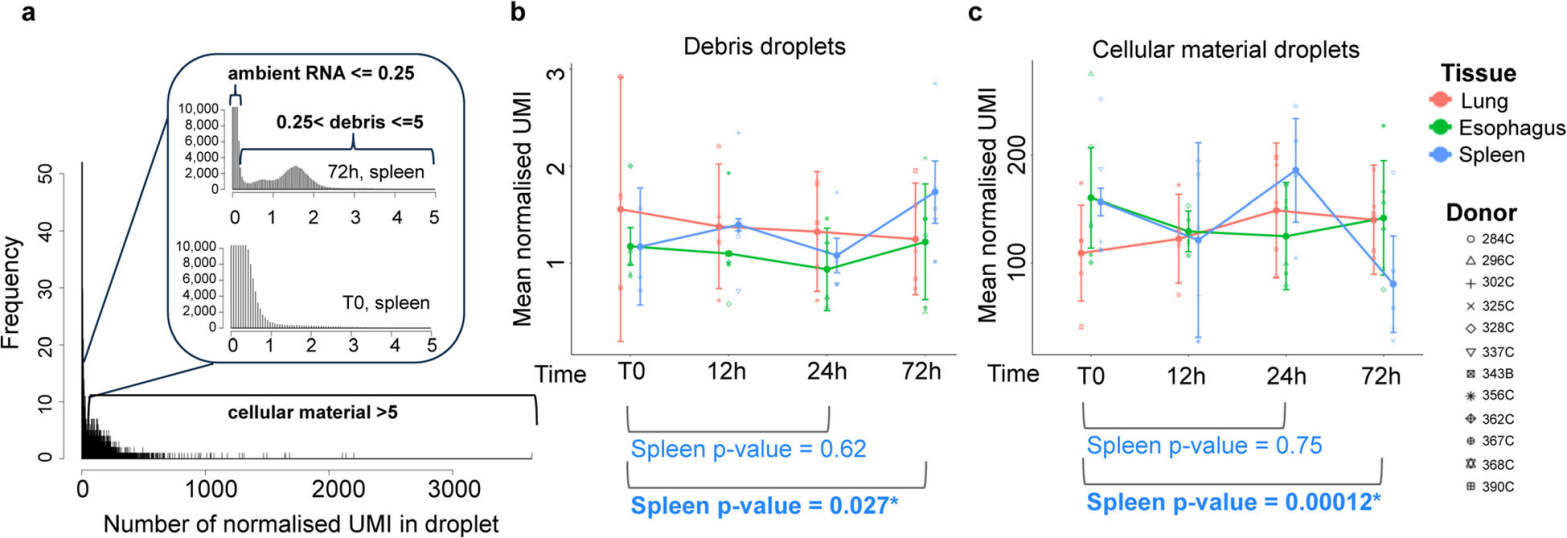
Loss of data quality is associated with increased “ambient RNA” and “debris” reads in the data. **a** Average spread of normalized UMI counts per droplet in the spleen, which were classified into ambient RNA, debris, and cellular material. **b** Mean values of normalized UMI in droplets containing debris or **c** cellular material. Individual sample means are shown for each donor with corresponding shape; color represents tissue. Means across donors per time point are shown by filled circles; whiskers represent standard deviation. *p* values were gained by Student’s paired (T0 vs 72 h) and non-paired (T0 vs 24 h) *t* test

We next evaluated changes in non-cellular droplets. All droplet sequencing reactions generate many droplets that do not contain cells but capture acellular mRNA, often referred to as “ambient RNA” or “soup” [37]. We normalized the number of UMI by read depth and set arbitrary thresholds to define “ambient RNA” as 0–0.25, “debris” as 0.25–5, and “cellular material” as > 5 normalized UMI per droplet (Fig. 3a) to reflect the distribution of reads. The proportions of droplets containing UMIs in any of these intervals were not affected by time in the spleen, lung, or esophagus (Additional file 1: Figure S6). However, the mean number of normalized UMI increased in debris and decreased in cellular droplets by 72 h (but not 24 h) in the spleen (Fig. 3b, c). This was not observed in the lung or esophagus, but we note that the mean values in debris and cellular material were very variable in all three tissues.

The increasing debris in the spleen could indicate increased cellular death by 72 h. After dissociation, we observed significant variation in cell viability between samples (Additional file 1: Figure S7) that may be of biological (donor variation) or technical origin (possibly due to samples being manually counted by multiple operators throughout the study). However, viability scores became more consistent after dead cell removal. To assess if cell viability was altered in the tissue prior to dissociation, we performed TUNEL assays on T0 and 72 h tissue sections from all three tissues to visualize apoptosing cells (Additional file 1: Figure S8). TUNEL staining intensity varied both between and within individual samples, with staining being noticeably patchy. There was a trend of higher staining at 72 h for all three tissues, but T0 staining in the spleen was higher than in the other two tissues. Overall, these findings are consistent with increased cell death at later time points and with a larger effect of cell death observed in the spleen.

Since dead cells should be removed in the washing steps and viability columns, we expect not to observe the cells at the late stages of apoptosis in our sequencing data. However, we do observe more debris in the spleen by 72 h that can indicate increased sensitivity to dissociation after prolonged storage.

### Annotation of cell types

**Fig. 4:**
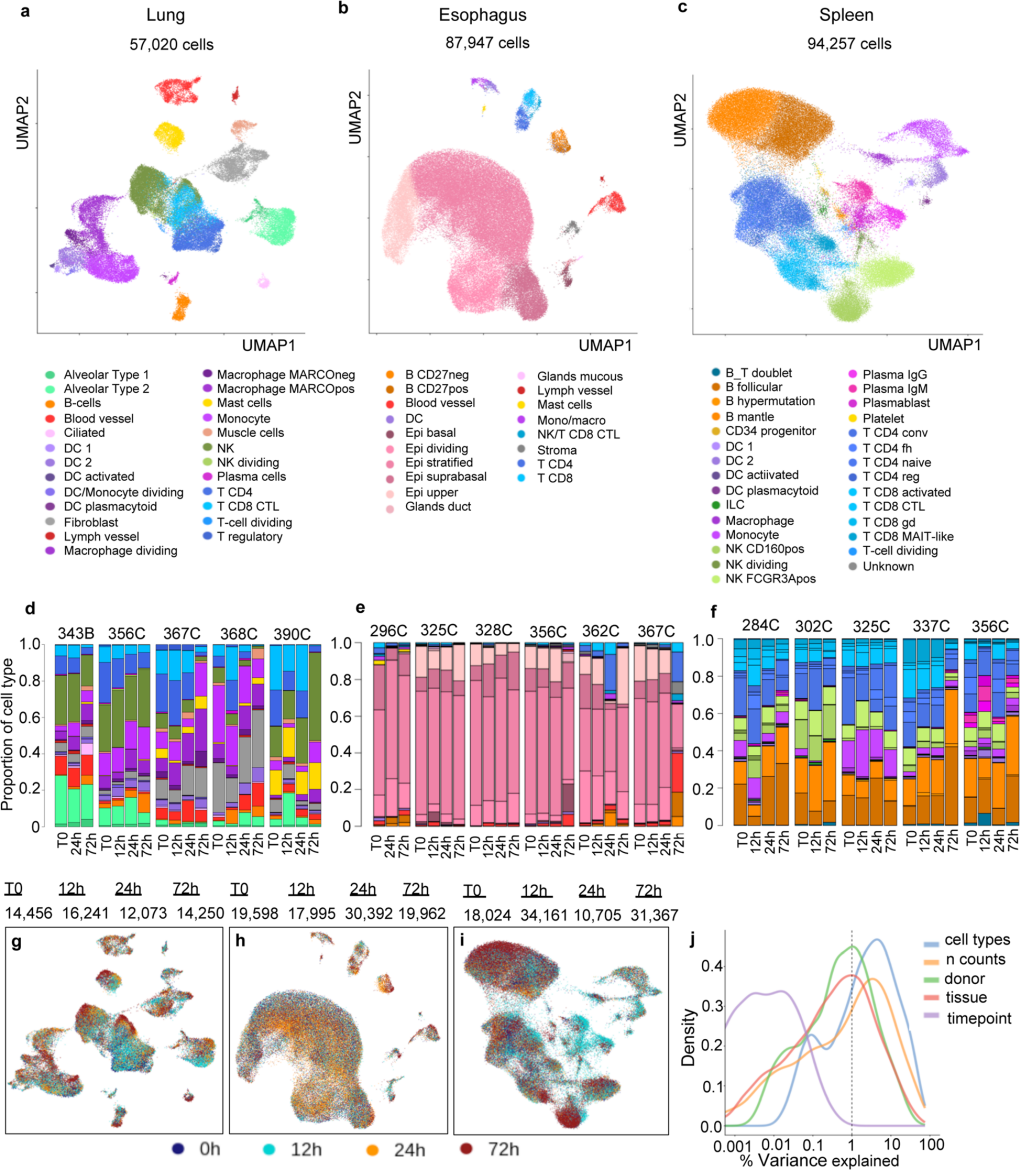
Cell types identified in different organs with time **a** UMAP projections of scRNA-seq data for the lung (*n* = 57,020), **b** esophagus (*n* = 87,947), and **c** spleen (*n* = 94,257). **d**–**f** Proportions of cells identified per donor and per time point for the **d** lung, **e** esophagus, and **f** spleen. **g**–**j** The single-cell UMAP plots for each organ with length of storage time highlighted. **j** Percent variance explained in the combined dataset by cell types, *n* counts, donor, tissue, and time points

The gene expression count matrices from Cell Ranger output were used to perform sequential clustering of cells from either whole tissues or particular subclusters. The cell type identities of the clusters were determined and annotated by observation of expression of known cell type markers (Fig. 4a–c, Additional file 1: Figure S9a-c, and Additional file 3: Table S2). Importantly, all time points and at least four different donors contributed to every cell type in all three tissues (Fig. 4d–f, Additional file 1: Figure S10, and Additional file 3: Table S2).

In the lung, 57,020 cells passed quality control and represented 25 cell types. We detected ciliated, alveolar types 1 and 2 cells, as well as fibroblast, muscle, and endothelial cells both from blood and lymph vessels. The cell types identified from the immune compartment included NK, T, and B cells, as well as two types of macrophages, monocytes, and dendritic cells (DC). Multiple DC populations such as conventional DC1, plasmacytoid DC (pcDC), and activated DC were detected and constituted 0.3% (163 cells), 0.08% (46 cells), and 0.2% (122 cells) of all cells, respectively. Lung club cell marker genes are detected in a small number of cells, but our clustering algorithm did not recognize these cells as a separate cluster (Additional file 1: Figure S11). All donors contributed to every cluster. Dividing cells formed separate clusters for T cells, DC, monocytes, NK, and macrophages.

The esophagus yielded 87,947 cells with over 90% belonging to the 4 major epithelial cell types: upper, stratified, suprabasal, and dividing cells of the suprabasal layer. The additional cells from the basal layer of the epithelia clustered more closely to the gland duct and mucous secreting cells. While all donors contributed to the basal layer, only 2 samples from a total of 23 esophagus samples provided the majority of the mucous secreting cells (0.06% from total; 55 cells; samples 325C, 12 h, and 356C, 72 h). Immune cells in the esophagus include T cells, B cells, monocytes, macrophages, DCs, and mast cells. Interestingly, almost 80% of the mast cells (87 cells) originated from a single donor (296C). Increased proportions of other immune cells (B cells, DC, monocytes/macrophages) were also noticed in this donor. This donor was the only one subjected to MACS dead cell removal, which was later excluded from the protocol due to concerns about losing larger cell types such as upper epithelial cells (0.5% of all cells in 296C, over 7% in all other donors). In addition, this donor was diagnosed with ventilator-associated pneumonia and some reports in mice indicate a link between mast cells and pneumonia infection [38, 39].

All the 94,257 cells from the spleen were annotated as immune cells. Follicular and mantle zone B cells were identified as the largest group with 17% (> 16,000 cells) and 20% (> 18,000 cells), respectively. Dividing B cells potentially undergoing affinity maturation were annotated by the expression of AICDA and detected with a frequency of 0.5% (437 cells). Over 6000 plasma cells were detected and annotated as plasmablasts, IgG, or IgM expressing plasma cells. About 90% from each of those originated from one donor 356C, which is consistent with the medical records showing chest infection in this donor. Over 28,000 T cells were annotated as CD4+ conventional, CD8+ activated, CD+4 naive, CD4+ follicular helper (fh), CD8+ MAIT-like, CD8+ gamma-delta, CD8+ cytotoxic lymphocyte (CTL), CD4+ regulatory, or dividing T cells. Two subpopulations of natural killer (NK) cells, a dividing NK population, monocytes, macrophages, and DCs were also identified. Multiple cell groups were represented in very low proportions, such as subpopulations of the DC including activated DC (0.04%), conventional DC1 (0.3%), and pcDC-s (0.3%), as well as innate lymphoid cells (0.6%), CD34+ progenitor cells (0.2%), platelets (0.08%), and an unknown population of cells positioned between T and B cell clusters (0.1%). Another group containing over 2207 cells expressing both T and B cell markers could represent the doublets of interacting cells and were called T_B doublet. Besides the plasma cell populations, multiple other cell types such as T_B doublet, conventional DC 1 and DC 2, DC plasmacytoid, and macrophages were also represented in higher proportions in donor 356C than any other donor. No stromal cells were detected, which is likely to be due to the fact that for the spleen, no enzymatic digestion was employed to release cells.

### Tissue processing signatures

We also performed bulk RNA-sequencing for each donor at each time point to assess gene expression changes over time without dissociation artifacts and to allow us to determine what gene sets are changed by dissociation, or if specific cell populations are lost. On a UMAP plot, the bulk and single-cell pseudo-bulk (sc-pseudo-bulk) samples cluster primarily by method (bulk or sc-pseudo-bulk) and by tissue of origin, but not according to the time point (Additional file 1: Figure S12). Previous work has highlighted the effect of enzymatic tissue dissociation on gene expression patterns [20]. Differential expression analysis was carried out by the Wilcoxon signed-rank test in each tissue between bulk vs sc-pseudo-bulk samples. *p* values were corrected for multiple testing via the Benjamini and Hochberg (BH) method. The genes with the highest fold changes from sc-pseudo-bulk to bulk were enriched in ribosomal genes in all three tissues (Additional file 5: Table S4). Also, a long non-coding RNA “MALAT1” appeared in the top 20 genes expressed at higher levels in sc-pseudo-bulk in all of the 3 tissues (adjusted *p* values < 0.002 in the lung, esophagus, and spleen). The high enrichment of ribosomal genes (adjusted *p* value 1.15 × 10^−6^) as well as MALAT1 (adjusted *p* value 1.15 × 10^−6^, median log2 fold change − 4.4) in sc-pseudo-bulk samples was also evident when combining all three tissues for the analysis. All of the dissociation-related FOS, FOSB, JUN, and JUNB genes [20] were significantly higher in the sc-pseudo-bulk than in the bulk samples with adjusted *p* values 1.56 × 10^−7^, 2.3 × 10^−10^, 8.7 × 10^−06^, and 6.13 × 10^−09^, respectively. Differential expression of ribosomal and early response genes was also seen in previous reports of dissociation signatures [20].

We also carried out tissue-specific analysis of differential gene expression. Genes more highly expressed in bulk derive from the cell types sensitive to dissociation. Pulmonary alveolar cells are very scarce in our single-cell lung data, but abundant in the tissue. This results in the differential expression of the marker AGER and surfactant-protein encoding genes SFTPB, SFTPA1, and SFTPA2. Other genes with high fold changes between bulk and sc-pseudo-bulk lung are blood vessel endothelial markers VWF and PECAM1. In the esophagus, stromal-specific genes FLNA and MYH11 and both KRT4 and KRT5, expressed in the majority of keratinocytes, are higher in bulk vs sc-pseudo-bulk. In the spleen, the list of top genes includes APOE, CD5L, VCAM1, HMOX1, C1QA, and C1QC, which are strongly expressed in macrophages. This suggests that our sample processing protocols are mostly affecting alveolar and blood vessel cells in the lung, stromal cells in the esophagus, and macrophages in the spleen.

### Cell type-specific changes

Having annotated cell types, it was possible to study the change in proportion of cell types over time. Cell type proportions varied greatly between samples and between donors (Fig. 4d–f, Additional file 3: Table S2, Additional file 1: Figure S13a). When examining cell type changes with time within donors, we noticed that the proportion of B cells increased in the spleen and that of T cells decreased in the lung and spleen with storage time (Additional file 3: Table S2, Additional file 1: Figure S13b, and Additional file 1: Figure S14). None of these changes were statistically significant after multiple testing corrections when comparing individual time points. However, we do observe a decrease of CD4 T cells and CD8 cytotoxic lymphocyte proportion in the lung when combining the T0, 12-h, and 24-h time points for comparison with the 72-h time point (BH-corrected *p* values < 0.01, Additional file 6: Table S5).

We next examined whether there was a cell type-specific effect of storage time on the transcriptome. Notably, UMAP plots that were calculated on highly variable genes did not reveal an obvious effect of time (Fig. 4g, h). We joined gene expression matrices for all the tissues and calculated the percentage of variability explained by different variables. Figure 4j shows that the variable donor, tissue, cell type, and number of counts account for the highest fraction of the variance explained, while the effect of storage time made the smallest contribution. This remained the case when the analysis was carried out per tissue (Additional file 1: Figure S15).

**Fig. 5:**
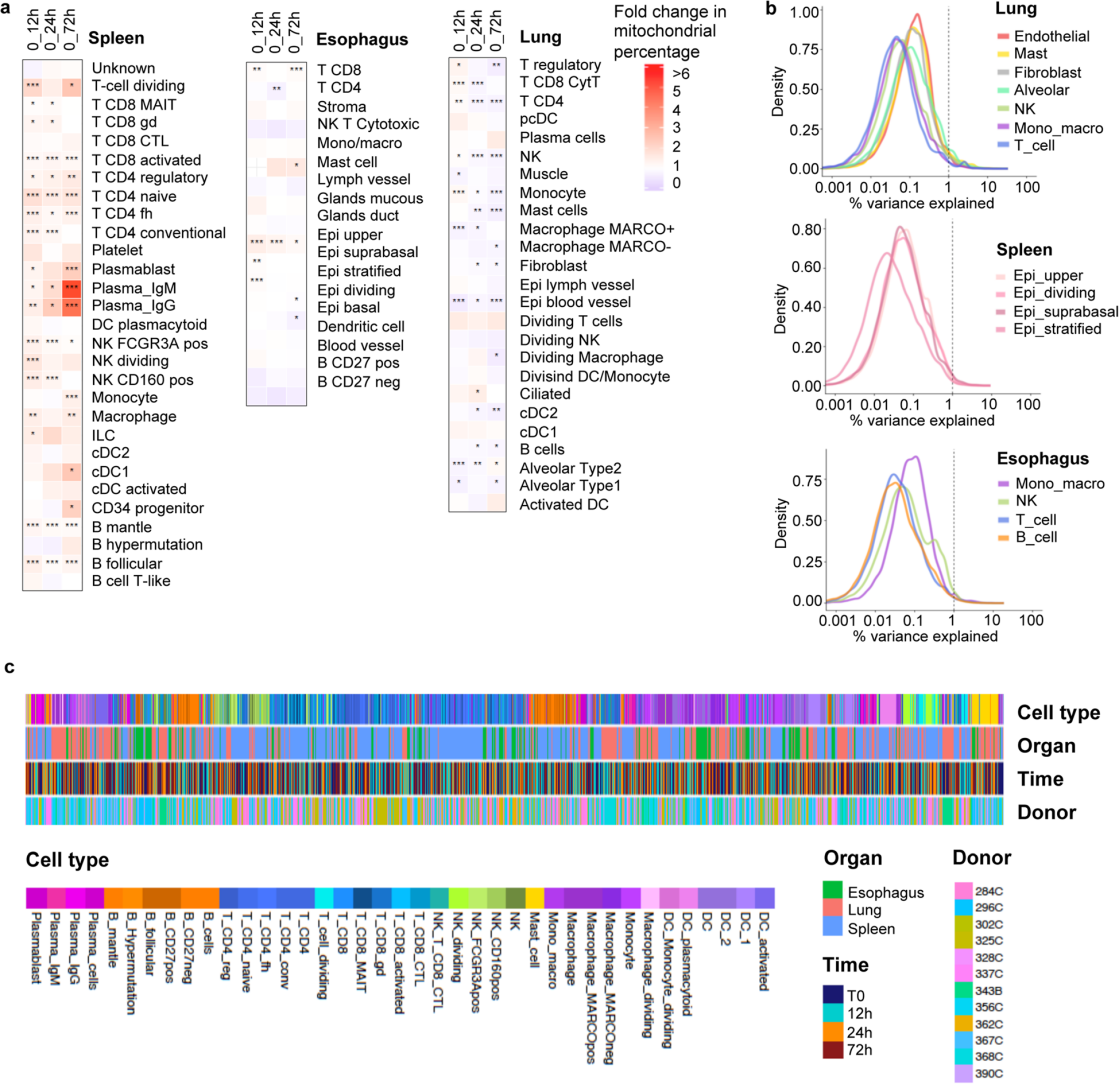
Cell type-specific changes in transcriptome. **a** Proportion of mitochondrial reads relative to T0 calculated for the spleen, esophagus, and lung. The fold change (FC) of mitochondrial percentage is measured in every cell type between T0 and 12 h, 24 h, and 72 h. FC is indicated by color with white indicating no fold change (FC = 1), blue indicating a drop in mitochondrial percentage, and red indicating an increase in mitochondrial percentage compared to T0 (FC > 1). The Benjamini and Hochberg (BH)-adjusted *p* values are indicated by asterisk as follows: *p value < 0.01, **p value < 0.00001, and ***p value < 0.00000001. All cells are used including those with high mitochondrial percentage (> 10%), annotated via scmap tool. Gray indicated time points with fewer than 5 cells. Missing values (no sample) are shown by a cross. **b** Percentage of variance in gene expression explained by time for cell type groups in the lung, esophagus, and spleen. Cell type groups in the lung are Endothelial (Blood vessel, Lymph vessel), Alveolar (Alveolar Type 1 and Type 2), Mono_macro (Monocyte, Macrophage_MARCOneg, Macrophage_MARCOpos), and T_cell (T_CD4, T_CD8_Cyt, T_regulatory). Cell type groups in the spleen are Mono_macro (Monocyte, Macrophage), NK (NK_FCGR3Apos, NK_CD160pos), T_cell (T_CD4_conv, T_CD4_fh, T_CD4_naive, T_CD4_reg, T_CD8_activated, T_CD8_CTL, T_CD8_gd, T_CD8_MAIT-like, T_cell_dividing), and B_cell (B_follicular, B_Hypermutation, B_mantle). **c** Hierarchical clustering of cell types of up to 10 cells per cell type per tissue per donor and time. Cell attributes (cell type, organ, time, and donor ID) are indicated by color

We next examined whether the observed increase in mitochondrial reads with time (spleen, 72 h, Fig. 2e–g) was due to a specific cell type. For this purpose, cells with high mitochondrial reads were assigned to a cell type via similarity. For each cell type and tissue, the mitochondrial percentages and their fold changes relative to T0 were calculated (Additional file 1: Figure S16, Fig. 5). The highest fold changes were present in the spleen at 72 h. While this effect was apparent in multiple cell types, it was particularly evident in plasma cells, where this effect was independently replicated in the two donors contributing the majority of this cell type (Additional file 1: Figure S17, Fig. 5a).

Next, similar cell types were joined together into larger clusters for more reliable analysis. The percentage of variability explained by time point in each of these cell type clusters was extremely low (Fig. 5b), especially compared with variables such as donor and number of counts (Fig. 4j), highlighting that for almost all cell types, cold storage time did not have a major effect.

We also examined which genes changed most with storage time in each cell type (see the “Methods” section). This analysis was carried on a per organ basis, as cold storage gene signatures derived from different cell types primarily clustered by organ of origin, rather than cell types. For example, storage-induced gene signatures from T cells, natural killer cells, and monocytes/macrophages grouped by organ (Additional file 1: Figure S18). Furthermore, the genes driving this similarity were among the top genes contributing to the ambient RNA contamination in the majority of samples (Additional file 1: Figure S19, Additional file 7: Table S6). For example, in the spleen, the plasma cell-specific genes such as JCHAIN, IGHA1, and IGLC3 are high in ambient RNA (Additional file 1: Figure S19) and are also overrepresented in the cold storage signature. This observation is consistent with high mitochondrial percentages (due to stress or cell death) observed in the plasma cells (Fig. 5a). In addition, we observed an overrepresentation of the cold storage signature genes (Additional file 5: Table S4) among the most strongly dissociation-related genes (adjusted *p* value < 0.01 and median log2 fold change < − 2, Additional file 7: Table S6) with Fisher’s exact test. We found a higher overlap of the dissociation-related signature than expected by chance in all three tissues (*p* values 2.2 × 10^−16^, 2.05 × 10^−14^, and 2.2 × 10^−16^ in the lung, esophagus, and spleen correspondingly). This can be explained either by cells becoming more sensitive to the dissociation with storage time, or by similar stress signatures being activated via storage time and dissociation independently. Therefore, the low levels of gene expression changes that we do observe with storage time are likely to be driven by stress-induced cell death leading to ambient RNA contamination.

Pairwise differential expression analysis in bulk RNA-sequencing between T0 and other time points did not yield significant genes in any tissue (Additional file 8: Table S7), further indicating that any changes observed are extremely small. For the lung, we were also able to freeze samples at the clinic immediately after collection and compare this sample to later time points. Again, no significantly differentially expressed genes were detected.

It may seem surprising to observe so few changes in gene expression with time, especially given that other studies such as the GTEx project do demonstrate such effects [22, 32]. However, it is important to note that post-mortem samples from warm autopsy were used for the GTEx project (albeit with < 24 h PMI). Our study was designed to mimic the process used during organ transplantation, in which tissues are removed rapidly (within 1 h of cessation of circulation) from cold-perfused donors and stored at 4 °C in hypothermic preservation media such as University of Wisconsin (timing is tissue-dependent; the heart can be stored for 4–6 h, lungs median 6.5 h, kidneys median 13 h). Indeed, for some organs, there is evidence that they remain functional for longer [29, 30]. Further, the work of Wang et al. [31], which looked at hypothermic preservation of mouse kidneys in HypoThermosol FRS, also demonstrated little change in gene expression over 72 h. Therefore, while it is certainly true that rapid gene expression changes will occur under certain storage conditions, at least for the organs tested in this study, it appears these can be limited by maintaining the samples cold in hypothermic preservation media. Altogether, this will be very useful for designing further studies with fresh biological samples (including biopsies from living donors) with regard to sample collection time in clinic, transport to the lab, and storage until processing is convenient.

### Mapping of cell types across organs

Having generated datasets for the esophagus, lung, and spleen, we examined if cell types that can be found in all three organs, such as immune cells, would cluster by organ or by cell type. Figure 5c shows the result of hierarchical clustering using the 1000 most highly variable genes in up to 10 cells per cell type, tissue, time, and donor. In this analysis of approximately 7500 cells, we see clear subclusters of mast cells, macrophages, and plasma cells with some substructure depending on the donor and the tissue of origin, suggesting that more extensive analysis will allow us to study tissue adaptation of different immune cell populations. Other cells such B cells sit in two groups, and dividing cells (NK, macrophages, T cells) also co-segregate. Importantly for this study, samples do not cluster by time.

### Variation in cell type contribution

Our protocols of single-cell dissociation are aimed at capturing the diversity of cell types present in each organ, but do not represent the proportion of each cell type in the original tissue. For example, the tissue dissociation protocol employed for the lung strongly enriches for immune cell types. Relatively high variability in the proportions of cell types was seen between samples. This was likely to be due to technical variation as well as underlying biological variation as indicated by the capture of rare structures such as glandular cells in only some esophagus samples, namely from donor 325C and 356C. Interestingly, histology on sections from donor 325C (12 h) confirmed the presence of glands (mucous secreting cells; Additional file 1: Figure S2) that were not present in the other esophagus samples sectioned. All other samples contained fewer than five mucous secreting cells (Additional file 3: Table S2). This exemplifies the difficulty in collecting cells from structures that are sparsely distributed, such as the glands in the esophagus, and suggests that some of the sample to sample variation is due to the underlying differences in the architecture of the specific tissue sections analyzed. A similar effect was seen for blood vessels (Fig. 4e, 367C, 72 h). Furthermore, the immune infiltrate seen on one of the histology sections (Additional file 1: Figure S2c, 362C, 24 h) is possibly reflected in an increase in immune cells (B, T, and monocytes/macrophages) at the single-cell level (Fig. 4e, 362C, 24 h).

Overall, we observe greater variability between donors than between samples. Additional file 6: Table S5 lists all cell types per tissue, 72 in total. Statistical tests (*t* tests) for changes with time yielded only two significant changes (discussed above). However, when we test differences between donors, we find that for 29 out of the 72 combinations the proportions of cell types were significantly different in at least 1 of the donors compared to the rest (one-sided ANOVA, BH-corrected *p* value < 0.05). This variability in cell type proportions per donor is also visualized in Additional file 1: Figure S12a. The cell type with the most significant variation between donors was mast cells in the lung. Other examples include NK cells in both the spleen and lung and dividing epithelial cells in the esophagus (Fig. 4d–f). This high variability between donors suggests that for the Human Cell Atlas, a large number of donors will have to be profiled to understand the range of “normal.”

## Conclusions

We present a method for cold storage of primary human tissue samples that requires no processing at the clinical site beyond sample collection and permits at least a 24-h window for shipping, tissue dissociation, and scRNA-seq. The lung and esophagus appeared stable for 72 h by all of the metrics tested. In the spleen, we observe changes in the proportion of intronic and exonic reads and an increase in the percentage of mitochondrial reads at 72 h. We demonstrate that it is possible to minimize the consequences of ischemia in various different cell types by storing the tissue samples immediately after collection in cold tissue preservation solution. We see no effect of time on the diversity of cell populations in scRNA-seq data or change in bulk RNA-seq within 24 h. This method is easy to adopt and will greatly facilitate primary sample collections for Human Cell Atlas studies. We highlight changes of cell type distribution due to anatomical heterogeneity of tissue samples and significant heterogeneity between donors, which will impact future HCA study design.

Further, we have generated detailed annotations on three primary human tissues: spleen, esophagus, and lung. This dataset of over 240,000 single cells presents a significant resource for further investigation of the biology of these tissues and contains the largest esophagus and spleen datasets to date. In addition, we make available WGS data from 13 healthy donors, including clinical metadata, allowing for future tissue-specific, single-cell eQTL studies.

## Methods

### Aim and study design

We aimed to identify a method of preserving intact human tissue samples for scRNA-seq. Three tissues, expected to have different sensitivities to ischemia (*n* = 5–6 per tissue), were chosen: spleen, esophagus, and lung. One sample was processed for 10x Genomics 3′ scRNA-seq (v2) immediately upon receipt (T0), and the remainder processed after 12 h, 24 h, and 72 h cold ischemic time. Additional samples were collected for bulk RNA extraction at each time point, and genomic DNA was also prepared for WGS from each donor. Of note, an additional lung donor was collected (376C) and samples sequenced but not included in the analysis, but these data are available in the Data Coordination Platform submission. Upon receipt, this sample was morphologically abnormal (blackened) and the resulting cell suspension contained cells with black granules, likely due to the donor being a heavy smoker for a prolonged period.

### Donor samples

All samples were obtained from the Cambridge Biorepository for Translational Medicine (CBTM) under appropriate ethical approval (see the “Ethics approval and consent to participate” section). These were primarily from donation after circulatory death (DCD) organ donors, in whom circulatory arrest followed withdrawal of life-sustaining treatment. Patient characteristics are listed in Additional file 2: Table S1, and representative histology is shown in Additional file 1: Figure S2. Upon cessation of circulation, donors were perfused with cold University of Wisconsin (UW) solution (within 12 min of asystole) and research samples collected at the end of the transplant procedure, within 1–2 h of cessation of circulation, constantly under cold ischemic conditions. Samples (typically 0.5–2 cm^3^) were maintained on ice in UW in the operating theater, then rapidly transferred into 5 ml of cold HypoThermosol FRS preservation solution, or approximately 10 ml for esophagus cylinders (sufficient to completely submerge the tissue), at 4 °C for shipping/storage (Sigma H4416). The size of samples varied by organ. For the lung and spleen, initial samples received were roughly 1.5 cm^3^ and were immediately dissected into four pieces (for 0 h, 12 h, 24 h, and 72 h time points) on receipt at the processing laboratory, then each placed into 5 ml cold HypoThermosol FRS. For the esophagus, cylindrical regions were received up to 2 cm in length from which only the thin (< 0.3 cm thick) length of mucosa was retained (see “Tissue dissociation” section). This was divided into four pieces, each stored in 5 ml cold HypoThermosol FRS either for immediate processing (0 h) or for storage at 4 °C. Of note, the lung and spleen are both soft/porous tissues and the stored esophagus mucosa was very thin, so we expect good penetration of HypoThermosol FRS into these tissues. We have not tested more solid organs with this method. Following division into four pieces for time points at the processing laboratory (typically 4–5 h following cessation of circulation), samples were dissociated for 10x Genomics 3′ single-cell sequencing (v2), and a portion flash frozen in isopentane for bulk RNA/DNA extraction as soon as possible (“T0” time point), or at 12, 24, and 72 h ischemic time following storage in the fridge (4 °C). For lung samples, it was also possible to collect an additional flash frozen sample at the clinic immediately after tissue excision, in order to compare bulk RNA between this “true zero” time and the “T0” time point, the latter being frozen on receipt at the tissue processing laboratory. The start of ischemia was defined as the point at which circulation ceased for donation after cardiac death (DCD) donors, unless they received normothermic regional perfusion with oxygenated blood (NRP, 2 h), in which case the end of NRP was used. For the one donation after brain stem death (DBD) donor in the study, start of ischemia was defined as the time at which life support was withdrawn. End of ischemia was defined as time of cell lysis or freezing; for 10x single-cell reactions, lysis occurs in the PCR step immediately after loading on 10x Genomics Chromium instrument. Cold ischemic times are available in the Data Coordination Platform metadata submission.

### Tissue section staining

Frozen optimal cutting temperature OCT compound embedded samples were cryosectioned and H&E stained to check for normal morphology. TUNEL (terminal deoxynucleotidyl transferase dUTP nick end labeling) assays for detecting apoptotic DNA fragmentation were performed using TACS®2 TdT-DAB In situ Apoptosis Detection Kit, catalog number: 4810-30-K. Sections were counterstained with methyl green for 5 min as nuclear counterstain.

### Tissue dissociation

All tissue dissociation protocols are available on protocols.io [40]: spleen (protocol 32rgqd6), esophagus epithelium (protocol 34fgqtn), and lung parenchyma (protocol 34kgquw).

Spleen (protocols.io 32rgqd6) samples from the top 5–7 mm of the organ were mechanically mashed through a 100-μM cell strainer with cold PBS, pelleted at 500×*g* for 5 min, and resuspended in cold 1× red blood cell lysis buffer (Life Technologies). Following dilution in cold PBS, pelleting at 500×*g* for 5 min, and resuspension in cold 0.04% BSA/PBS, cells were counted and viability was determined using a C-chip hemocytometer and trypan blue. Up to 10 million cells were used for MACS dead cell removal (Miltenyi; protocols.io qz5dx86), and the flow through (live cells) pelleted, resuspended in cold 0.04% BSA/PBS, and counted/viability determined using trypan blue and C-chip. Cells were loaded onto the 10x Genomics Chromium Controller following the single-cell 3′ v2 protocol, aiming for between 2000 and 5000 cell recoveries.

In the esophagus epithelium (protocols.io 34fgqtn), a cylindrical piece of the esophagus was received from the mid-region, and the mucosa (mainly epithelium) removed mechanically with forceps/scissors and divided into segments for time points (placed in HypoThermosol FRS in the fridge). The epithelium/mucosa was finely chopped with scalpels and incubated for 30 min in 0.25% trypsin-EDTA (GIBCO) containing 100 μg/ml DNase I (Sigma) at 37 °C with shaking. The sample was centrifuged, and digestion media replaced with fresh 0.25% trypsin-EDTA (GIBCO)/DNase I for 15 min at 37 °C with shaking (this protocol can also be used for stomach, in which the media change is necessary due to pH alterations as the tissue digests; this is less required for esophagus). Trypsin was neutralized with RPMI containing 20% FBS, and cells pelleted and passed through a 70-μM strainer before pelleting again and treating with 1× red blood cell lysis buffer (Life Technologies). Following dilution, pelleting, and resuspension in cold 0.04% BSA/PBS, cells were counted and viability determined using a C-chip hemocytometer and trypan blue. The resulting suspension contained a range of cell sizes, up to 50 μM. No dead cell removal was performed for esophagus samples due to the risk of losing larger cells in the MACS column (cell viability was > 70%), with the exception of the fresh sample from the first esophagus donor (296C). Cells were loaded onto the 10x Genomics Chromium Controller following the single-cell 3′ v2 protocol, aiming for 5000 cell recoveries.

In the lung (protocols.io 34kgquw), a 0.2–0.5-g piece of lung parenchyma (lower left lobe) was finely chopped with scalpels and incubated for 1 h in 0.1 mg/ml collagenase D (Sigma C5138) in DMEM with 100 μg/ml DNase I (Sigma) for 1 h at 37 °C with shaking. (This protocol was initially designed for isolation of immune cells from lung airway, a much tougher region of lung tissue; parenchyma can be dissociated with 30 min treatment; however, 1 h incubation was used for this study.) Digested tissue was mashed through a 100-μM cell strainer and washed with DMEM containing 10% FBS before centrifuging, washing, and resuspending the pellet in 1× red blood cell lysis buffer (Life Technologies). Following dilution, pelleting, and resuspension in cold 0.04% BSA/PBS, cells were counted and viability determined using a C-chip hemocytometer and trypan blue. MACS dead cell removal was performed (Miltenyi; protocols.io qz5dx86), and the flow through (live cells) pelleted, resuspended in 0.04% BSA/PBS, and counted using trypan blue and C-chip. Cells were loaded onto the 10x Genomics Chromium Controller following the single-cell 3′ v2 protocol, aiming for 5000 cell recoveries.

### Library preparation, bulk RNA, and WGS

cDNA libraries were prepared from single-cell suspensions following the 10x Genomics 3′ v2 protocol, and 2 samples per lane sequenced on HiSeq4000 with 26 bp read 1, 8 bp sample index, and 98 bp read 2 (aiming for 150 M reads/sample or ≥ 30,000 per cell).

For bulk RNA and DNA extraction, samples were flash frozen in isopentane at each time point (protocols.io qz7dx9n). Bulk RNA and DNA were prepared from frozen samples using the Qiagen AllPrep DNA/RNA mini kit and TissueLyser II. Spleen RNA samples required an additional on-column DNase digest.

RNA was quantified using the QuantiFluor RNA system (Promega) on a Mosquito LV liquid handling platform, Bravo WS, and BMG FluoSTAR Omega plate reader. Libraries (poly(A) pulldown) were prepared using the NEB RNA Ultra II Custom kit on an Agilent Bravo WS automation system, including PCR with the KAPA HiFi Hot Start Mix and dual-indexing. Libraries were cleaned on a Caliper Zephyr liquid handling system using Agencourt AMPure XP SPRI beads and quantified with the AccuClear™ Ultra High Sensitivity dsDNA Quantitation kit (Biotium). RNA integrity number (RIN) was determined for each sample by Agilent BioAnalyser RNA 6000 Nano kit. Libraries were pooled in equimolar amounts and quantified on an Agilent BioAnalyser before sequencing on an Illumina HiSeq4000, 75 bp paired end, aiming for 35 million reads per sample.

Genomic DNA from 13 donors was prepared for WGS. DNA was first sheared to 450 bp using a Covaris LE220 instrument, purified with AMPure XP SPRI beads (Agencourt) on an Agilent Bravo WS, and then libraries prepared with the NEB Ultra II custom kit on an Agilent Bravo WS system. PCR (6 cycles) was performed using the Kapa HiFi Hot Start Mix and IDT 96 iPCR tag barcodes, before purification using Agencourt AMPure XP SPRI beads on a Beckman BioMek NX96 liquid handling platform. Libraries were sequenced at 30× coverage on an Illumina HiSeqX.

### Computational analysis

#### Single-cell RNA-seq data analysis

Reads were mapped to GRCh38 1.2.0 Human Genome reference by Cell Ranger 2.0.2 pipeline. The EmptyDrops algorithm [41] was run on each sample. Identified cells were used to generate the Cell Ranger filtered count matrix. An outlier sample HCATisStabAug177276393 (spleen, Donor 302C, 24 h) in which fewer than 40% of reads were mapped to the transcriptome was removed from further analysis (Additional file 1: Figure S3a). Count matrices were analyzed by the scanpy version 1.4 [42] tool in Python version 3.7.2. Cells with less than 300 or more than 5000 detected genes (8000 in esophagus), more than 20,000 UMI, and more than 10% mitochondrial reads were removed. Genes that were detected in less than three cells per tissue were removed. All donors and time points per tissue were combined for analysis. The reads were log-transformed and normalized.

#### Quality metrics of samples

Number of cells, number of reads, median genes per cell, reads confidently mapped to the transcriptome, and other quality metrics were obtained from Cell Ranger’s output metrics.csv files. The confidently mapped reads to intronic, exonic, and intergenic regions were further studied by extracting the number of reads mapping confidently (QC = 225 from Cell Ranger’s output bam file) for every cell barcode.

“Scrublet” [43] was used to calculate the doublet scores for each cell in every 10x separately.

#### Unique molecular identifier (UMI) count analysis

The number of UMIs in each droplet was quantified by using soupX tool [37] in R. UMI counts were normalized for read depth by multiplying with 1 million and dividing by the sum of UMI in all droplets: normalized UMI = UMI per droplet × 1,000,000/UMI in all droplets per run. Three intervals were defined for describing the distribution of reads: 0 < ambient RNA ≤ 0.25, 0.25 < debris ≤ 5, and 5 < cellular material. The droplets containing up to 0.25 normalized UMI were defined as ambient RNA expression originating from free-floating RNA in the sample.

#### Clustering and annotation of cell types

To achieve good clustering by cell types, number of counts, mitochondrial percentage, and donor effects were regressed out. PCA was carried out on highly variable genes, and the donor effect was reduced by BBKNN tool [44]. Leiden clustering [45] and UMAP visualization were performed for gaining clusters of cells and visualization. Statistical analysis was performed in R version 3.5.0, and plotting was in Python via scanpy or custom script and in R using ggplot2 version 2.2.1 or by using custom scripts. Cells which contained more than 10% mitochondrial reads were assigned by similarity to their closest cell type within a tissue with scmap tool [46], using cells with less than 10% mitochondrial reads as a reference. The high and low mitochondrial percentage cells were then combined for calculating the mitochondrial percentage per each cell type. All code for the analysis is available at https://github.com/elo073/TissStab.

Expression of known markers and re-analysis of bigger clusters were used to annotate cell types, with cell markers shown in Additional file 1: Figure S9. The major cell types were annotated for the lung, esophagus, and spleen by looking at expression of known cell type markers. Three subsets from the lung (mononuclear phagocytes and plasma cells; lymphocytes; dividing cells), two subsets from the esophagus (immune; small clusters), and two subsets from the spleen (DC, small clusters and dividing cells; CD4 and CD8 T cells) were extracted, further analyzed by re-clustering, and annotated using known markers. These updated annotations then replaced the original bigger ones.

#### Explanatory variance calculation

Effect of variable factors (donor, tissue, time point, cell type, n_counts, etc.) on gene expression was studied by the scater package by computing the marginal *R*^2^ that describes the proportion of variance explained by each factor alone for each gene. Density plots of the gene-wise marginal *R*^2^ are shown. Normalized and scaled gene expression was used with the effect of donor and number of counts regressed out, but not mitochondrial percentage or time. Effect of time only as a continuous variable was calculated for each cell type or cell type group in tissues. Smaller or similar cell types were combined to groups as Endothelial (Blood vessel, Lymph vessel), Alveolar (Alveolar Type 1 and Type 2), Mono_macro (Monocyte, Macrophage_MARCOneg, Macrophage_MARCOpos), and T_cell (T_CD4, T_CD8_Cyt, T_regulatory) in the lung, and Mono_macro (Monocyte, Macrophage), NK (NK_FCGR3Apos, NK_CD160pos), T_cell (T_CD4_conv, T_CD4_fh, T_CD4_naive, T_CD4_reg, T_CD8_activated, T_CD8_CTL, T_CD8_gd, T_CD8_MAIT-like, T_cell_dividing), and B_cell (B_follicular, B_Hypermutation, B_mantle) in the spleen.

#### Differential expression

The Wilcoxon signed-rank test was used to compare expression between time points in bulk RNA-sequencing samples, and between bulk RNA-sequencing and sc-pseudo-bulk samples. The Benjamini and Hochberg (BH)-corrected *p* values were reported, as well as the median_log2_foldchange.

#### Test of independence

Fisher’s exact test was performed for overrepresentation of time signature genes (Additional file 7: Table S6) among the dissociation-related genes with adjusted *p* value < 0.01 and median log2 fold change < − 2 (Additional file 5: Table S4) in all three tissues.

#### Analysis of whole genome sequencing (WGS)

Pair-end WGS data of 30× were mapped to GRCh38 using bwa-mem [47]. After removing duplicates and filtering out reads with mapping quality < 30, single nucleotide variants (SNVs) were called using bcftools-mpileup and bcftools-call [48] for each sample individually and filtered using the criteria “DP>10 && DP<70 && QUAL>221 && MQB>0.5 && MQSB>0.5 && RPB>0.05”. The resulting SNV call set yields a Ts/Tv of 2.1 for all samples. For comparison, SNV call set on GRCh38 from 1000 genomes project [49] were downloaded from 1K genome project data FTP. Functional consequences of sample SNVs and 1K genome SNVs were predicted using bcftools-csq [48] based on EnsEMBL gene annotation v98. Copy number variations (CNVs) were called using ERDS [50] for each sample individually using the default parameters. We only kept calls at least 1 kb in size and not overlapping centromere or gaps in the reference genome. For comparison, the latest DGV [51] (database of genomic variants) gold standard variant sets were downloaded from DGV, and similarly, only variants at least 1 kb in size were considered. For whole genome visualization of CNVs, average depth of 50 kb non-overlapping genomic bins was calculated for all samples and was normalized by the median across the samples of the same sex.

## Supplementary information

**Additional file 1:** Supplementary Table legends and supplementary Figures.

**Additional file 2: Table S1.** Patient characteristics and sample information.

**Additional file 3: Table S2.** Number of cells per cell types.

**Additional file 4: Table S3.** RIN-values for samples.

**Additional file 5: Table S4.** Differential Expression between the bulk and single-cell pseudo-bulk RNA-sequencing samples.

**Additional file 6: Table S5.** Changes in cell-type proportions between time points and donors.

**Additional file 7: Table S6.** Pairwise Differential Expression between time points with bulk RNA-sequencing data in three tissues.

**Additional file 8: Table S7.** Explained variability by time in different cell types.

**Additional file 9:** Review History.

## Data Availability

The datasets generated in this study are available through the Human Cell Atlas Data Coordination Platform and NCBI BIOPROJECT accession code PRJEB31843 (https://prod.data.humancellatlas.org/explore/projects/c4077b3c-5c98-4d26-a614-246d12c2e5d7) [54]. The data can also be browsed interactively at www.tissuestabilitycellatlas.org. All experimental protocols are available on protocols.io, https://www.protocols.io/ [40].
