# Supplementary material for: scRNA-seq assessment of the human lung, spleen, and esophagus tissue stability after cold preservation
Source: Genome Biol. 2019 Dec 31;21:1. doi: 10.1186/s13059-019-1906-x (PMC6937944; doi:10.1186/s13059-019-1906-x)
Supplement: Supplementary file 1 — Additional file 1: Supplementary Table legends and supplementary Figures. [file 13059_2019_1906_MOESM1_ESM.docx]

Additional file 1

##

## **scRNA-seq assessment of human lung, spleen and esophagus tissue stability after cold preservation**

Madissoon, E.^1, 2^*, Wilbrey-Clark, A^1^.*, Miragaia, R.J^1^., Saeb-Parsy, K.^3^, Mahbubani, K.T.^3^, Georgakopoulos, N.^3^, Harding, P.^1^, Polanski, K^1^., Huang, N^1^., Nowicki-Osuch, K^4^., Fitzgerald, R. C.^4^, Loudon, K.W.^5^, Ferdinand, J.R.^5^, Clatworthy, M.R^5^, Tsingene, A.^1^, Van Dongen, S.^1^, Dabrowska, M.^1^, Patel, M.^1^, Stubbington, M.J.T.^1,6^, Teichmann, S.A.^1^, Stegle, O.^2^, Meyer, K.B.^1†^

# Table of contents

[scRNA-seq assessment of human lung, spleen and esophagus tissue stability after cold preservation](#_bgoet54ch61c) 1

[**Table of contents**](#_oq77qsnxrmzn) **2**

[**Supplementary Table legends**](#_sebkzr7vuqyx) **3**

[Table S1. Patient characteristics and sample information.](#_blhhfntvtydn) 3

[Table S2. Number of cells and proportions per cell types.](#_go4ut3s00ubw) 3

[Table S3. RIN-values for samples.](#_v8wfh13uobb9) 3

[Table S4. Differential Expression between the bulk and single-cell pseudo-bulk RNA-sequencing samples.](#_18t0x09jtvuj) 3

[Table S5. Changes in cell-type proportions between time points and donors.](#_ytf4y81nzv30) 3

[Table S6. Pairwise Differential Expression between time points with bulk RNA-sequencing data in three tissues.](#_x7cjwzf6d6to) 4

[Table S7. Explained variability by time in different cell types.](#_2kblzg8ljq6l) 4

[**Supplementary Figures**](#_whc00k5ny9hv) **5**

[Fig S1. Donors have no gross genomic abnormalities.](#_m64m7lpvnl4z) 6

[Fig S2. Histological analysis.](#_ayh5h3afqka6) 8

[Fig S3. scRNA-seq quality metrics for all samples.](#_bug4h0fglr6h) 9

[Fig S4. Fraction of reads in exonic versus intronic regions changes in time in spleen, esophagus and lung.](#_fj1vafxx4jtq) 10

[Fig S5. Doublet score predictions do not change with storage time.](#_70feeid852q5) 11

[Fig S6. Proportions of droplets in three tissues and intervals.](#_7l2dd74u1n28) 12

[Fig S7. Cell viability directly after dissociation and after dead cell removal.](#_n2gtlo2kd3nu) 13

[Fig S8. TUNEL staining](#_3rovbtpualak) 14

[Fig S9. Cell type markers and their expression in the data.](#_fyma7edvcn1y) 17

[Fig S10: Distribution of cells from different donors.](#_uh675hz3jfi) 18

[Fig S11. Expression of Club cell markers in lung.](#_836kc03s9dny) 19

[Fig S12. Bulk RNA-sequencing data comparison with single-cell RNA-sequencing.](#_uykyjxok83ap) 20

[Fig S13: Proportions of cells types shown per donors or time.](#_xni0iq8c319r) 22

[Fig S14: Change in the proportion of cell types in time.](#_p8o0pkoth9lj) 24

[Fig S15. Time explains the least of variance in gene expression.](#_w1tbzc8vjhak) 25

[Fig S16. Mitochondrial percentage differs between cell types.](#_2hok8upu14is) 26

[Fig S17. Change in the percentage of mitochondrial reads with time and by cell type and donor in spleen.](#_aikp4h71ip0) 27

[Fig S18. Gene signatures associated with storage time are correlated with tissue type and not cell type.](#_w3yuofznn5v) 28

[Fig S19. Frequency plots of top ambient RNA contamination genes.](#_w9irsfmmunh8) 29

#

# Supplementary Table legends

#### **Table S1. Patient characteristics and sample information.**

Sheet entitled “Donor metadata”: DCD = Donation after cardiac death, DBD = Donation after brainstem death, NRP = normothermic regional perfusion. Additional metadata is available in the Data Coordination Platform submission, or upon request.

Sheet entitled “Sample info”: Information regarding scRNAseq sample ID, corresponding organs, donors and timepoints.

#### **Table S2. Number of cells and proportions per cell types.**

Number of cells as well as cell proportions are given for each cell type in every sample according to the tissues. Samples are shown in columns, where each time point is an individual sample, labelled by time and donor (time_donor), cell types are shown in rows. Proportions are also given per donor and per time point. Tissues are distributed in three separate sheets of the workbook: Lung, Esophagus and Spleen.

#### **Table S3. RIN-values for samples.**

Quality of the samples was assessed by the RNA Integrity number (RIN) measured by Agilent Bioanalyser. RIN values are shown and plotted for all the samples that were analysed.

#### **Table S4. Differential Expression between the bulk and single-cell pseudo-bulk RNA-sequencing samples.**

Single-cell pseudo-bulk (sc-pseudo-bulk) samples were compared with their corresponding bulk RNA-sequencing data. Differential gene expression was calculated and values were subjected to the Wilcoxon signed-rank test. P-values and Bonferroni corrected FDR p-values are shown in columns “pvals” and “pvals_adj”, respectively. Median log2 fold-change is shown for every gene in the column “median_log2_foldchange”. Positive values imply upregulation in the first group listed, negative values imply upregulation in the second group tested. The four sheets in the workbook display results for four different comparisons: Lung bulk vs sc-pseudo-bulk, Esophagus bulk vs sc-pseudo-bulk, Spleen bulk vs sc-pseudo-bulk and all tissues combined bulk vs sc-pseudo-bulk.

#### **Table S5. Changes in cell-type proportions between time points and donors.**

Sheet entitled “TIME.t.test.celltype.proportion”: student’s t-test and fold change analysis was performed to compare cell type proportion changes between time points. Five comparisons were made: T0 versus 72h (0.vs.72); T0 versus 24h (0.vs.24); 24h versus 72h (24.vs.72); T0 and 12h versus 24h and 72 (0.12.vs.24.72); T0 and 12h and 24h versus 72h (0.12.24.vs.72). Fold changes are given in comparison to the earlier time point(s) with larger values indicating higher proportions in earlier time point(s). Adjusted p-values (Benjamini Hochberg) are given as separate columns.

Sheet entitled “DONOR.anova.celltype proportion”: one-way analysis of variance (ANOVA) was performed on the means of cell type proportions in different samples using donor as the grouping variable. The test was performed for each cell type in each tissues to test whether at least one donor is not equal to the others. The resulting p-values as well as BH corrected p-values are given in separate columns.

#### **Table S6. Pairwise Differential Expression between time points with bulk RNA-sequencing data in three tissues.**

Wilcoxon signed-rank test was performed between the Clinic time point (“true 0h”) and all other time points (T0, 12h, 24h, 72h) in lung, and between T0 and all other time points (12h, 24h, 72h) in lung, esophagus and spleen shown in four different workbook sheets. P-values and Bonferroni corrected FDR p-values are shown in columns “pvals” and “pvals_adj” correspondingly. Median log2 fold-change is shown for every gene in column “median_log2_foldchange”. Positive values imply upregulation in the first group listed, negative values imply upregulation in the second group tested.

#### **Table S7. Explained variability by time in different cell types.**

Proportion of variance explained by time was calculated for each cell type or cell type group and is shown for genes that explained >= 1% of the variance in time in any of the cell types or cell type groups. Cell types were grouped as follows: Endothelial (blood vessel, lymph vessel), Alveolar (alveolar Type 1 and Type 2), Mono_macro (Monocyte, Macrophage_MARCOneg, Macrophage_MARCOpos), T_cell (T_CD4, T_CD8_Cyt, T_regulatory) in Lung and Mono_macro (Monocyte, Macrophage), NK (NK_FCGR3Apos, NK_CD160pos), T_cell (T_CD4_conv, T_CD4_fh, T_CD4_naive, T_CD4_reg, T_CD8_activated, T_CD8_CTL, T_CD8_gd, T_CD8_MAIT-like, T_cell_dividing) and B_cell (B_follicular, B_Hypermutation, B_mantle) in Spleen.

##

# Supplementary Figures

a b


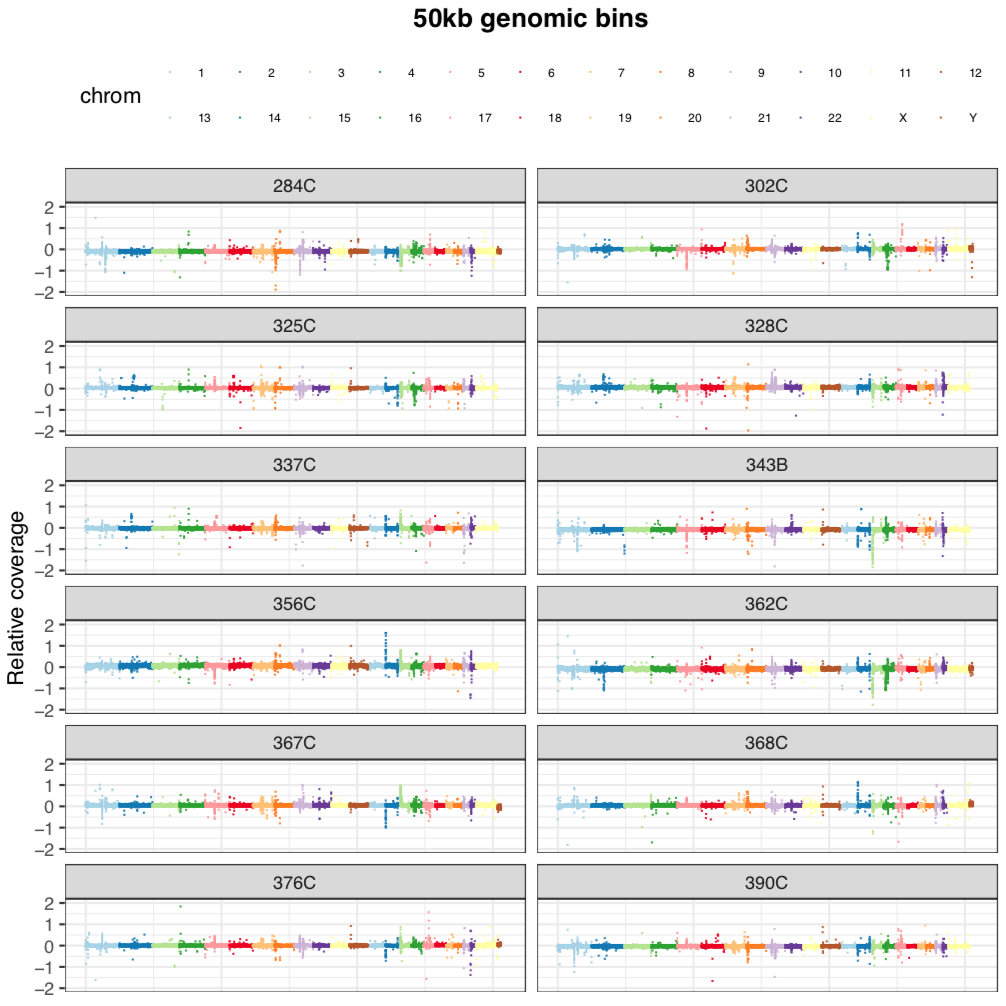

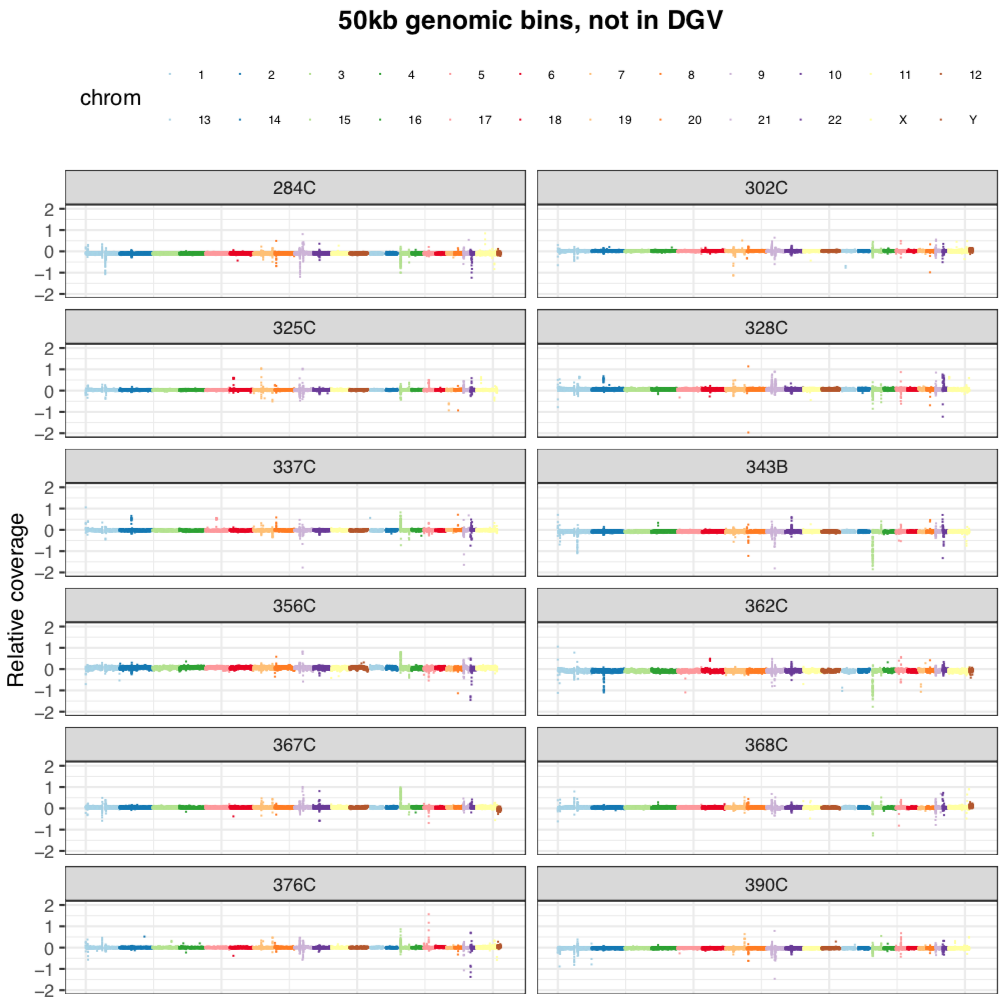


c


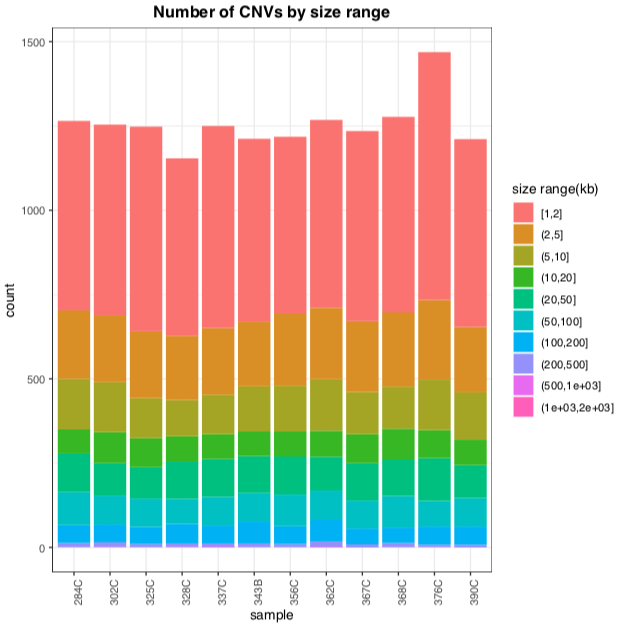

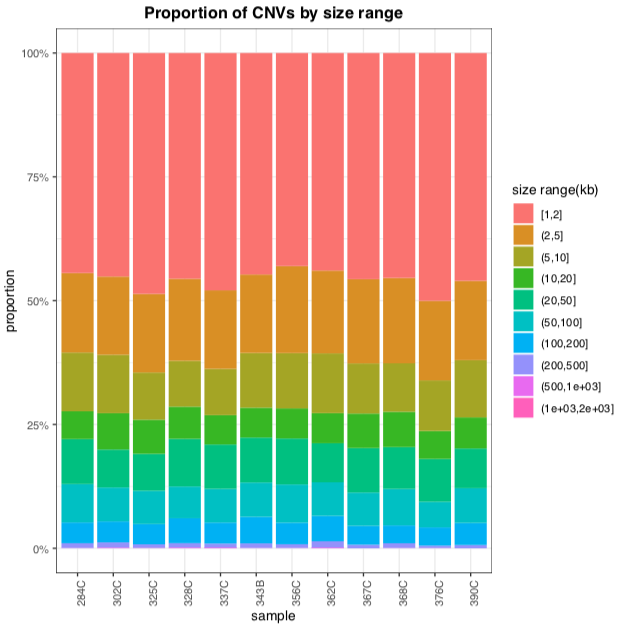

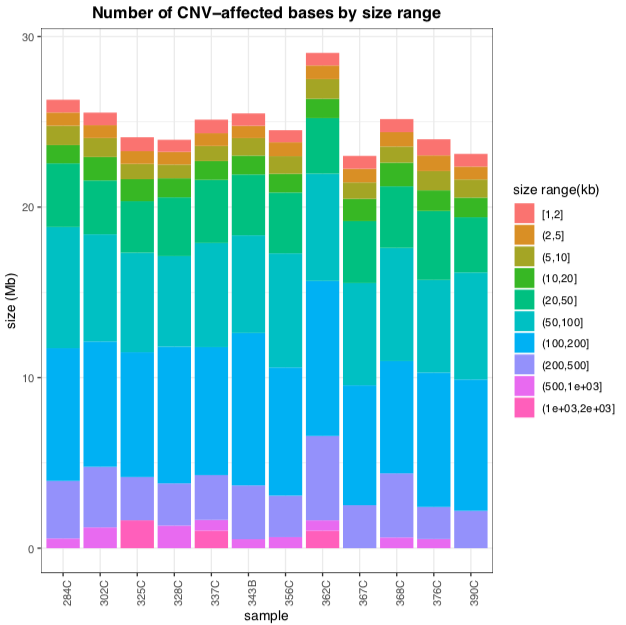

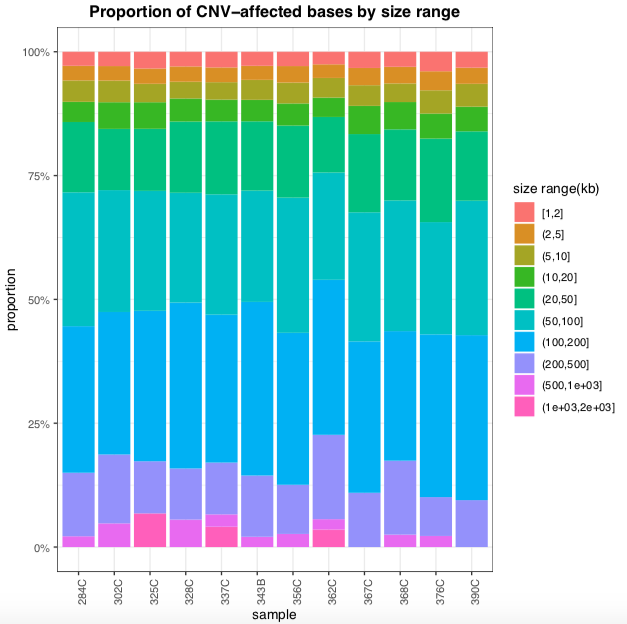


d


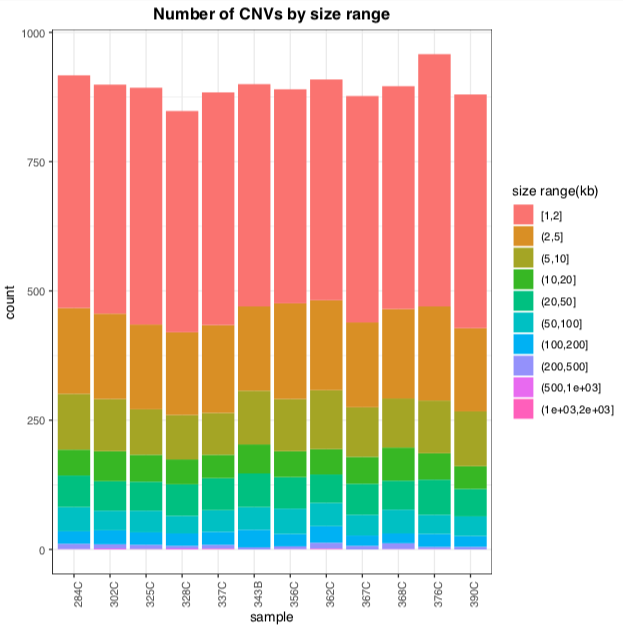

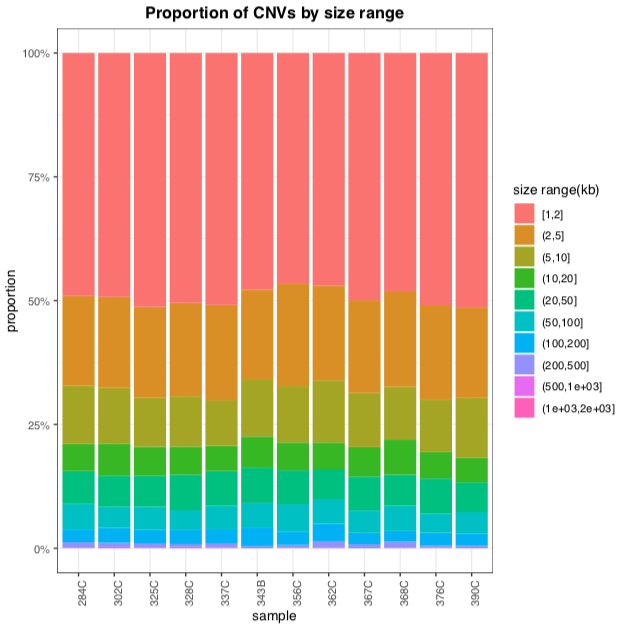

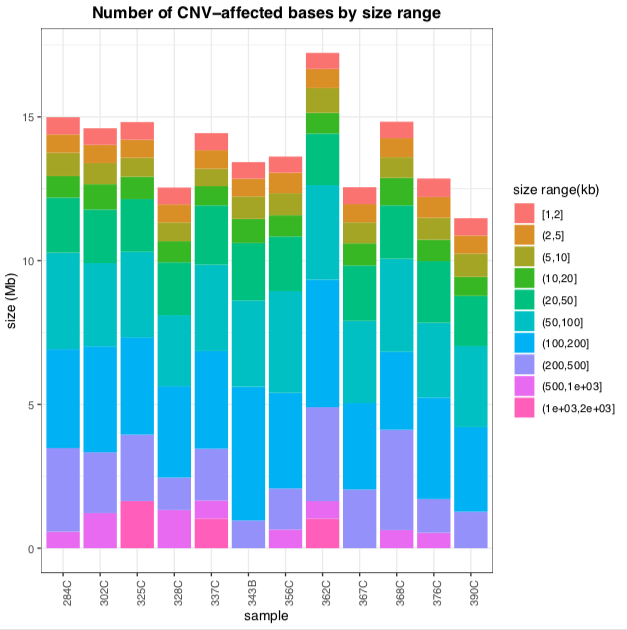

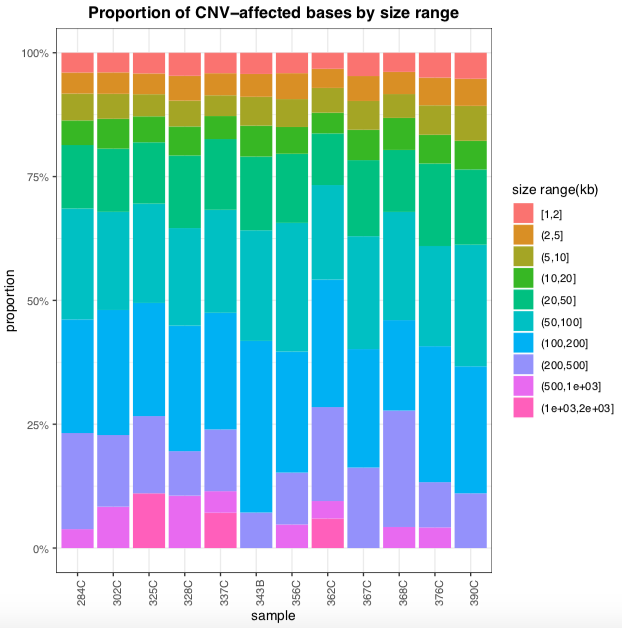


e


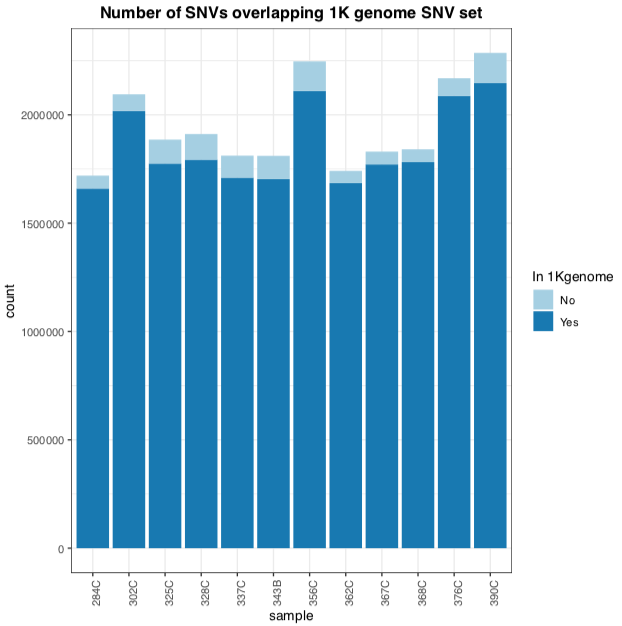

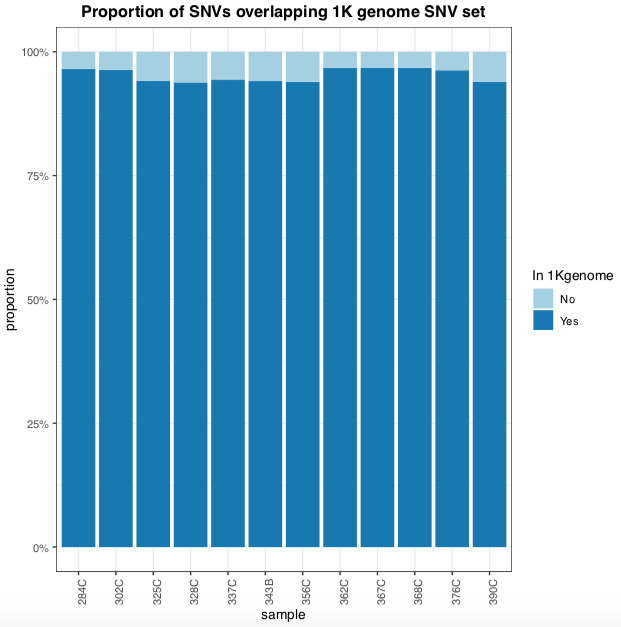

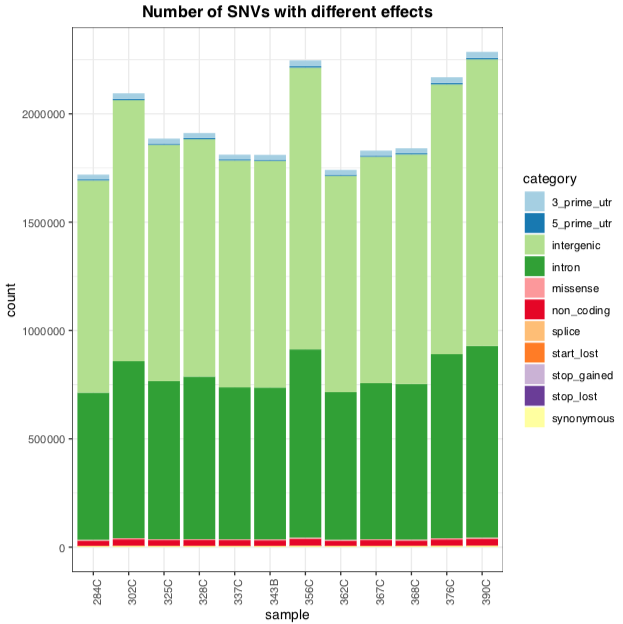

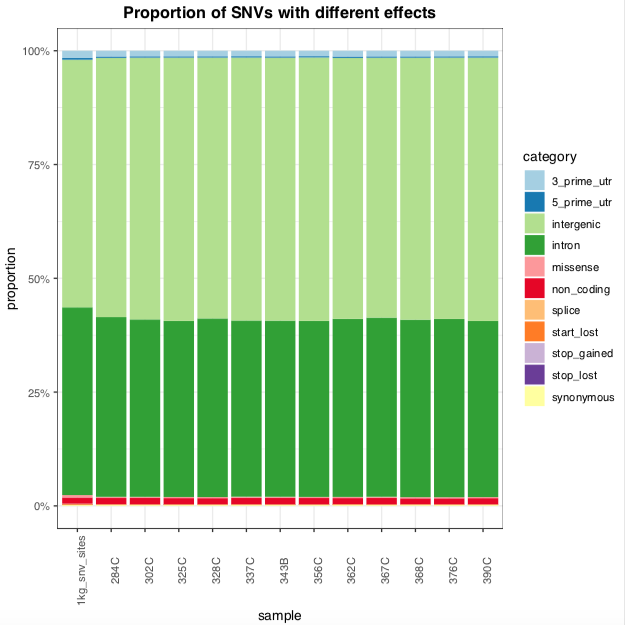


#### **Fig S1. Donors have no gross genomic abnormalities.**

Normalised read depth (log2 ratio relative to the median of all donors of the same sex) of each chromosome and donor at all 50kb genomic bins (a) and those not overlapping DGV (data base of genome variation) gold-standard CNV set from apparently healthy individuals (b). Number of CNVs and CNV affected bases and their proportions broken-down by CNVs size ranges for each donor including (c) and excluding (d) CNVs overlapping those in DGV gold-standard CNV set from apparently healthy individuals. Number and proportion of SNVs overlapping 1K genome SNV set and with different predicted functional effects (e).


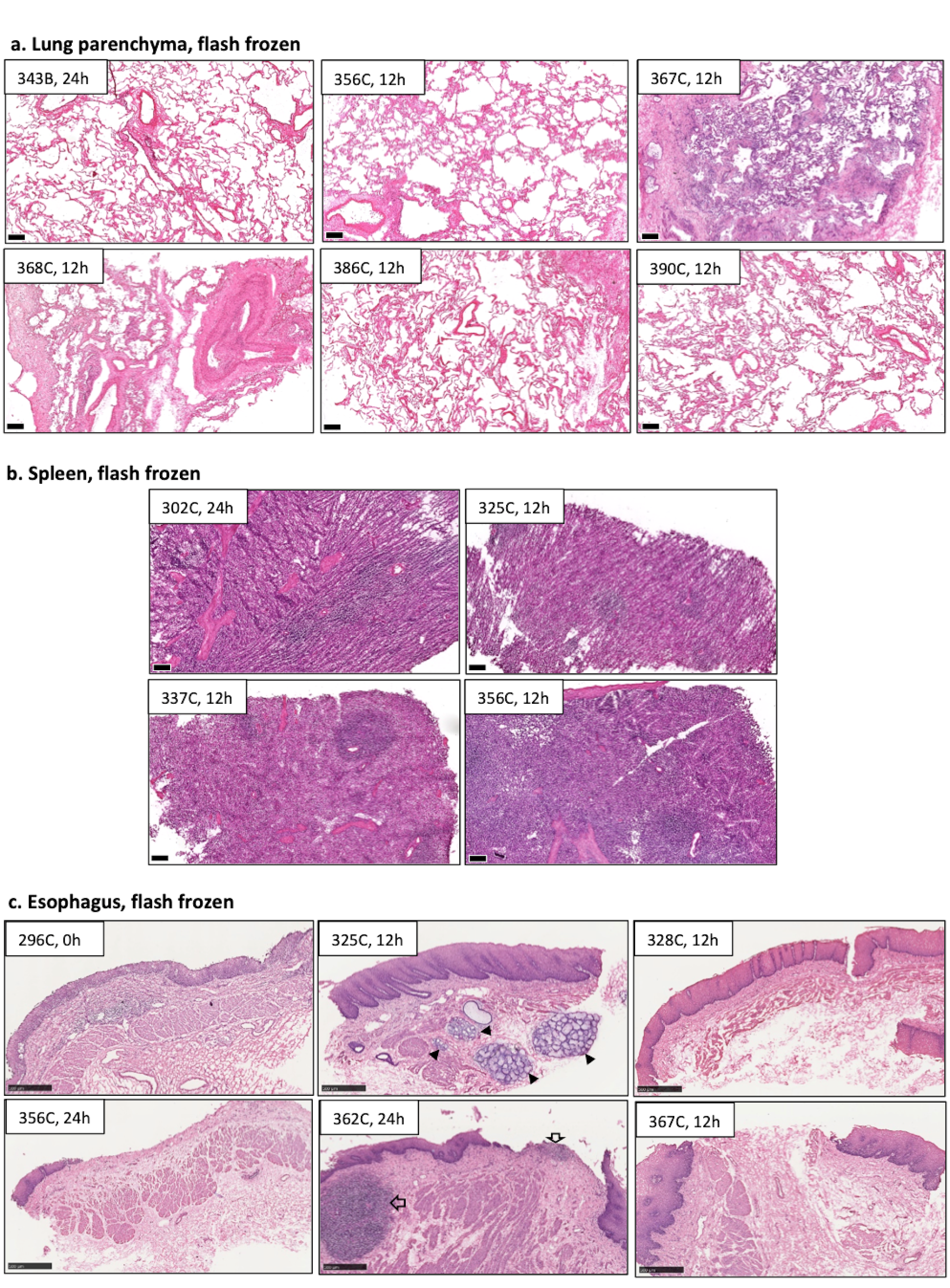


#### **Fig S2. Histological analysis.**

Flash-frozen tissue samples from the time points indicated were cryosectioned and stained with haematoxylin and eosin for (**a**) lung, (**b**) spleen and (**c**) esophagus. This shows normal, healthy histology overall (some emphysema is apparent in lung samples, but this is frequently observed in healthy patients). Of note, the lung from donor 368C exhibits some evidence of lung hypertension. A large vessel is apparent in the section taken from donor 368C. Note that apart from donor 296C, for which a full cross-section was retained, esophagus samples contain only the mucosa / epithelium (the same layer used for single cell / bulk sequencing). Though damaged during processing in some samples, epithelium is clearly visible as a dark purple layer along the outer edge of the tissue. Esophagus donor 325C, 12h, contains glands (arrow heads) and donor 362C, 24h has evidence of inflammation of unknown origin (open arrows). All images at 5x magnification; scale bar = 200μM for lung and spleen, 500μM for esophagus.


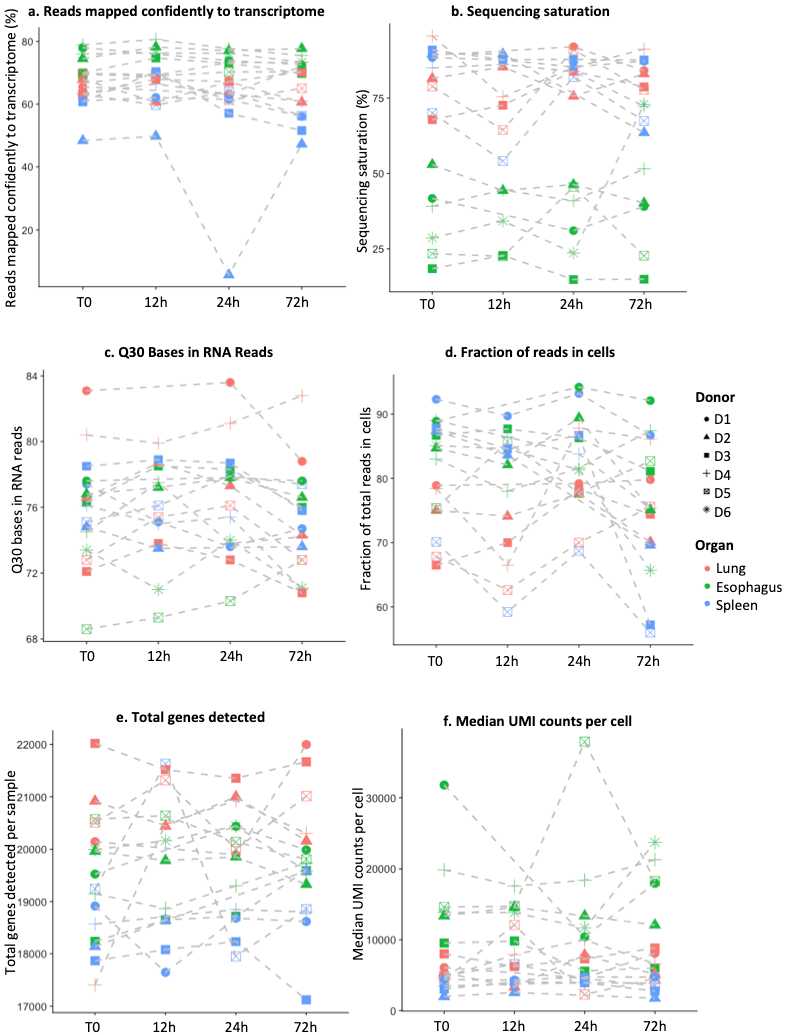


#### **Fig S3. scRNA-seq quality metrics for all samples.**

Percentage of reads mapping confidently to the transcriptome (QC=225 from Cellranger 2.0.2 pipeline mapping the reads to GRCh38 1.2.0 Human Genome reference) shows an outlier with less than 40% (donor 2 for lung at time point 24h) and was removed from analysis (a). Percent sequencing saturation (b), Q30 bases in RNA reads (c), fraction of total reads assigned to cells (d), total number of genes detected per sample (e), median number of UMI counts per cell (f). Samples are colored by tissue, shapes correspond to a separate donor within the tissue.

#### **
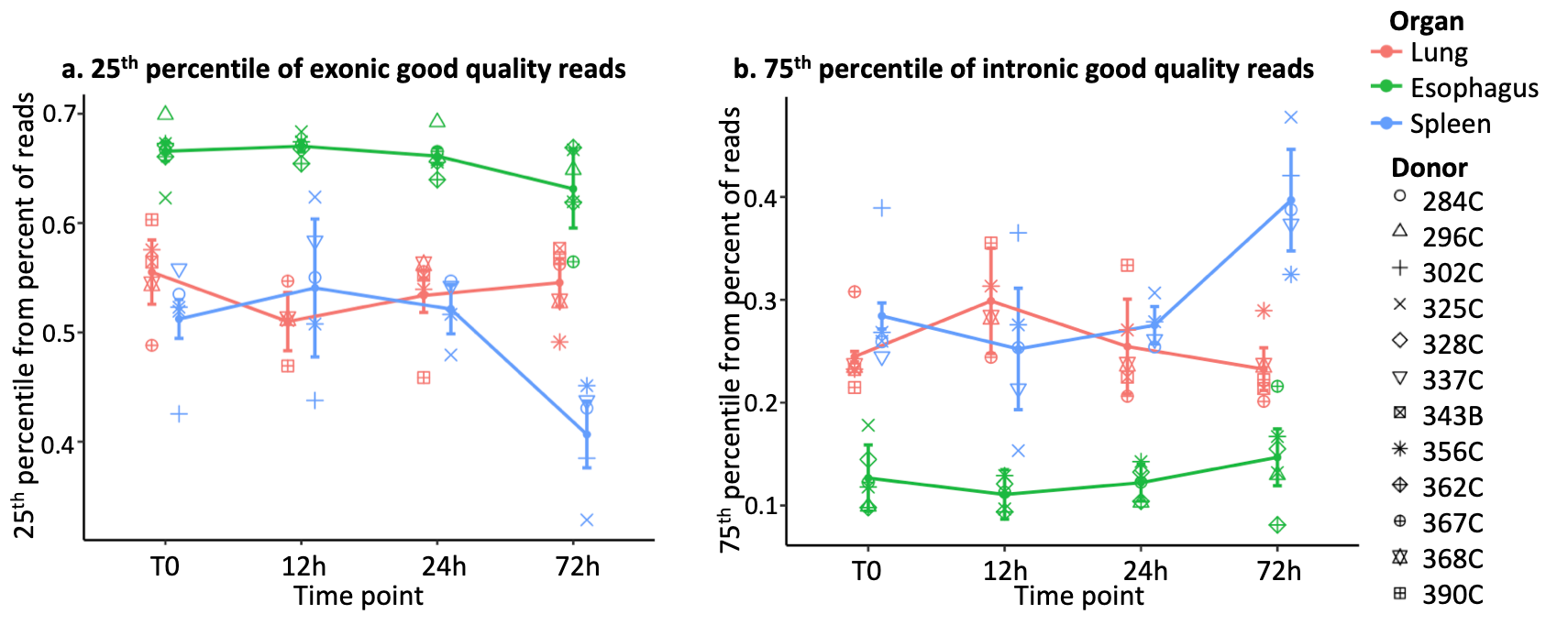
**

#### **Fig S4**. **Fraction of reads in exonic versus intronic regions changes in time in spleen, esophagus and lung.**

Percentage of good quality reads mapping to exons (a) or introns (b) in 25% or 75% of cells correspondingly. p-value is significant between T0 and 72h only in spleen for both exonic (p-value = 0.007) and intronic (p-value = 0.013) mapping fractions.


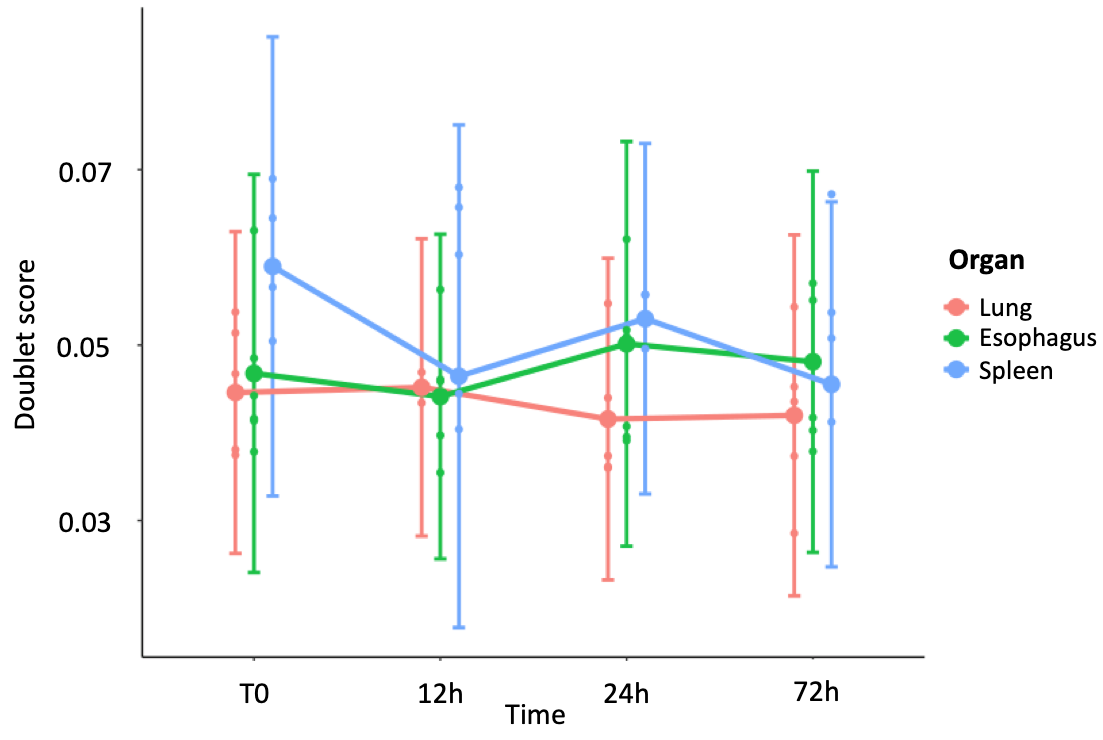


#### **Fig S5**. **Doublet score predictions do not change with storage time.**

Scrublet was applied per every sample separately, doublets were simulated and doublet scores calculated. Mean values of doublet scores per run are plotted for each time point and tissue. Different tissues are shown by different colors, standard deviation is indicated by whiskers. Highest fold change is observed between T0 and 72h in spleen, providing a p-value of 0.24.


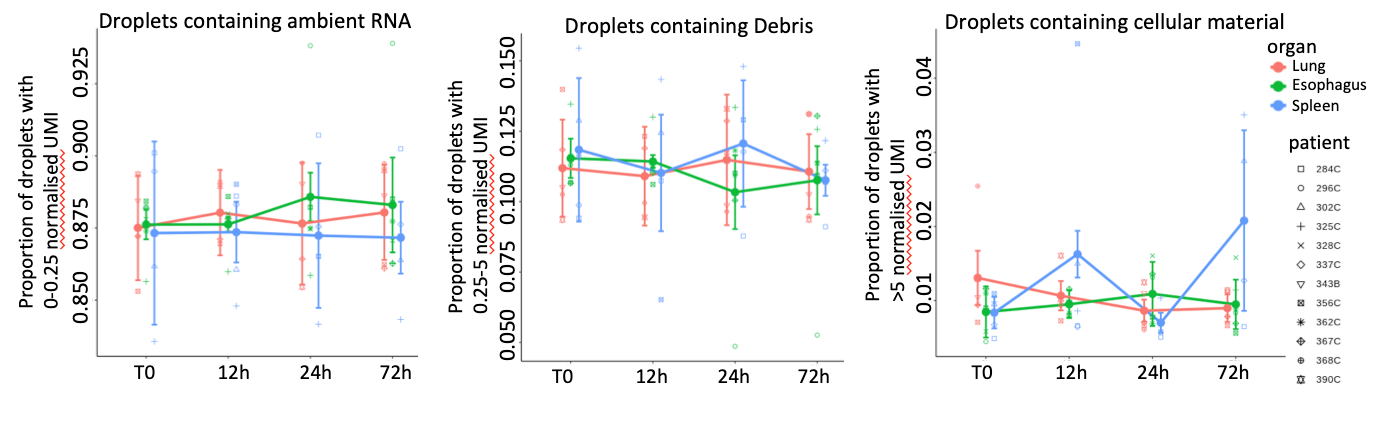


#### **Fig S6**. **Proportions of droplets in three tissues and intervals.**

Proportion of droplets containing normalised UMI for all three tissues in given intervals: ambient RNA with less than 0.25 normalised UMI (a), debris from 0.25, but less than 5 normalised UMI (b) and cellular material with over 5 normalised UMI (c). Mean values and standard deviations are indicated by errorbar for donors within each tissue and timepoint, line types connect means within the same tissue. Color indicates tissues, shapes indicate donors.

#### **
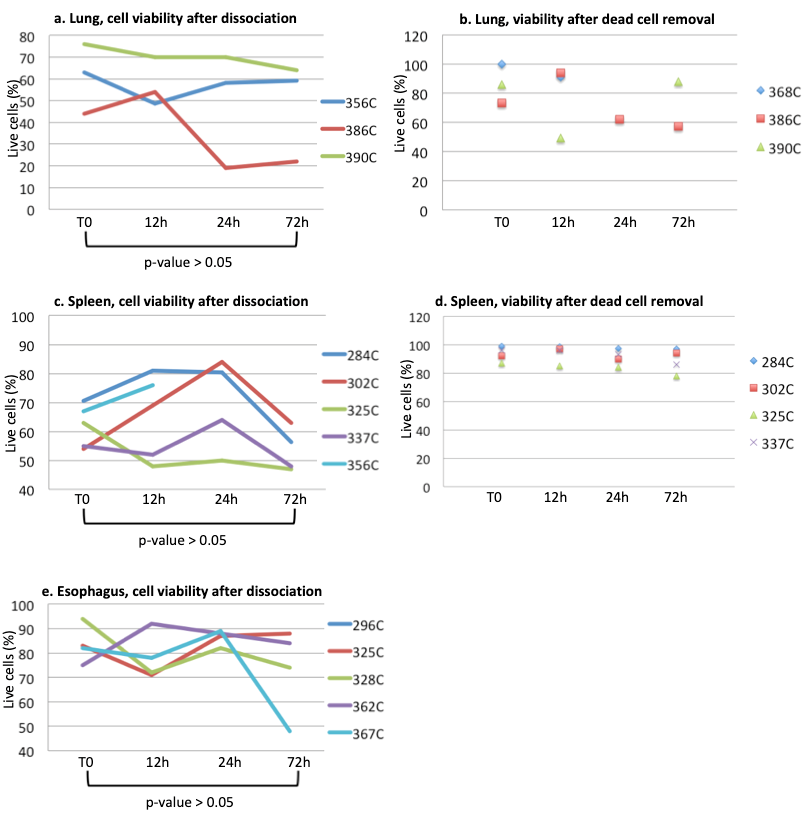
**

#### **Fig S7**. **Cell viability directly after dissociation and after dead cell removal.**

Cell viabilities are shown for lung (a, b) and spleen (c, d) both directly after dissociation (a, c) and after dead cell removal (b, d), and for esophagus only directly after dissociation (e). Student’s t-tests were performed in each tissue for viability percentages directly after dissociation between T0 and 72h.


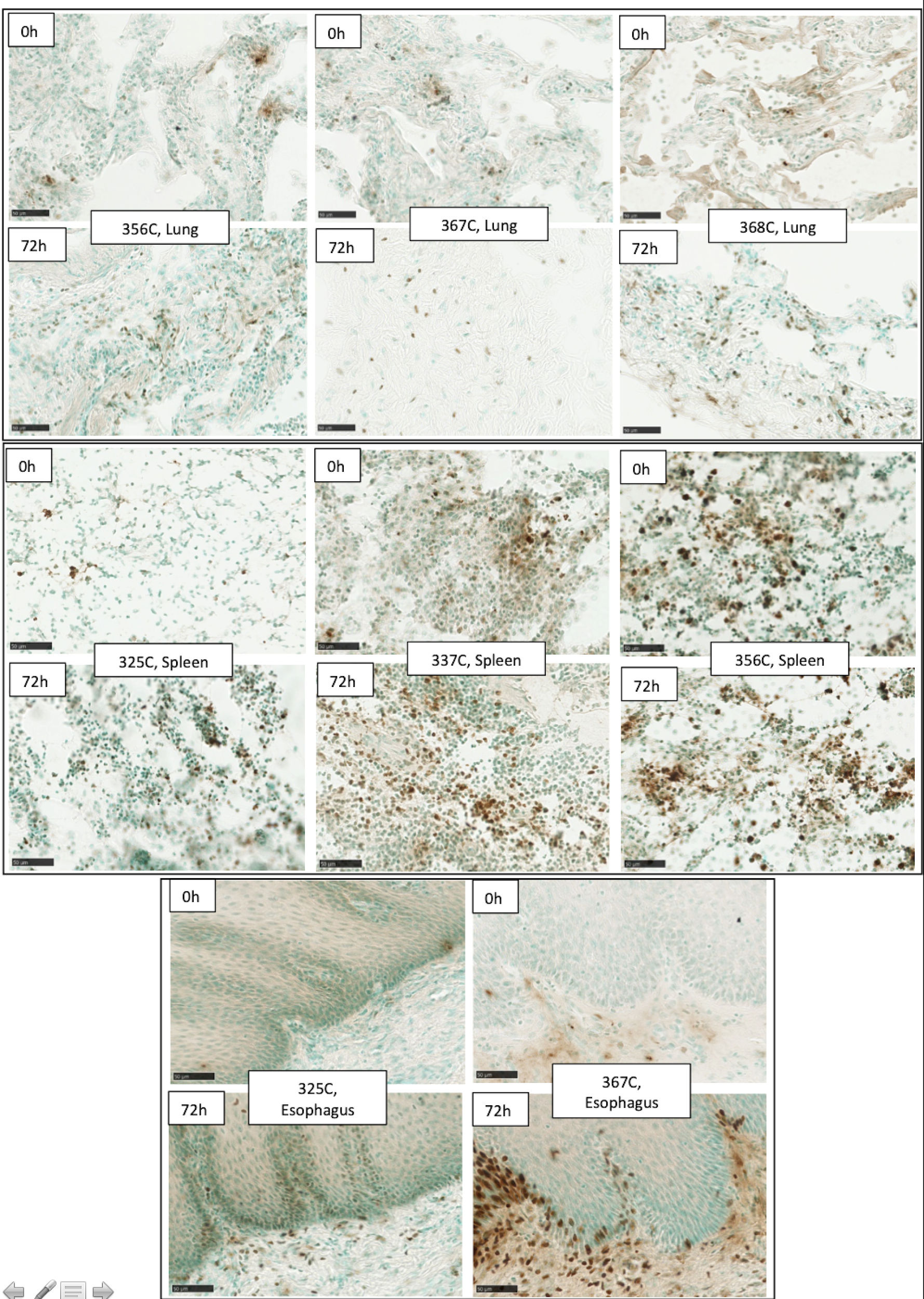


#### **Fig S8**. **TUNEL staining**

TUNEL staining of fresh frozen tissue sections from 0h and 72h time points for lung (n=3), spleen (n=3) and esophagus (n=2). Positive staining (DNA strand breaks) is indicated by brown nuclei; methyl green nuclear counterstain. All images at 40x magnification, scale bars = 50µM.


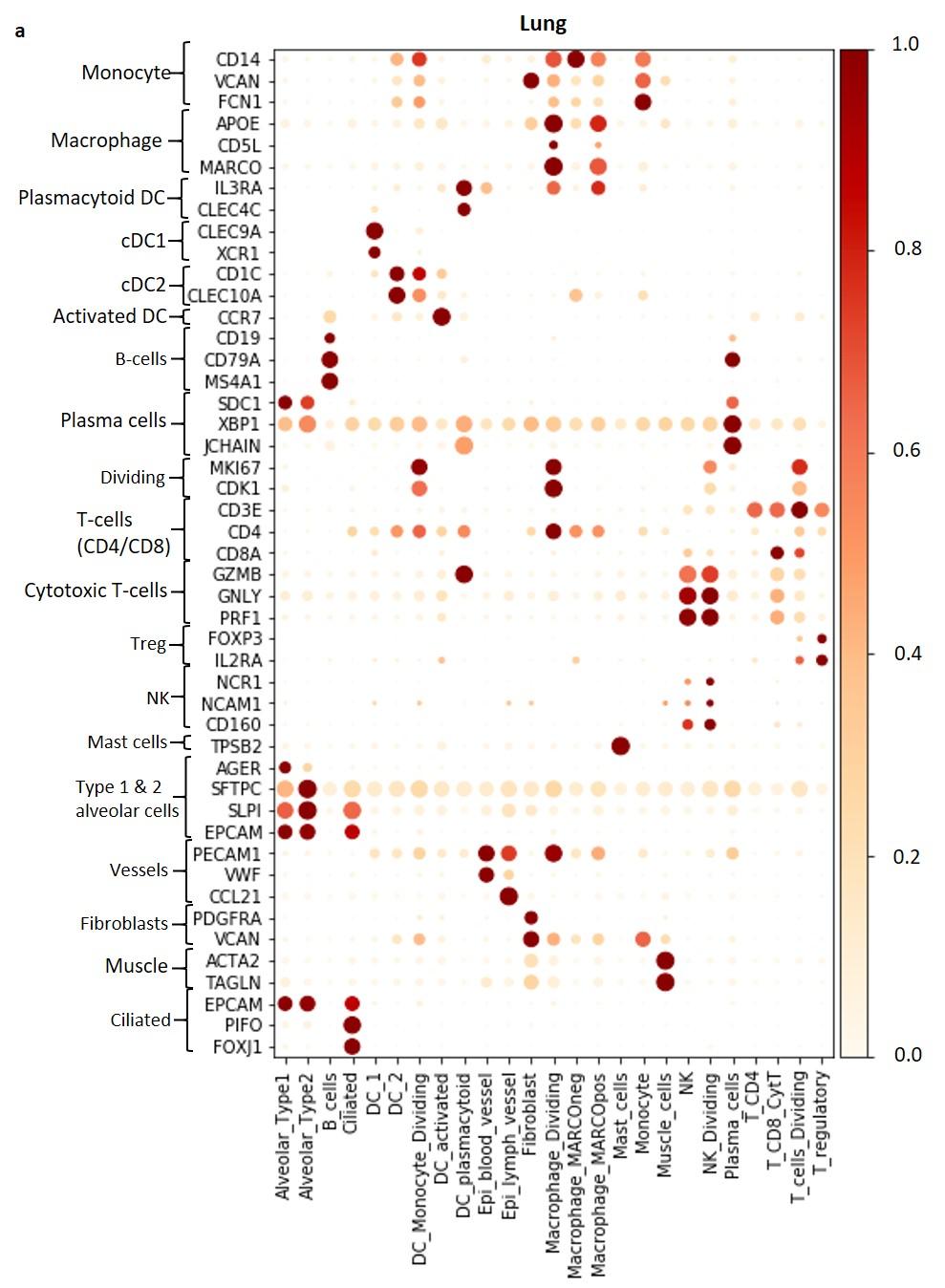


**
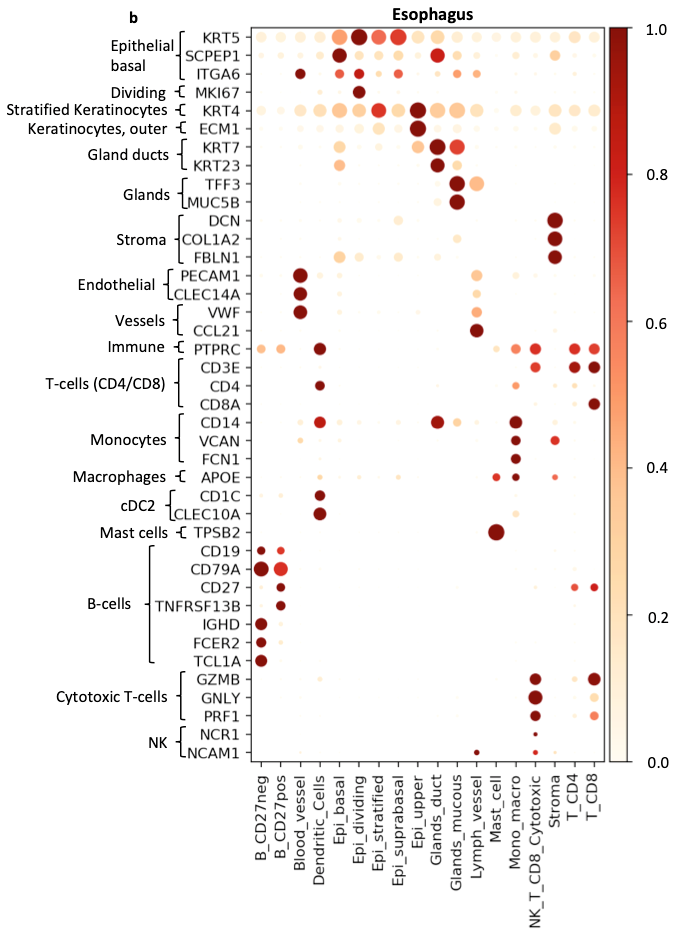
**

#### **
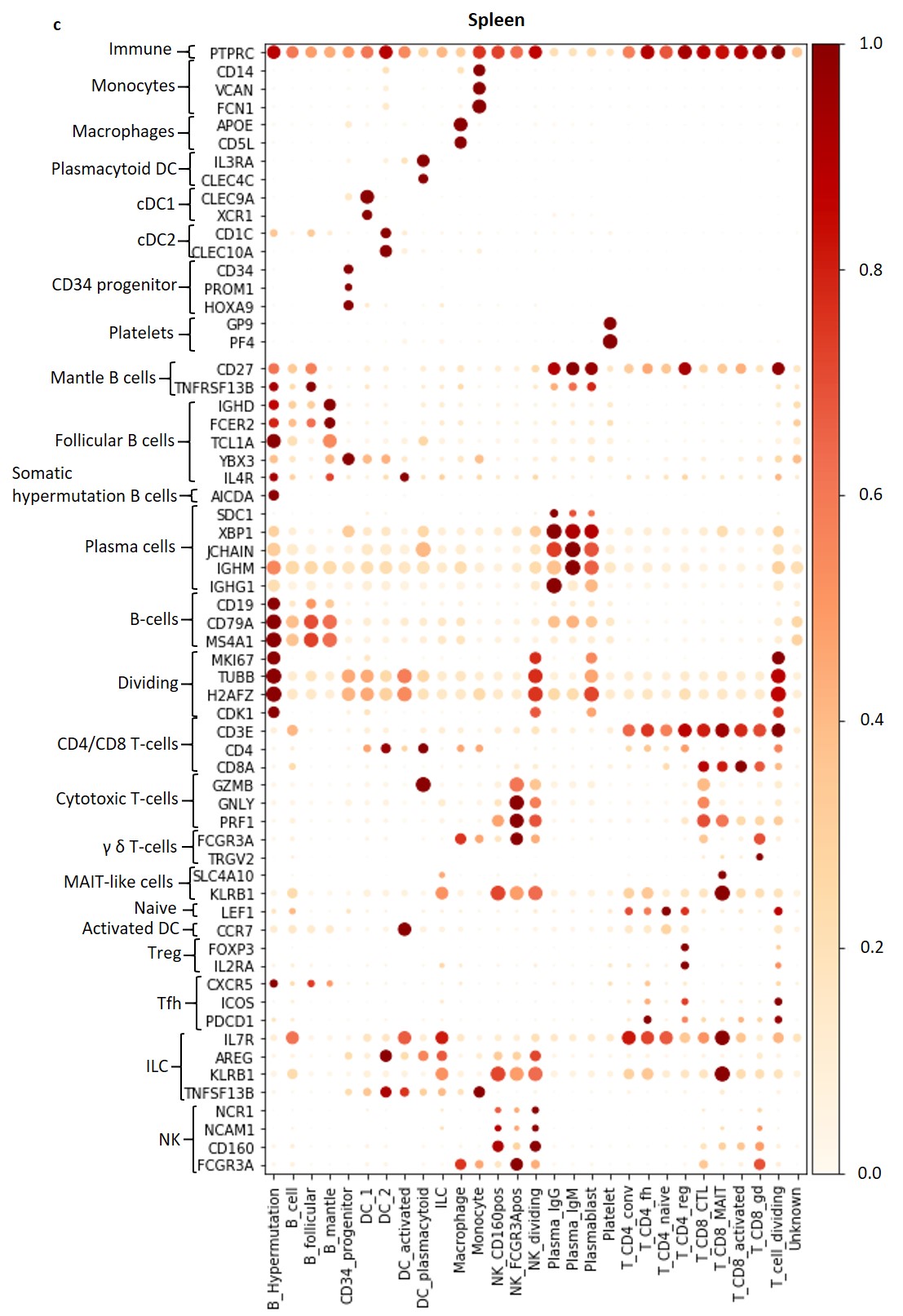
** **Fig S9. Cell type markers and their expression in the data.**

Overview of Lung (a), Esophagus (b) and Spleen (c) single-cell RNA-sequencing data cell type markers genes expression. Color represents maximum-normalised mean expression of marker genes in each cell group, and size indicates the proportion of cells expressing marker gene.


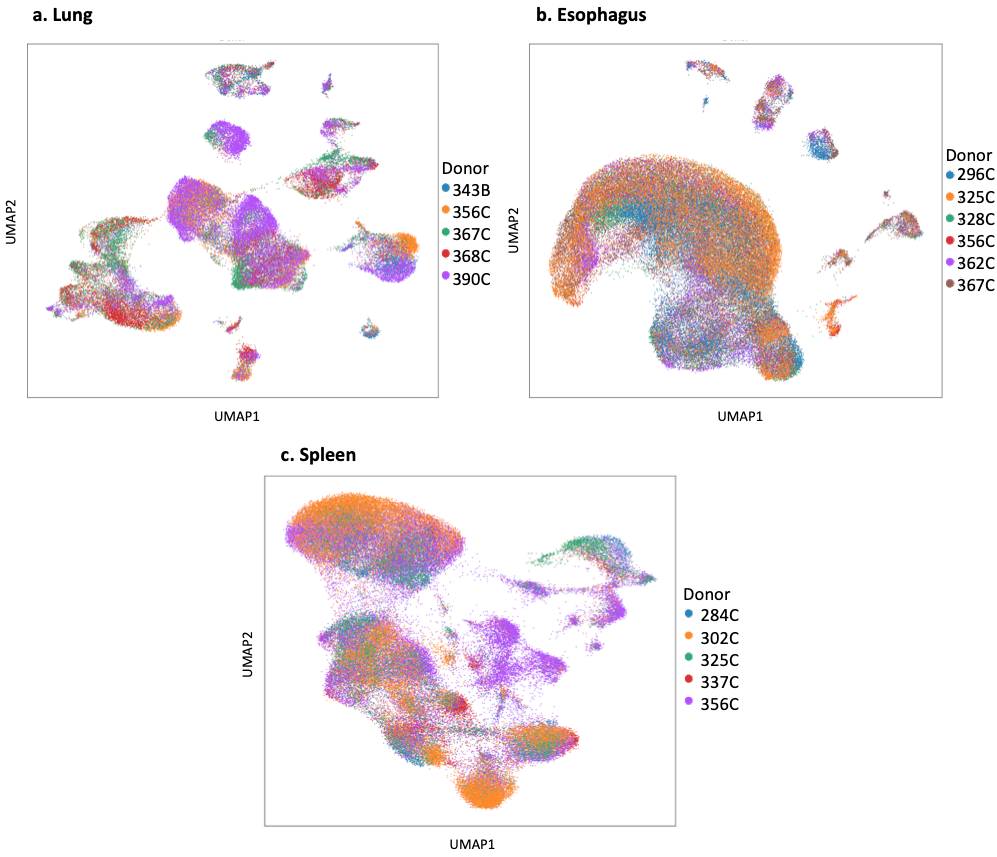


#### **Fig S10: Distribution of cells from different donors.**

UMAP plots for all three organs coloured by donor, for lung (a), esophagus (b) and spleen (c).

SCGB1A1 BPIFB1
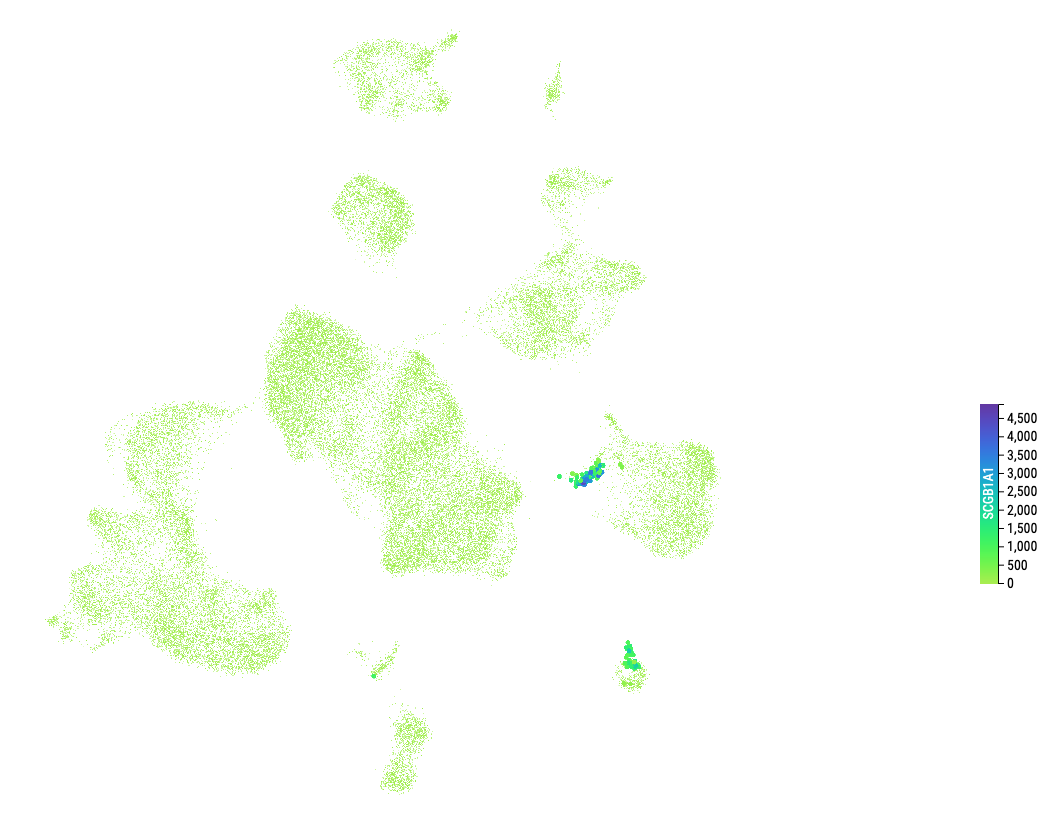

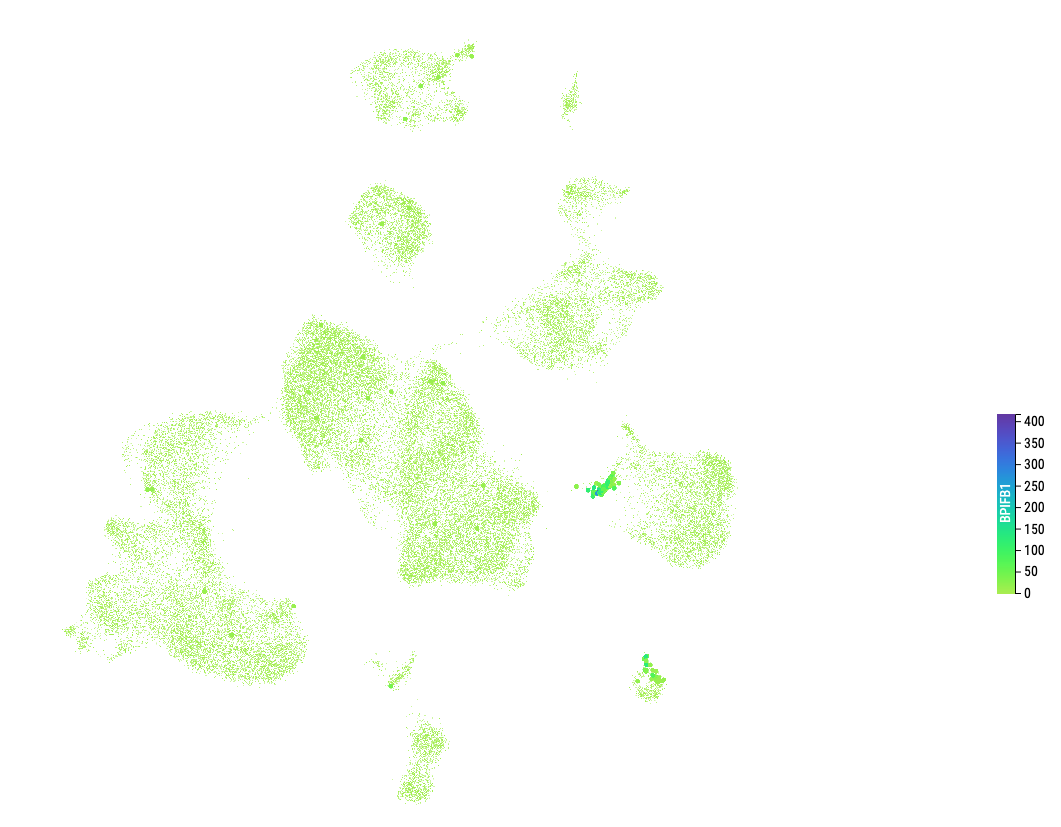


MSMB SCGB3A2
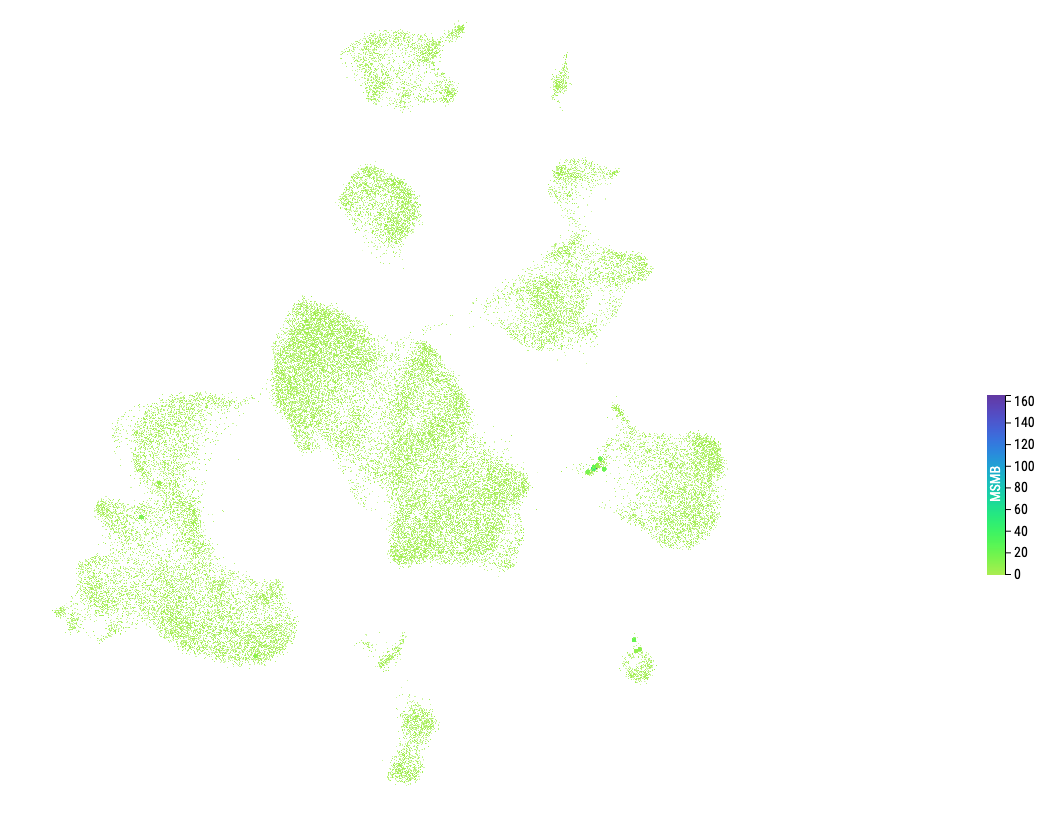

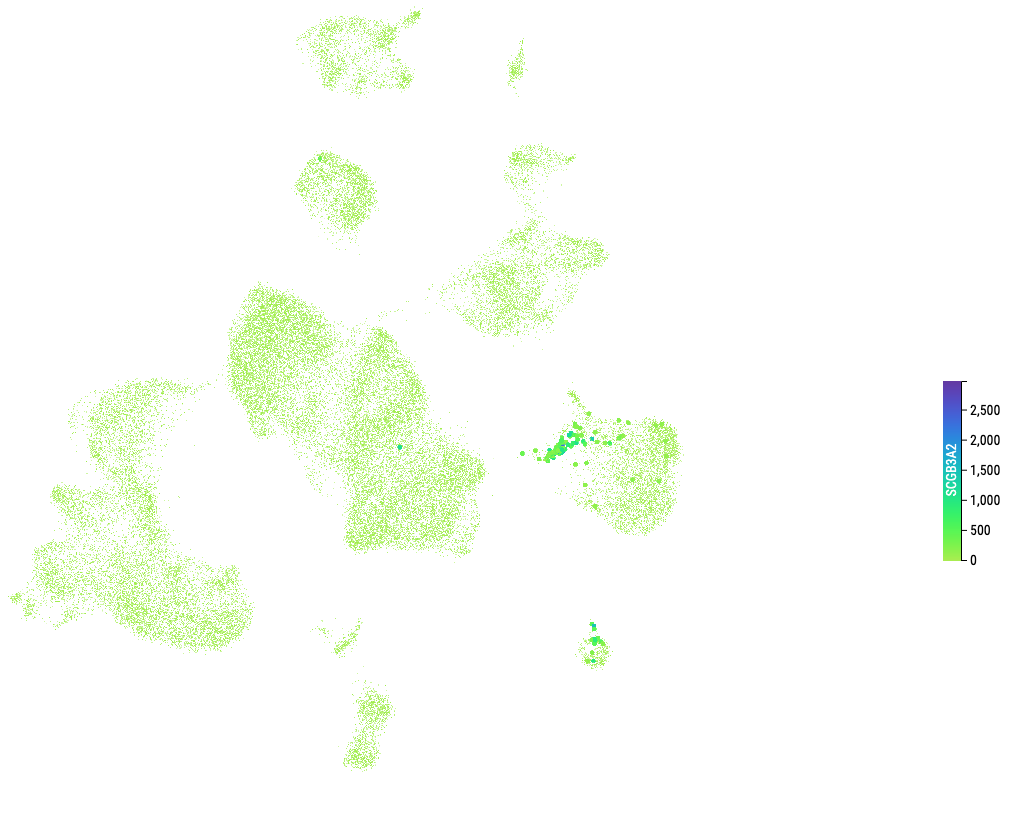
 BPIFA1 SCGB3A1


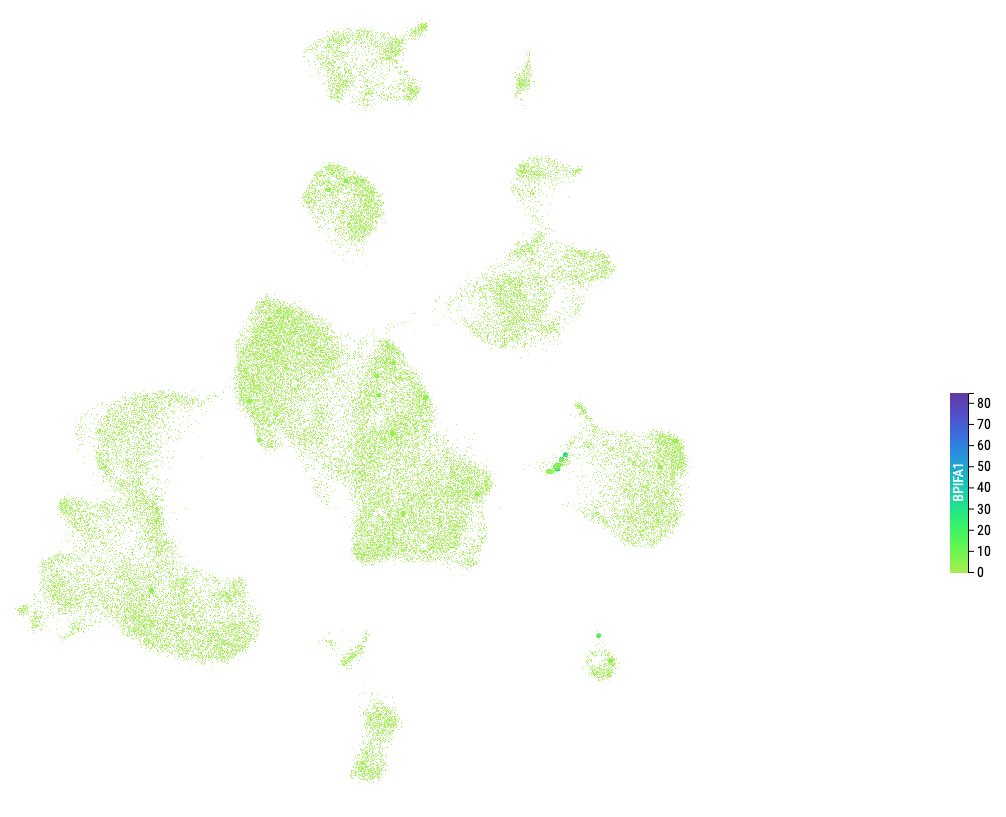

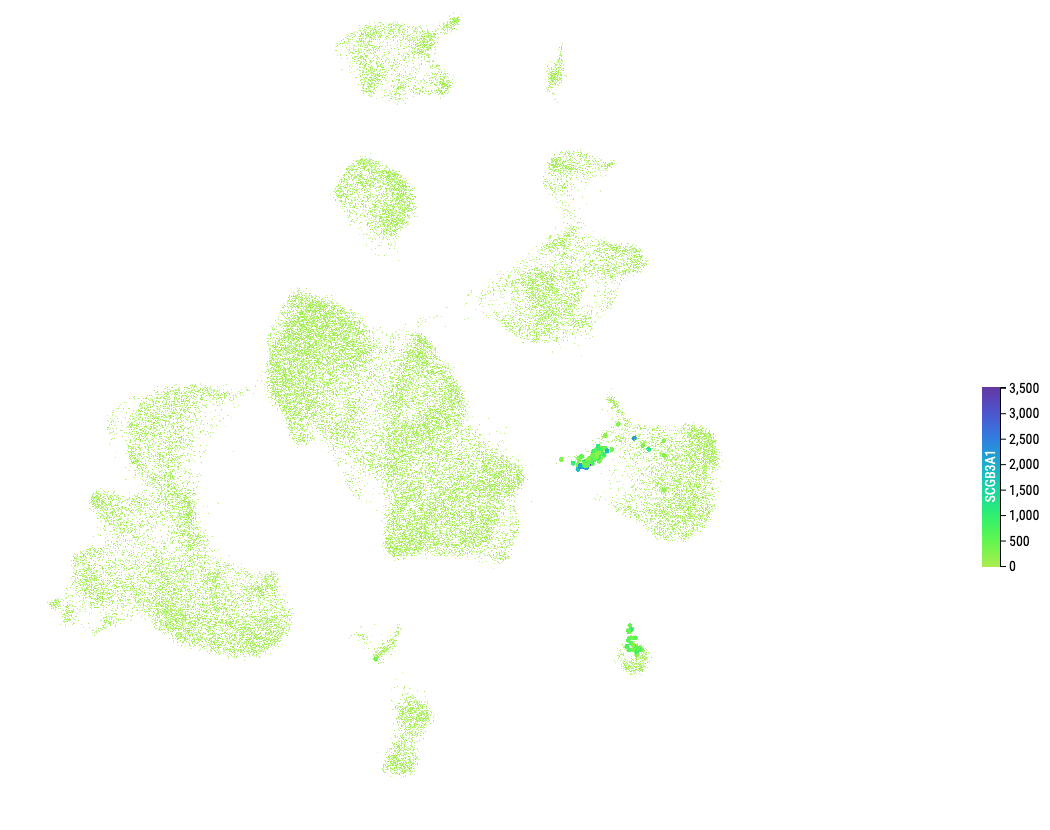


#### Fig S11. Expression of Club cell markers in lung.

The raw read counts of Club cell marker genes are shown on the lung UMAP plot as viewed in <https://www.tissuestabilitycellatlas.org/lung>. Cells with no expression are indicated by smaller dots. Those with any expression by a larger dot with color indicating expression level.


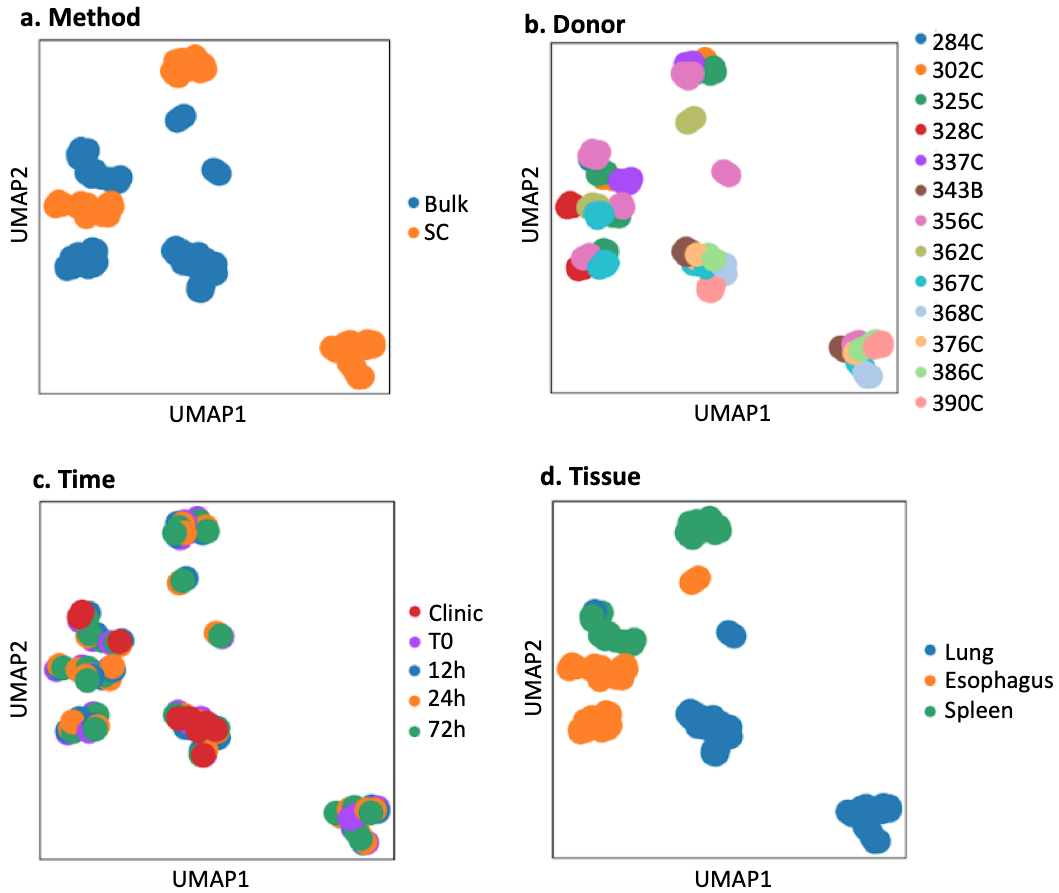


#### **Fig S12**. **Bulk RNA-sequencing data comparison with single-cell RNA-sequencing.**

The single-cell data was combined as pseudo-bulk per sample, PCA was performed for highly variable genes across samples, UMAP coordinates were calculated based on PCA. The clusters of samples are shown on UMAP plot, coloured by method (a), donor (b), time (c) and tissue (d).

a.Donors

Lung Esophagus Spleen


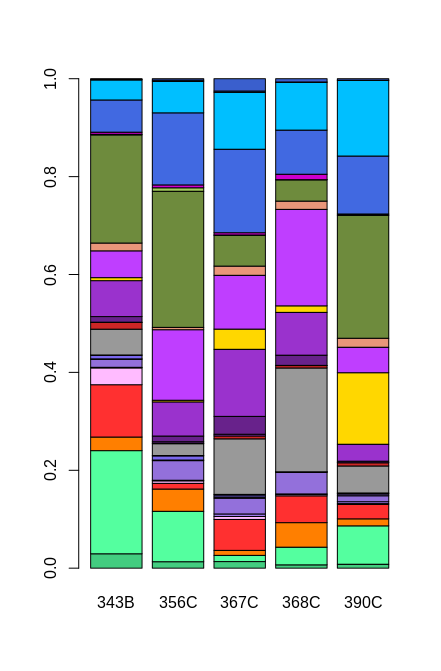

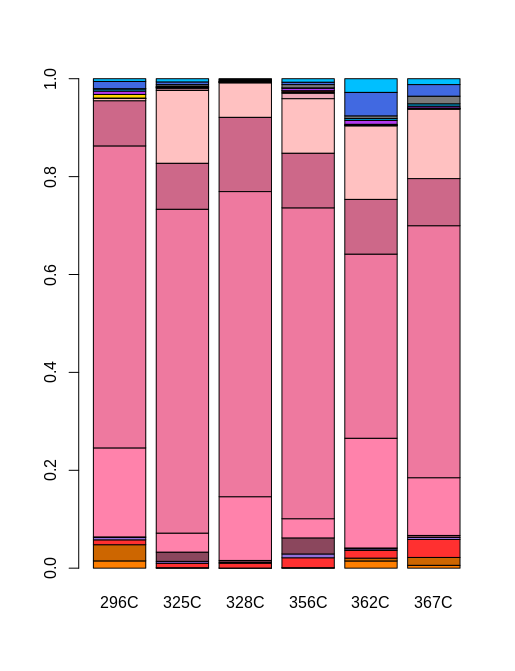

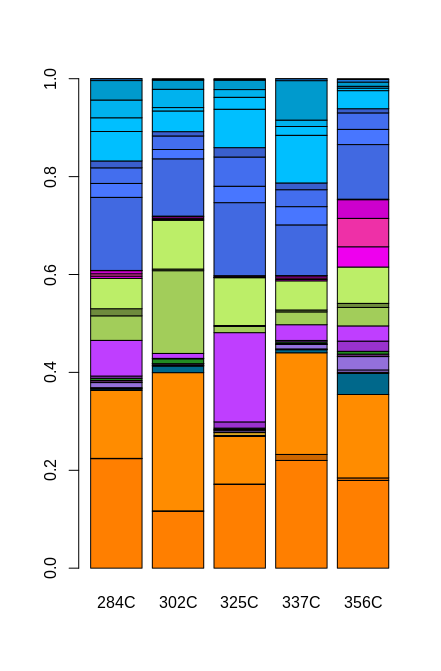


b.Time

Lung Esophagus Spleen


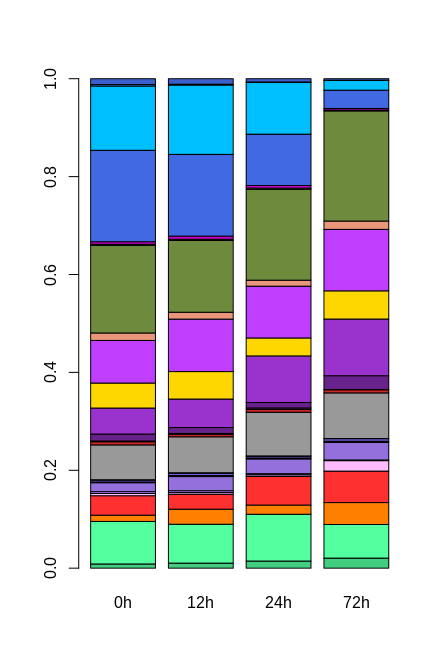

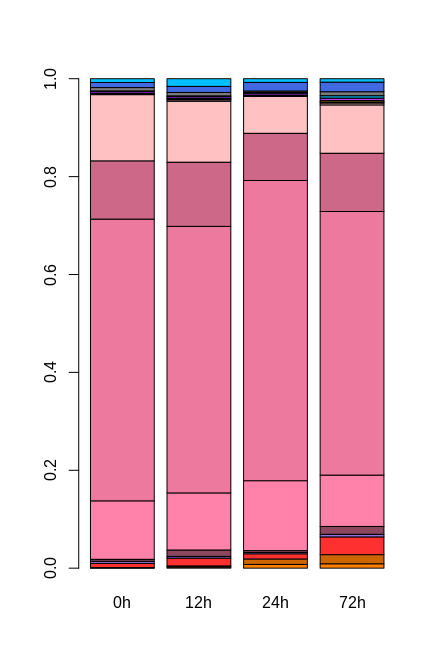

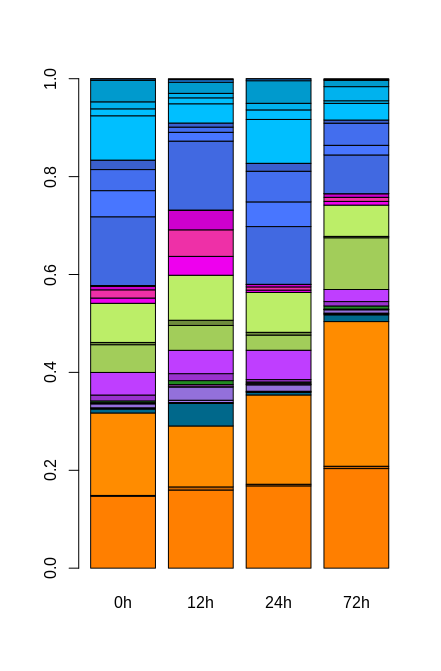


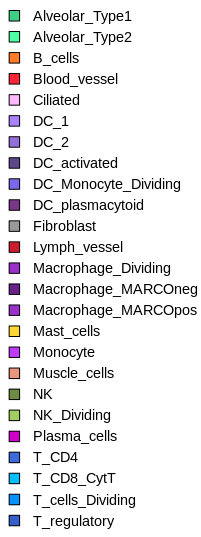

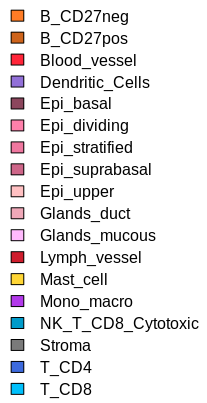

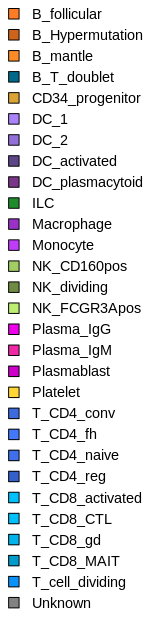


#### **Fig S13: Proportions of cells types shown per donors or time.**

Proportions of cell types in each donor (a) and time point (b) are shown for lung, esophagus and spleen.

1. Lung


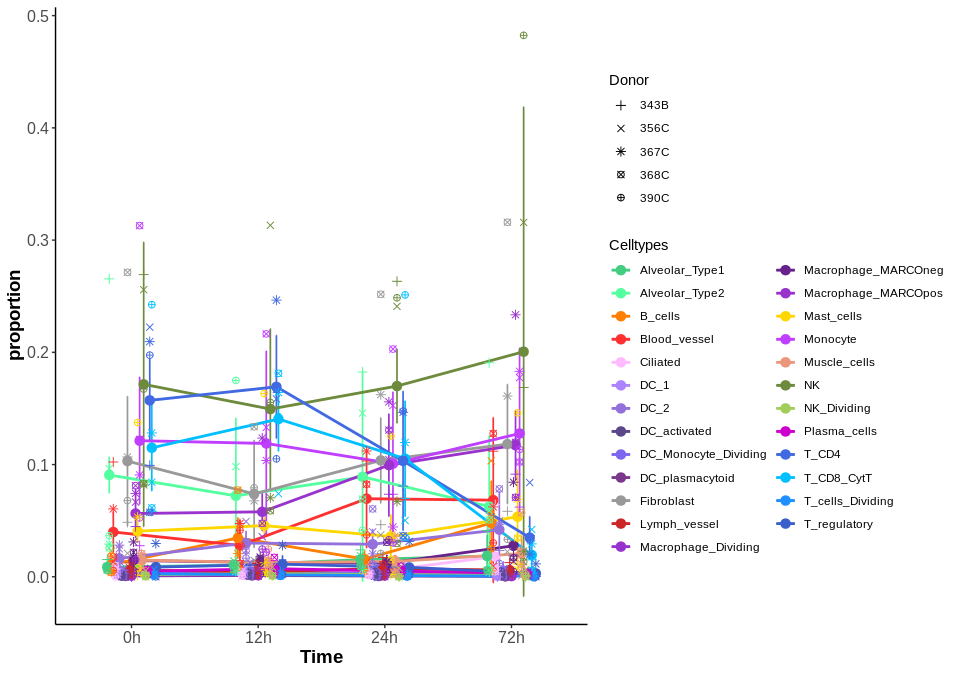


1. Esophagus


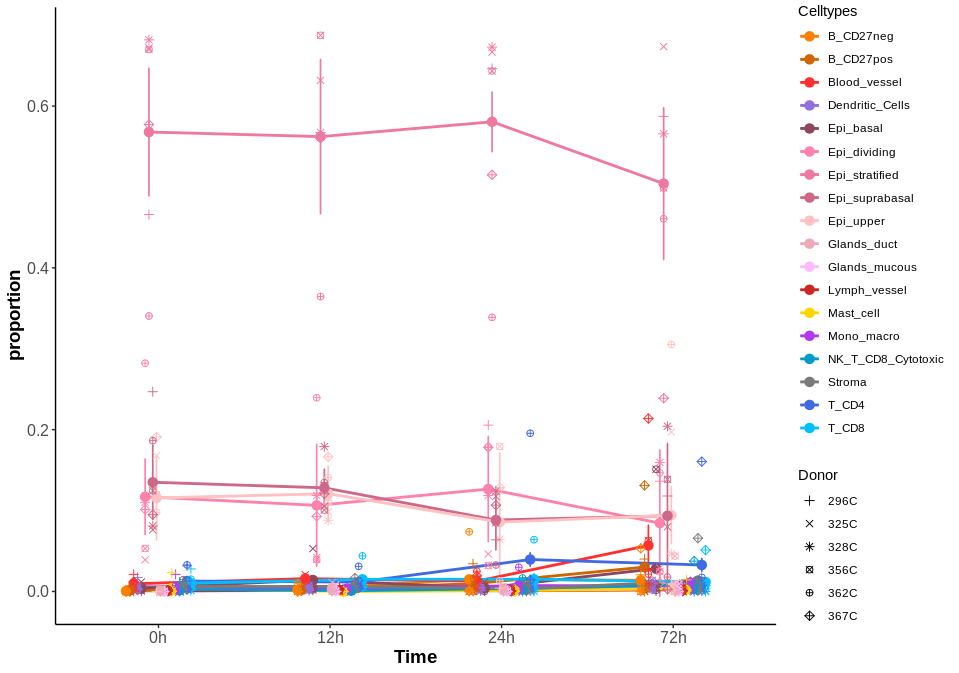


1. Spleen


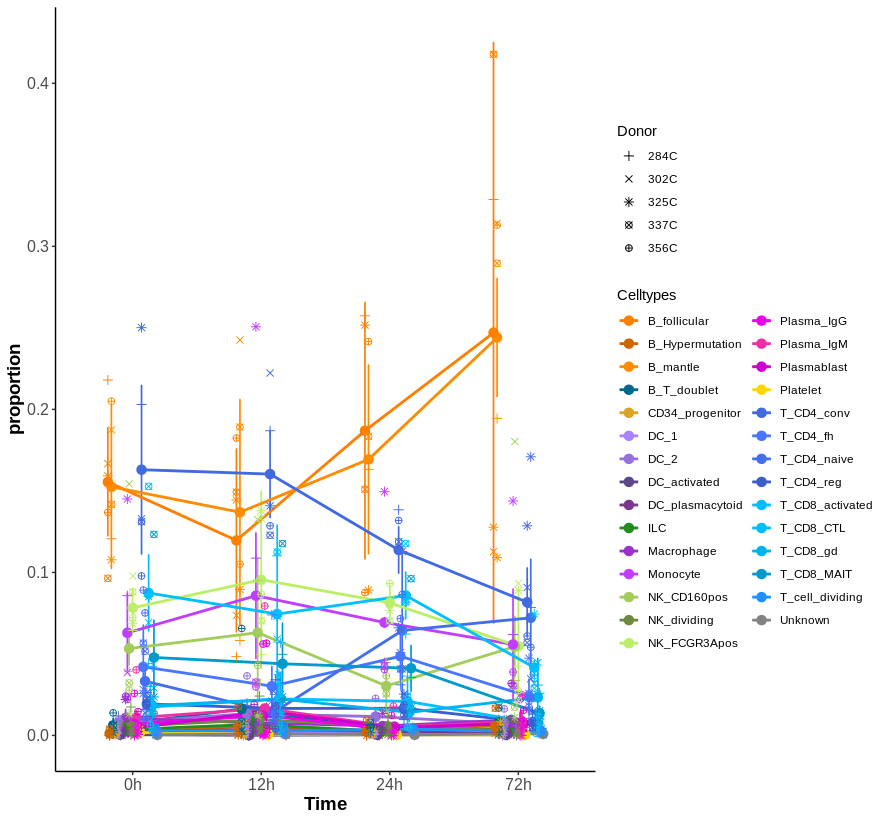


#### **Fig S14: Change in the proportion of cell types in time.**

Proportions of cells in each sample are shown for lung (a), esophagus (b) and spleen (c). Cell types and donors are indicated by color and shape, respectively. Means between donors for every cell type at given time points are shown by dots, connected by a line corresponding to the cell type. Error bars indicate standard deviation.

#### **
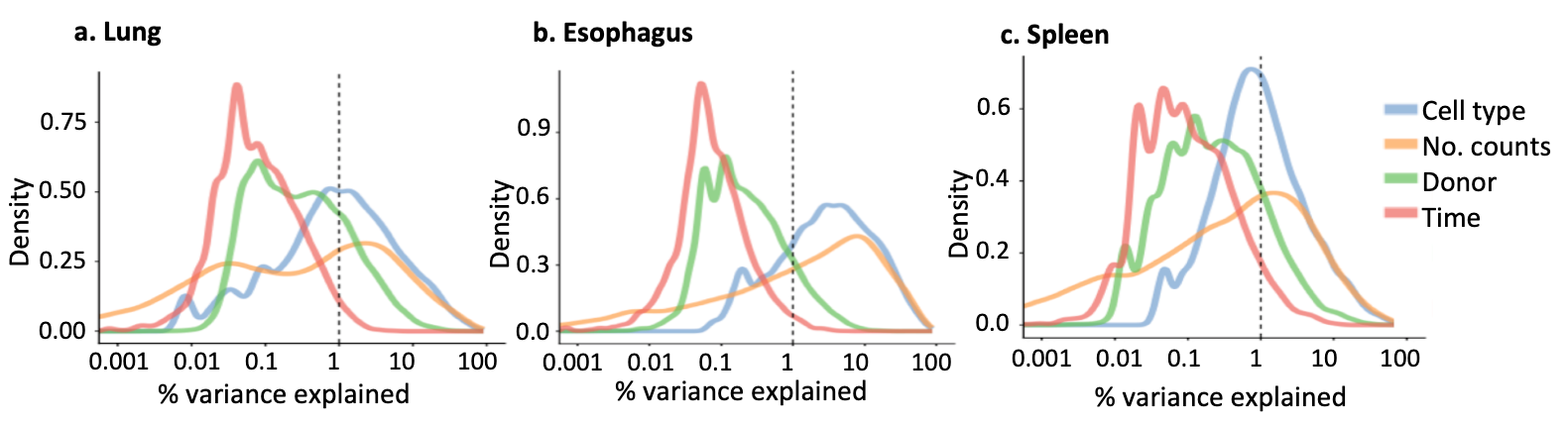
**

#### **Fig S15**. **Time explains the least of variance in gene expression.**

Percentage of variance in gene expression explained by cell type, number of counts, donor and time in lung (**a**), esophagus (**b**) and spleen (**c**). Gene-wise density plots of the gene-wise marginal R^2^ for each variable is shown.


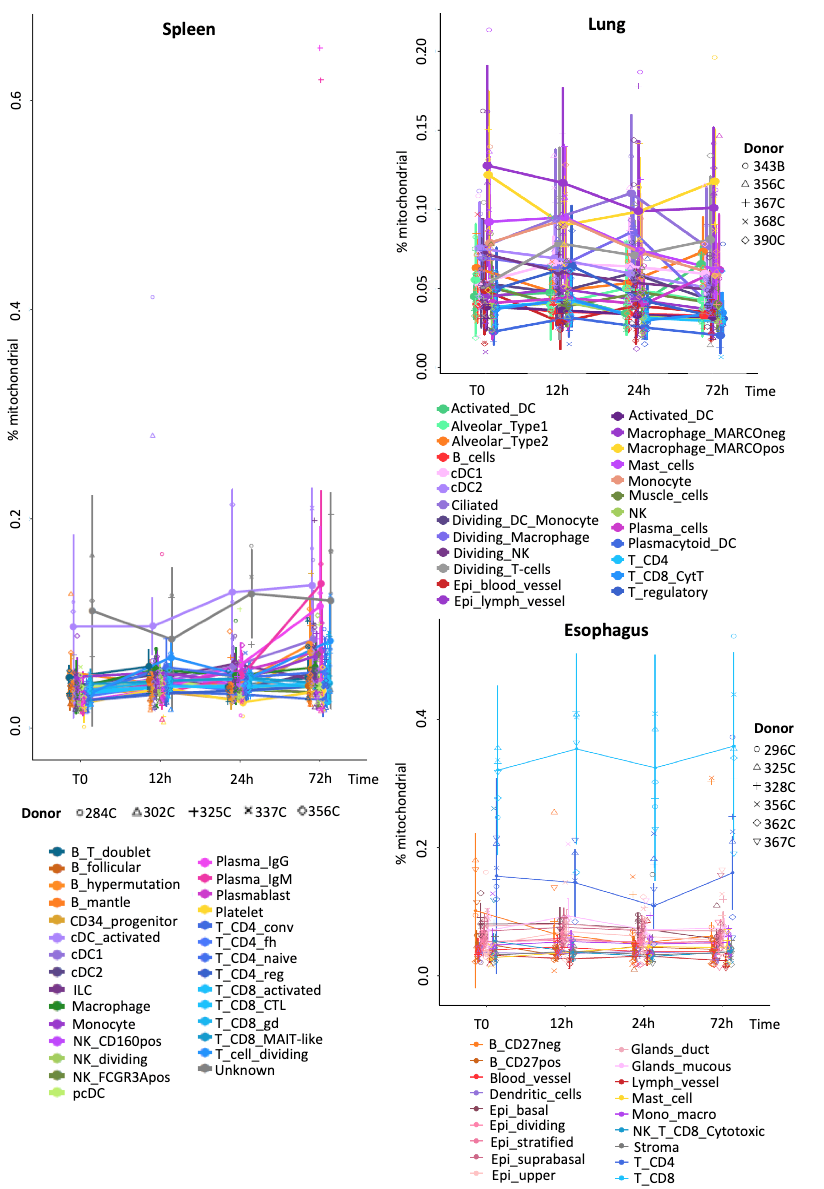


#### **Fig S16. Mitochondrial percentage differs between cell types.**

Mean mitochondrial percentage and standard deviation across cells and donors was calculated per every cell type in every time point. Dots represent the mean mitochondrial percentage across donors for a cell type and time point. Whiskers show standard deviation. The same cell types are connected by line. Mean mitochondrial percentages are shown separately for every donor, cell type and time point by shapes corresponding to donors. All colors represent cell types.


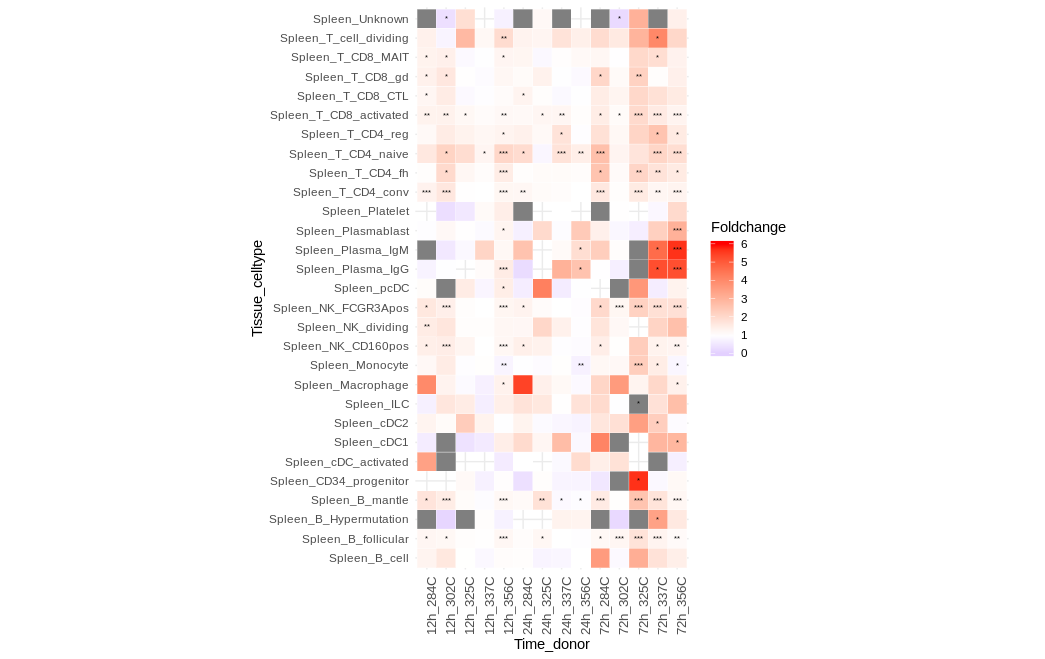


#### **Fig S17**. **Change in the percentage of mitochondrial reads with time and by cell type and donor in spleen.**

The fold change (FC) of mitochondrial read percentage is shown for every cell type between later timepoints (12h, 24h and 72h) and time T0. FC is indicated by color with white indicating no fold change (FC=1), blue indicates a drop in mitochondrial reads compared to T0 (FC<1), red indicates increase in mitochondrial percentage compared to T0 (FC>1). Benjamini and Hochberg adjusted p-values are indicated by asterisk as follows: p-val<0.01*, p-val < 0.00001** and p-val < 0.00000001***. All cells are used, including those with high mitochondrial percentage (>10%) whose cell type annotations were derived via scmap tool based on similarity to the annotations to cells with lower mitochondrial percentage. Time points with fewer than 5 cells per time point are shown in grey. Missing values (no cells in either comparison) are shown by light grey crosses.


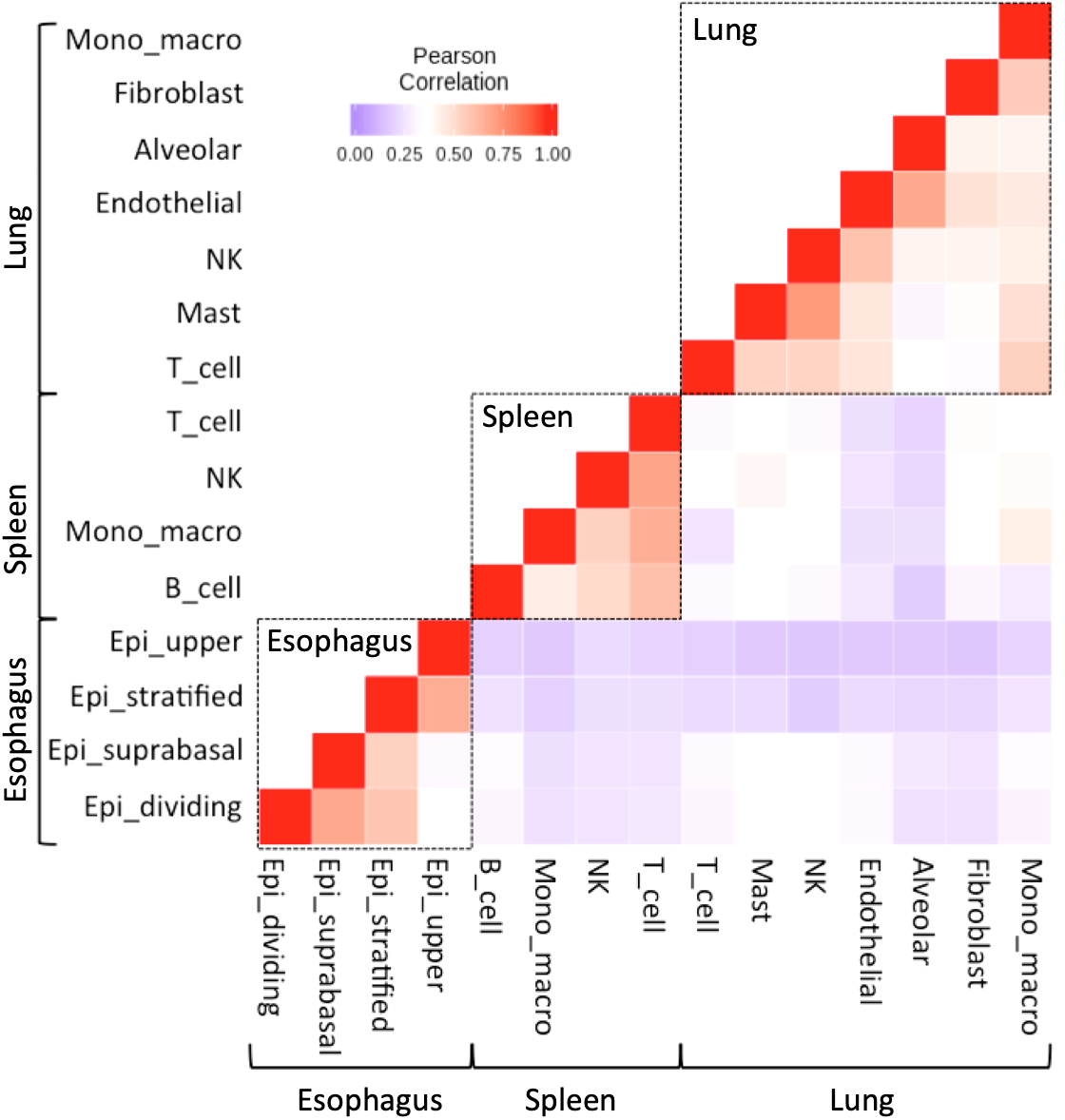


#### **Fig S18**. **Gene signatures associated with storage time are correlated with tissue type and not cell type.**

For the major cell types for each tissue (see x axis), we calculated the explanatory variance in gene expression over time for all genes. Using this matrix we then examined the correlation between cell types and these are plotted as Pearson correlation coefficients, indicated by color.

a Lung


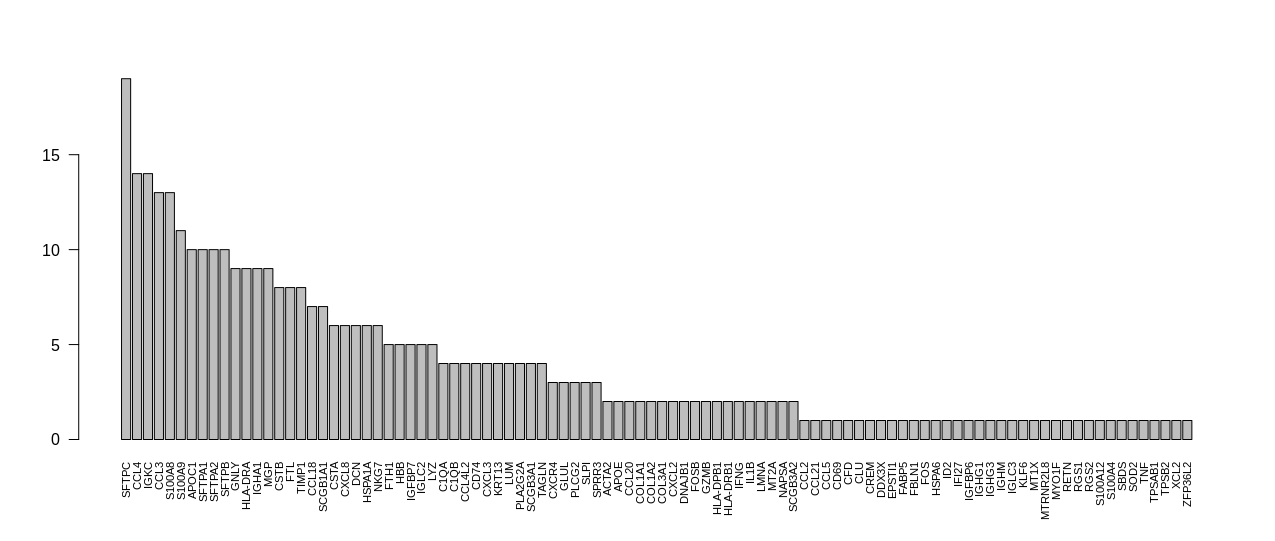


b

Esophagus


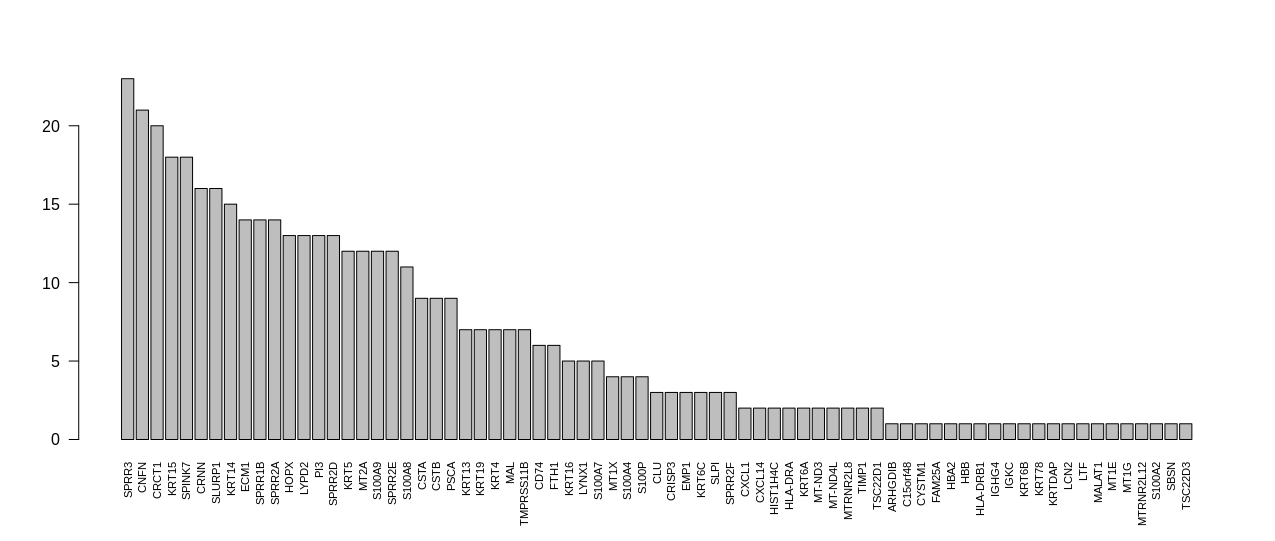


c Spleen


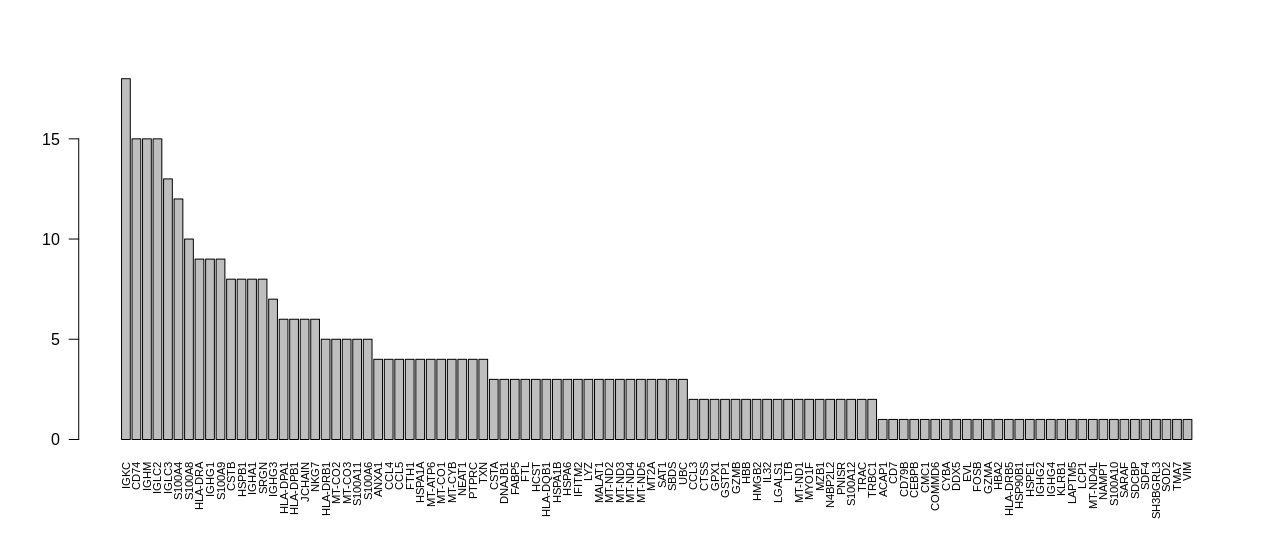


#### **Fig S19**. **Frequency plots of top ambient RNA contamination genes.**

Frequency of top 20 contaminating genes from any of the samples in lung (a), esophagus (b) and spleen (c) tissues. SoupX algorithm infers genes for a 10x run that are likely to contaminate the samples. The top 20 of these genes per sample were used to calculate the frequency across samples per tissue.
