# Supplementary material for: scRNA-seq assessment of the human lung, spleen, and esophagus tissue stability after cold preservation
Source: Genome Biol. 2019 Dec 31;21:1. doi: 10.1186/s13059-019-1906-x (PMC6937944; doi:10.1186/s13059-019-1906-x)
Supplement: Supplementary file 9 — Additional file 9: Review History. [file 13059_2019_1906_MOESM9_ESM.docx]

Review History

**First round of review**

**Reviewer 1**

**Are you able to assess all statistics in the manuscript, including the appropriateness of statistical tests used?**

Yes, and I have assessed the statistics in my report.

**Comments to author:**

This is an essential work. While conceptually it is a simple extension of the previous study of Wang et al showing that mouse kidney gene expression patterns are remarkably stable when the organ is stored on ice, it nevertheless extends this result to human samples and three organs. This is critically important for the Human Cell Atlas (HCA) Project, where samples are often shipped on ice for analysis.

The work was executed with care. For example, whole genome sequencing was carried out for all 12 organ donors contributing samples for this study.  The total number of cells analyzed, 240,000, is quite impressive. Overall, one is impressed with the thoroughness of the study.

This paper references van den Brink Sc et al (2017)(ref 20) for 37 deg proteolytic cell dissociation induced gene expression changes. This reviewer would add the slightly earlier Adam M, Potter AS and Potter SS Development paper also showing this and further demonstrating that these artifacts can be reduced through the used of psychrophilic cold active proteases.

One concern is that the expected gene expression changes associated with cold storage stress might well overlap with the stress related cell dissociation induced gene expression artifacts. So, could the dissociation procedure used, which at least in some cases involved 37 deg proteolytic digestion for over an hour, hide gene expression changes associated with cold storage, in some cases for three days? It would have been preferable to either use cold cell dissociation protocols to reduce the dissociation gene expression artifacts, or to use nuclei, which can be prepared on ice. Nevertheless, it would not be reasonable to request a complete re-do.

This study used samples that were quickly perfused with cold University of Wisconsin (UW) solution after cessation of circulation and then transferred to cold Hypothermasol FRS preservation solution. Such rapid perfusion with UW solution is not likely to be possible for all samples. It would have been useful to address this perfusion as a variable. How important is it?

The topic of cell death is discussed at the bottom of page nine, starting at line 212. This would seem an important issue, as one might expect that with prolonged storage more cells would die. It is not clear that the best way to address this is through scRNA-seq. Dead cells might be expected to be preferentially lost during the dissociation/cell purification process. Why not just do TUNNEL, cleaved caspase analyses on tissue sections at different storage times?


Minor points.

There is some mixing of fonts and spacing that is annoying.

Line 283. The Bonferroni FDR correction is extremely stringent. Then it jumps to no correction. It might be useful to consider intermediate stringencies, like Benjamini and Hochberg.

Whole genome sequencing was carried out for all donors, but it is simply stated that "…none of the study participants displayed gross genomic abnormalities". What does that mean? There is no description of the level of analysis carried out. No major CNVs? Was anything else looked for?

It is a little disappointing that all 94,257 cells from the spleen were immune cells, apparently the result of no enzymatic digestion.

**Reviewer 2**

**Are you able to assess all statistics in the manuscript, including the appropriateness of statistical tests used?**

No, I do not feel adequately qualified to assess the statistics.

**Comments to author:**

Please consider the following questions:

- Are the conclusions adequately supported by the data shown? If not, please explain

This paper describes a method for cold storage of primary human tissue samples that requires no processing at the clinical site beyond sample collection, thus allowing a 24h window (at least) for shipping, tissue dissociation and scRNA-seq. The paper provides useful information for investigators involved in similar projects. Its major input is to show that hypothermosol cryopreservation is well adapted to store tissues before dissociation for single cell studies. While this point has already been shown for kidney, it is further extended here for lung, thymus and esophagus, at least at some levels. The authors claim that lung and esophagus were stable for 72h for all of the metrics they tested, and that some changes were observed in spleen at this time, but not before. They claim that this approach can be useful for future HCA study design. A restriction to their conclusions is that lung dissociation mostly released immune cells, but nearly no other cell types. For me, it is important to test additional protocols of dissociation in order to ensure a better dissociation of lung parenchyma, thus establishing on a more comprehensive number of cell types the benefits of cryopreservation.
The study results in a very large single cell data set for spleen, esophagus, and lung immune cell, with over 240,000 single cells with detailed cell type annotations, which has been collected after different times of storage at low temperature, in order to assess RNA stability at a single cell level. This analysis is coming with whole genome information, allowing future eQTL analysis. All data have been submitted to the open access Human Cell Atlas data coordination platform, making freely available this resource.
It is also indicated that the annotated data will be available at www.tissuestability.org, but this link was not accessible when I tested it.

- Are the methods appropriate to the aims of the study, are they well described, and are necessary controls included? If not, please specify what is required.

This is a well written manuscript, and a carefully designed study.
An interesting aspect is a tentative quantitative assessment of the different experimental parameters affecting variance. Providing such values would be extremely valuable. Figure 4j and Supplementary Figure 10 suggest that time of storage has less impact on gene expression than cell types, number of counts or donor. Intriguingly, cell type explains no more than 10% of the variance, and overall, it seems that a large part of the variability is unexplained. This has to be clarified. In my opinion, each cell-type has probably its specific behavior. From the shown graphs, it seems that the total percentage of explained variance is probably below 20%. That implies that 80% of it comes from additional variables. A clarification of this point is key, because it will rate more clearly the impact of time of storage.
As such, the paper contains a wealth of information, but clearly requires some important clarifications. To demonstrate the usefulness of cold conservation, additional results should be provided, regarding the morphology of the different cell types, the percentage of live cells, etc. This would complement well paragraphs about diversity of cell types, tissue processing, and annotation of cell types.
The central hypothesis is that cold storage in an appropriate buffer will slow down RNA degradation. The observation of stable RIN numbers around 7 already provide an indication about the real stability of the samples, although it does not exclude cell-specific effects. These correct values probably explain the acceptable quality of the reads that is shown in Figure 1 and supplementary figure 1. Long term storage however resulted in spleen in an increased % of mitochondrial sequences, and of intronic reads, raising the interesting observation that nuclear mRNA is less susceptible to enzymatic degradation (a good point for using single cell NucSeq protocols).
The authors refer to "ambient RNA" for droplets that contain 1-2 UMIs, "debris" for droplets that contain 3-100 UMIs and "cellular material" for droplets that contain more than 100 UMIs. They notice that the mean number of UMI increased in debris and decreased in cellular droplets by 72h (but not 24h) in spleen (Figure 3.c, d), but not in the two other tissues. While this is understandable, a direct comparison with a cell viability assay would be very valuable.

- Are sufficient details provided to allow replication and comparison with related analyses that may have been performed? If not, please specify what is required.

More details about the protocol of cold conservation should be provided regarding sampling areas (samples are 0.5 to 2 cm3), ratio between volumes of submersion and tissue, etc. I suspect that these parameters can be important and can affect the results. Hence, protocols should be more carefully documented. As a non-limitative list of issues : can there be an effect of the surface of exposition to the solution ? What are the evidences that the solution really penetrates into the core of each sample ? What are the differences between tissues?
A key parameter is clearly the dissociation, and this dissociation is quite tissue-dependent. Hence, collagenase dissociation of esophagus released a large fraction of epithelial cells (89% of all cells), which were virtually absent from the lung (10% of all cells) and spleen (0%). Formally, this clearly demonstrates that the same dissociation protocol will not be applicable for any cell type. For instance, any conclusion about non-immune lung cells have probably to be considered with some caution, as it is clear that this preservation method is not adapted to study lung epithelial cells. After comparing bulk and Sc-bulk, the authors conclude that differences are due to the dissociation protocol rather than to conservation. Surprisingly, while dissociation in lung is supposed to extract mostly immune cells, and few epithelial cells, they report p-values and log2ratio that do not necessarily fit with this idea !  For instance, FOXJ1 and TPPP3, 2 specific markers of ciliated cells, are not differentially expressed between bulk and pseudo-bulk! The same situation for MUC5B and MUC5AC, two markers of mucous cells, KRT5, a marker of basal cells. Surprisingly, SCGB1A1, a marker of CLUB cells is enriched in pseudo-bulk!

- Does the work represent a significant advance over previously published studies?
- Is the paper of broad interest to others in the field, or of outstanding interest to a broad audience of biologists?

In conclusion, this work contains interesting information regarding processing of human samples, but complementary approaches are needed to provide a definitive demonstration of its applicability.

Minor issues :
Citation of supp table 5 is wrong. This is supp table 4.
Supp table 5 is not quoted
Supplementary Figure 1 : scale bar is lacking for lung and spleen
Legends of Figure 5 and Supplementary Figure 12: "Benjamini and Hochberg", not "Benjamin and Hochberg"

**Reviewer 3**

**Are you able to assess all statistics in the manuscript, including the appropriateness of statistical tests used?**

Yes, and I have assessed the statistics in my report.

**Comments to author:**

This is an important and well executed study of the relationship between ischaemia time and cold preservation methods for three tissue types. The authors show convincingly that with cold preservation storage for these tissue types, upto 24 hours cold storage does not contribute significantly to the variation in single cell gene expression. A greater proportion of variance is explained by donor/individual factors, and probably also dissociation method, than length of cold storage for reasonable periods. Spleen and pancreas(not analysed here) are notoriously among the earliest organs to autolyze after ischaemia, it is interesting to see that a fair degree of preservation can be achieved by cold temperature storage before processing. This doesn't of course mitigate host/donor factors which contribute greater variance, such as the degree of organ preservation before sampling. I don't have major technical or conceptual criticisms but some suggestions for improvement.

Suggestions for improvement:

-     It seems the correlations with tissue necrosis (an individual donor factor which could have a major influence on expression variance) and cell death were established by trypan blue and/or histology of the organs, or by scRNA-seq alone. It would be of value to the dataset to either perform some IHC on the frozen sections/FFPE from the sampled organ regions to document the basic metrics commented on (eg viability, proportion of replicating cells, proportion of the major cell types determined by antibody markers) and the major cell type proportions present. This could also be done by FACS if there is remaining tissue available.

-     The conclusions/discussion could do a better job of synthesizing both the key findings and commenting on the limitations - the main one being that the number of individuals sampled and tissue types will potentially limit some of the conclusions - the authors do mention this along the way, but it would be easier for the reader to have the key synthesis in the discussion/conclusions. The need to evaluate/minimize/control for the consequences of ischaemia and dissociation method (less directly examined in this study) in each tissue type being studied by scRNA-seq is a key finding that should be highlighted in my view.

**Reviewer 4**

**Are you able to assess all statistics in the manuscript, including the appropriateness of statistical tests used?**

Yes, and I have assessed the statistics in my report.

**Comments to author:**

The here presented work by Madissoon and colleagues interrogates the effect of sampling time on single-cell gene expression profiles. In contrary to previous work by the GTEx consortium, they sample in the context of organ transplantation allowing rapid tissue procurement and minimizing PMI. As for transplanted organs, the study maintains the sample at cold temperatures and determines alterations over a period of 72 hours. This work is of crucial importance to guide sampling standards for tissue and organ atlas project that require intact, viable and unaltered cells. The work concludes that samples can be stored for 72 hours without the introduction of major gene expression changes or cell composition biases. The conclusions are supported by sampling from 3 different organs, 5 donor, 4 time points and thousands of cells. The study is appropriately scaled, timely, and of high importance for the single-cell community. However, the structure of the manuscript can be improved to focus on the main messages of the paper and additional analysis need to better support the conclusions.

1.     The studies includes many arguments that directly relate to the design of HCA projects. However, the work will be of interest for many other projects in the single-cell community. Thus, the conclusions could be formulated more general.
2.     Since degradation starts from the 5' transcript ends, it would be of interest to see if profiles obtained using the 10x Genomics 5' assays would be affected. Many future (autoimmune) disease-focused projects will perform 5' profiling combined with TCR genotyping. Thus, a proof-of-concept is important in this context and can be done on a single sample and tissue type.
3.      The Fig. 2 should be re-ordered to have the summary first and subsequently the spleen-specific analyses.
4.     The definition of empty drops and debris is challenging and thresholds need to be adjusted per sample. As the distribution is sequencing depth dependent a fixed cutoff could result in cells assigned to debris in lowly sequenced datasets. Can this threshold be defined automatically per sample, e.g. through the automatic detection of the inflection points (with EmptyDrops; Lun et al., 2018).
5.     The mean number of UMIs in debris increased at 72h. What about the total UMI numbers in empty drops? More informative would be to show the number of reads in debris and empty drops as this directly relates to the "wasted" sequencing budget. Also, the figure does not fit the text (e.g. no Figure 3d).
6.     The results of the viability analysis might be a good fit as introduction of the cell composition results. At the moment they seem to be a bit disconnected from the results, also because lung tissue is mentioned as affected.  
7.     As the focus of the work is on the preservation test, the description of the tissue composition might be reduced. Important is the cell type proportion between time points and donors and not the detailed annotation.
8.     The composition analysis and the cell type signatures should come directly after the presentation of tissue heterogeneity and annotation. Like this the work keeps the focus on the changes induced by timing. All extra analysis can be shifted to the end.
9.     The mapping of cell types between tissues in certainly of interest, but seems to be a bit off-topic in this work. These valuable datasets will allow more specific analyses that go beyond this work and the authors better focus on the key messages of this study related to the conservation of gene expression profiles and tissue composition.
10.     The conservation of cell type composition is a key message. Can the authors add more evidence (statistical tests) to support their conclusions that a) cell type composition is conserved over time points and b) the cell type variability is higher between donors than time points.
11.     This is personal taste, but the details of the analyses (e.g. use of packages and software) can be moved to the Methods.
12.     The title could be re-phrased as tissue stability can refer to many different parameters.
13.     It is mentioned twice in the beginning that the samples are also snap frozen for bulk RNAseq.

**Authors Response**

**Point-by-point responses to the reviewers’ comments:**

Comment

*Response*

Reviewer 1

1.1

This paper references van den Brink Sc et al (2017) (ref 20) for 37 deg proteolytic cell dissociation induced gene expression changes. This reviewer would add the slightly earlier Adam M, Potter AS and Potter SS Development paper also showing this and further demonstrating that these artifacts can be reduced through the used of psychrophilic cold active proteases.

*Thank you for pointing out this manuscript, we have now added this reference (ref 21).*

1.2

One concern is that the expected gene expression changes associated with cold storage stress might well overlap with the stress related cell dissociation induced gene expression artifacts. So, could the dissociation procedure used, which at least in some cases involved 37 deg proteolytic digestion for over an hour, hide gene expression changes associated with cold storage, in some cases for three days? It would have been preferable to either use cold cell dissociation protocols to reduce the dissociation gene expression artifacts, or to use nuclei, which can be prepared on ice. Nevertheless, it would not be reasonable to request a complete re-do.

*The reviewer raises an important issue. Yes, we agree that there might be an overlap between the cold storage and cell dissociation induced stress genes. We note, that our experimental design included one organ (spleen) which was mechanically dissociated, avoiding enzyme induced changes. Overall, we expect that even if storage and dissociation signatures overlap, storage related changes should still appear in the analysis, since all the time-points had the same dissociation treatment. We tested for over-representation of the time-related genes in the dissociation-related gene set, and indeed found a higher than expected overlap between the two sets (p-val <0.01 in all three tissues). This demonstrates that our approach is able to detect both the cell type specific genes (Supplementary Figure 13) as well as the genes affected by dissociation. This information together with appropriate statistical tests and conclusions is now added to the manuscript.*

1.3

 This study used samples that were quickly perfused with cold University of Wisconsin (UW) solution after cessation of circulation and then transferred to cold Hypothermasol FRS preservation solution. Such rapid perfusion with UW solution is not likely to be possible for all samples. It would have been useful to address this perfusion as a variable. How important is it?

*The reviewer raises a valid point. As the samples obtained for this study were all from organ transplant donors, rapid perfusion with organ preservation solution is part of the clinical protocol and we cannot change this. This perfusion is required to occur within 12 minutes of asystole (added in to methods). Warm ischaemic time can certainly impact on gene expression changes, as demonstrated by Ferreira et al (ref 23 in our manuscript, discussed on page 6), but we are unable to assess this here.*

*Of note, a protocol of rapid cooling and cold preservation is also relevant for biopsies from live donors, making our findings relevant for different study designs.*

1.4

The topic of cell death is discussed at the bottom of page nine, starting at line 212. This would seem an important issue, as one might expect that with prolonged storage more cells would die. It is not clear that the best way to address this is through scRNA-seq. Dead cells might be expected to be preferentially lost during the dissociation/cell purification process. Why not just do TUNNEL, cleaved caspase analyses on tissue sections at different storage times?

*Thank you for this suggestion. We have now performed TUNEL staining on 0h and 72h tissue sections from all three tissues (see new Supplementary Figure 8). TUNEL staining intensity varied both between and within individual samples, with staining being noticeably patchy. There was a trend of higher staining at 72 hours for all three tissues, but t0 staining in spleen was higher than in the other two tissues. Overall these findings are consistent with increased cell death at later time points, and with a larger effect of cell death observed in spleen. Since dead cells should be removed in the washing steps and viability columns, we expect not to observe the cells at the late stages of apoptosis in our sequencing data. However, we do observe more debris in spleen by 72h that can indicate increased sensitivity to dissociation after prolonged storage.*

*We have added text and discussions about the viability to the manuscript.*

1.5

There is some mixing of fonts and spacing that is annoying.

*We have now carefully read the manuscript and hopefully formatted evenly across the text.*

1.6

Line 283. The Bonferroni FDR correction is extremely stringent. Then it jumps to no correction. It might be useful to consider intermediate stringencies, like Benjamini and Hochberg.

*Thank you for the suggestion. We have changed the Bonferroni FDR correction to Benjamini and Hochberg throughout the study, and updated the tables and text accordingly.*

1.7

Whole genome sequencing was carried out for all donors, but it is simply stated that "…none of the study participants displayed gross genomic abnormalities". What does that mean? There is no description of the level of analysis carried out. No major CNVs? Was anything else looked for?

*We have now added analysis methods and Supplementary Figure 1 regarding WGS data. We observe that no donor carries CNVs larger than 2Mb and see at most 2 CNVs>500kb per donor. The total affected size <30Mb (1% of the genome) which is comparable to the other publicly available WGS data. Furthermore, 29% of CNVs (44% of affected bases) are listed as common CNVs in the Database of Genomic Variance (DGV) and the rest of large CNVs are common across donors. Overlap of SNVs with 1KG set (1000 genomes) is high (93.8%-96.8%) and the proportion of other SNVs is highly similar to the 1KG set. We therefore conclude that none of the study participants displayed gross genomic abnormalities. Supp Figure 1 and additional methods text have been added to the manuscript.*

1.8

It is a little disappointing that all 94,257 cells from the spleen were immune cells, apparently the result of no enzymatic digestion.

*We understand the reviewer’s point and agree that it is disappointing that the spleen data lacks non-immune cells. The original reason for avoiding enzymatic digestion was to dissociate the tissue as rapidly as possible, aiming to reduce artefacts introduced by prolonged enzyme treatment. That said, we find that even with a rapid non-enzymatic dissociation we still observe stress-related changes. Unfortunately all single cell dissociation protocols create some level of bias in the cell populations recovered and throughout this study we have used three different dissociation protocols for the different tissues (mechanical, collagenase and trypsin). Interestingly the stress signatures all show some similarity to each other.*

Reviewer 2

2.1

The authors claim that lung and esophagus were stable for 72h for all of the metrics they tested, and that some changes were observed in spleen at this time, but not before. They claim that this approach can be useful for future HCA study design. A restriction to their conclusions is that lung dissociation mostly released immune cells, but nearly no other cell types. For me, it is important to test additional protocols of dissociation in order to ensure a better dissociation of lung parenchyma, thus establishing on a more comprehensive number of cell types the benefits of cryopreservation.

*We agree with the reviewer that our lung dissociation protocol is immune-biased. We have tested a different single cell dissociation protocols (liberase / trypsin - based) and single nuclei RNA sequencing (sn-RNA-seq) on one additional lung donor (see figure below) and do observe different proportions of cell types recovered. It is now widely established in the single-cell RNA-sequencing field that different protocols and dissociation methods recover different cell types, and do not necessarily reflect the original proportions of cell types in tissues.*

*While we only have data for one donor, the single-nuclei RNA sequencing detects expectedly more Alveolar Type 2 and especially Alveolar Type 1 cells which are sensitive to any of the dissociation methods. Stromal and endothelial cell proportions are similar between nuclei and Collagenase D treatment, whilst immune cells are less represented. Notably in the nuclei preparation a number of cell types could not be detected. We see no clear monocyte population and T and NK cells could not be resolved (T-NK). Conversely, all cell types we detect with nuclear sequencing were also detected in the collagenase protocol that we used in this study. The liberase/trypsin protocols released far more stromal and fibroblast cells, but AT1/AT2 cells were also depleted and therefore offered no particular advantage over the protocol we used.*

*We therefore conclude that our study design should be able to capture any cell-type specific response to cold storage. While the proportion of AT1 cells is small, we detect these cells in every donor and time point (Supplementary table 2).*

*At this time, it is not feasible for us to perform sn-RNA-seq for the study. Furthermore, we think that sequencing single cells is of greater value for the question we asked. Single nuclei sequencing is usually done from frozen material, which sets no limits to sample storage as they can be frozen at the time of collection. Whilst this argues for the use of sn-RNA-seq, the nuclei data is known to be of worse quality with fewer genes detected (*[*https://journals.plos.org/plosone/article?id=10.1371/journal.pone.0209648*](https://journals.plos.org/plosone/article?id=10.1371/journal.pone.0209648) *and Figure c below) making it more difficult to detect cell types and calculate differentially expressed genes. Furthermore, cell type enrichment is difficult for snRNA-seq.*

*Overall, all the cell types were observed with our protocols, although in different proportions than in the original tissue. If there were an effect of storage time on specific cell types, we would expect it to be detected in our study design.*

*As the data below is only for n=1, we do not propose to include this in the manuscript, but would consider showing*

2.2

 It is also indicated that the annotated data will be available, but this link was not accessible when I tested it.

*The data is available for interactive viewing as well as an analysis file both for Python users as .h5ad and for R users as an .rds file in here:* [*https://www.tissuestabilitycellatlas.org/*](https://www.tissuestabilitycellatlas.org/)

2.3

Figure 4j and Supplementary Figure 10 suggest that time of storage has less impact on gene expression than cell types, number of counts or donor. Intriguingly, cell type explains no more than 10% of the variance, and overall, it seems that a large part of the variability is unexplained. This has to be clarified. In my opinion, each cell-type has probably its specific behavior. From the shown graphs, it seems that the total percentage of explained variance is probably below 20%. That implies that 80% of it comes from additional variables. A clarification of this point is key, because it will rate more clearly the impact of time of storage.

*The reviewer points out a valid concern regarding scRNA-seq data which has previously been reported to have high variability (*[*https://genome.cshlp.org/content/21/7/1160.long*](https://genome.cshlp.org/content/21/7/1160.long)*). The unexplained or residual variance in known to be very high for most genes most likely due to high dropout rate. A similar analysis was carried out for scRNA-seq data created with smart-seq2 protocol showing that only a few percent of variance were explained by different variables, and over 80% variance remained as residuals.*

2.4

To demonstrate the usefulness of cold conservation, additional results should be provided, regarding the morphology of the different cell types, the percentage of live cells, etc. This would complement well paragraphs about diversity of cell types, tissue processing, and annotation of cell types.

*We agree with the reviewer that the usefulness of this study is limited to the aspects that we have measured: sc-RNA-seq, bulk RNA-seq, cryosectioning morphology with H&E staining and manual cell viability counts. While addition of other protocols and cell type specific viability stains would have been interesting, we selected the most widely used methods in this study that were feasible to do in our processing pipeline at the time. However, we have added TUNEL staining on our frozen tissues as suggested by the reviewer 1.*

*Unfortunately, it will not be possible to collect any viability data retrospectively from the donated, processed tissues.*

2.5

The central hypothesis is that cold storage in an appropriate buffer will slow down RNA degradation. The observation of stable RIN numbers around 7 already provide an indication about the real stability of the samples, although it does not exclude cell-specific effects. These correct values probably explain the acceptable quality of the reads that is shown in Figure 1 and supplementary figure 1. Long term storage however resulted in spleen in an increased % of mitochondrial sequences, and of intronic reads, raising the interesting observation that nuclear mRNA is less susceptible to enzymatic degradation (a good point for using single cell NucSeq protocols).

*We agree with the reviewer that our data could indicate better resistance of nuclear transcriptome to storage time. While nuclear sequencing will be important for cell types that are sensitive to dissociation, the data will be of lower quality and not necessarily help to identify novel cell types even in brain (*[*https://journals.plos.org/plosone/article?id=10.1371/journal.pone.0209648*](https://journals.plos.org/plosone/article?id=10.1371/journal.pone.0209648)*). Although the storage time effect could be reduced via nuclear sequencing, we recommend processing of fresh cell samples within 24h when possible, which should avoid the increase in mitochondrial and intronic reads in a variety of cell types. Furthermore, nuclear sequencing can be performed on frozen tissues, in which case the cold storage time will not be relevant since the samples can be frozen at the site of collection, thus reducing cold storage time to a minimum.*

2.6

The authors refer to "ambient RNA" for droplets that contain 1-2 UMIs, "debris" for droplets that contain 3-100 UMIs and "cellular material" for droplets that contain more than 100 UMIs. They notice that the mean number of UMI increased in debris and decreased in cellular droplets by 72h (but not 24h) in spleen (Figure 3.c, d), but not in the two other tissues. While this is understandable, a direct comparison with a cell viability assay would be very valuable.

*We agree with the reviewer, that cell viability is an important QC measure and and the number of viable cells was recorded and is given in Supplementary Figure 7. A functional assay was not part of our original experimental plan, it would be too difficult to add at this stage.*

*However, to address the issue of cell death, we have carried out TUNEL apoptosis assays on tissue sections (Supplementary Figure 8, as a response to the reviewer 1) and added further discussion to the manuscript, following the description of the UMI results.*

*The TUNEL staining results are consistent with our interpretation that higher cell death in spleen is the most likely cause of increased cell debris.*

2.7 More details about the protocol of cold conservation should be provided regarding sampling areas (samples are 0.5 to 2 cm3), ratio between volumes of submersion and tissue, etc. I suspect that these parameters can be important and can affect the results. Hence, protocols should be more carefully documented. As a non-limitative list of issues : can there be an effect of the surface of exposition to the solution ? What are the evidences that the solution really penetrates into the core of each sample ? What are the differences between tissues?

*We apologise for providing insufficient detail. Sample sizes for different tissues are now described in more detail in the methods under ‘donor samples’. Regarding whether the solution penetrates to the core of each sample; the oesophagus mucosa samples are very thin (<3mm), lung parenchyma is very porous and spleen is very soft, so we would expect good penetration in these tissues. However, it is possible that the Hypothermasol solution may penetrate different tissues, particularly more solid organs, to different extents. We have added a note into the methods to ensure that future investigators using this method are aware of this.*

2.8

Formally, this clearly demonstrates that the same dissociation protocol will not be applicable for any cell type. For instance, any conclusion about non-immune lung cells have probably to be considered with some caution, as it is clear that this preservation method is not adapted to study lung epithelial cells.

*We agree with the reviewer (and acknowledge that this is widely accepted in the field) that the choice of the dissociation protocol will affect the cell type proportions gained. In the current study, we have tried three different methods: collagenase D treatment for lung, trypsin treatment for oesophagus and no enzymatic treatment for spleen. Collagenase treatment is admittedly biased towards immune cells (see figure below and Manuscript Figure 4) and acknowledge this in the manuscript methods and in the chapter entitled “Variation in cell type contribution” with a sentence “For example, the tissue dissociation protocol employed for lung strongly enriches for immune cell types.“. The trypsin treatment will recover epithelial cells better due to stronger effect on highly-connected epithelial cells, which is reflected in oesophagus data (Figure 4). The spleen is lacking non-immune cell types due to the lack of dissociation (Figure 4). As the reviewer correctly points out, the dissociation method clearly will have an impact on the cell types recovered. However, in lung we are capturing all the expected cell types with our collagenase treated scRNA-seq (see answers for 2.1), but admittedly capture less Alveolar Type 1 cells than for example with single nuclei sequencing.*

*Unfortunately, it was not feasible to carry out the study with multiple protocols on every tissue.*

2.9

After comparing bulk and Sc-bulk, the authors conclude that differences are due to the dissociation protocol rather than to conservation. Surprisingly, while dissociation in lung is supposed to extract mostly immune cells, and few epithelial cells, they report p-values and log2ratio that do not necessarily fit with this idea! For instance, FOXJ1 and TPPP3, 2 specific markers of ciliated cells, are not differentially expressed between bulk and pseudo-bulk! The same situation for MUC5B and MUC5AC, two markers of mucous cells, KRT5, a marker of basal cells. Surprisingly, SCGB1A1, a marker of CLUB cells is enriched in pseudo-bulk!

*Wilcoxon signed rank test p-value is 0.0003 for FOXJ1, 0.1 for TPPP3, 0.01 for MUC5B, 0.1 for MUC5AC and 0.08 for KRT5. All genes have higher expression in the bulk data indicated by positive median log2 fold change (Supplementary Table 4), suggesting loss of epithelial cells after digestion. However, only FOXJ1 is significant after Benjamini-Hochberg multiple testing for over 30,000 genes involved in analysis. This is most likely a power issue with very few replicas in both bulk and sc-pseudo-bulk groups and with large number of tests (genes) for the p-value correction. However, as the reviewer correctly points out, the club cell marker SCGB1A1 indicates higher expression in sc-pseudo-bulk, although this is not significant after multiple testing. While a single marker can appear significant only by chance, we checked other human club cell markers MSMB, BPIFB1, SCGB3A1 and BPIFA1 and noticed that they either have very high p-values or negative median log2 fold change indicating no differential expression between sc-pseudo-bulk and bulk. Furthermore, we saw that the expression of those genes is restricted to very few cells in sc-RNA-seq (UMAP plot below), which can also indicate that we originally had very few club cells in our lung parenchyma tissue sections. This is supported by previous findings where the club cells are mainly found in the airway part of the lung and not in parenchyma:*

[*https://www.nature.com/articles/s41591-019-0468-5*](https://www.nature.com/articles/s41591-019-0468-5)*,*

*Figure 1.E*

*This is consistent with the findings that the club cells markers are not detected as significantly changed between bulk and sc-pseudo-bulk potentially due to low expression in both samples, most likely due to very low cell numbers in the original tissue biopsy from parenchyma.*

2.10

In conclusion, this work contains interesting information regarding processing of human samples, but complementary approaches are needed to provide a definitive demonstration of its applicability.

*We have performed sc-RNA-seq, bulk RNA-seq, cryosectioning morphology with H&E staining and manual cell viability counts as the most widely used methods in this study that were feasible to do in our processing pipeline at the time. However, we have added TUNEL staining on our frozen tissues as suggested by the reviewer 1 and further performed analysis of different digestion methods in lung. Preliminary results indicated that alternative digestion methods were not more informative and were not included in the manuscript due to n=1.*

2.11

Citation of supp table 5 is wrong. This is supp table 4.

*Thank you for this comment, we have now revised the tables and figures to match the text.*

2.12

Supp table 5 is not quoted

*We have now quoted all supplementary figures and tables.*

2.13

Supplementary Figure 1 : scale bar is lacking for lung and spleen

*Thank you for bringing this to our attention. The scale bars in the lung and spleen images have now been made clearer.*

2.14

Legends of Figure 5 and Supplementary Figure 12: "Benjamini and Hochberg", not "Benjamin and Hochberg"

*Legends are now updated*

Reviewer 3

3.1

It seems the correlations with tissue necrosis (an individual donor factor which could have a major influence on expression variance) and cell death were established by trypan blue and/or histology of the organs, or by scRNA-seq alone. It would be of value to the dataset to either perform some IHC on the frozen sections/FFPE from the sampled organ regions to document the basic metrics commented on (eg viability, proportion of replicating cells, proportion of the major cell types determined by antibody markers) and the major cell type proportions present. This could also be done by FACS if there is remaining tissue available.

*Thank you for this useful suggestion. As you will see in our response to reviewer 1, point 4, we have now performed TUNEL (end stage apoptosis) staining on tissue sections from all 3 organs at 0h and 72h. This demonstrates low levels of cell death in the lung and oesophagus and higher levels in the spleen at T0. Due to the patchy nature of the staining and limited area of tissue available on sections, the staining is hard to quantitate. Nevertheless we see a clear trend of increased apoptosis with time. As the reviewer suggests, FACS would be a better solution for assessing proportions; however it is now impossbile to access fresh tissue sections (only preserved sections remain) to carry out this analysis. It is well known that different dissociation protocols create bias in cell type proportions; therefore while we capture the majority of cell types, we are aware that the actual proportions may not reflect the original tissue and we acknowledge this in the text.*

3.2

The conclusions/discussion could do a better job of synthesizing both the key findings and commenting on the limitations - the main one being that the number of individuals sampled and tissue types will potentially limit some of the conclusions - the authors do mention this along the way, but it would be easier for the reader to have the key synthesis in the discussion/conclusions. The need to evaluate/minimize/control for the consequences of ischaemia and dissociation method (less directly examined in this study) in each tissue type being studied by scRNA-seq is a key finding that should be highlighted in my view.

*Thank you for this suggestion. We have added text to the conclusion to highlight these findings.*

Reviewer 4

4.1

The studies includes many arguments that directly relate to the design of HCA projects. However, the work will be of interest for many other projects in the single-cell community. Thus, the conclusions could be formulated more general.

*Thank you for the suggestion. We have added text to the conclusion to make it clearer*

4.2

Since degradation starts from the 5' transcript ends, it would be of interest to see if profiles obtained using the 10x Genomics 5' assays would be affected. Many future (autoimmune) disease-focused projects will perform 5' profiling combined with TCR genotyping. Thus, a proof-of-concept is important in this context and can be done on a single sample and tissue type.

*We agree with the reviewer that this is a valid concern. Our 3’ sc-RNA-seq analysis fails to capture the changes of reads’ 5’ locations directly and thus demonstrate the potential 5’ degradation in time. However, even with our 3’ data we are able to detect changes in the genomic location of the reads in 72h in spleen (Figure 2 A-D) from exonic to intronic. We speculate that this could indicate degradation of mRNA in the cytoplasm, since the reads on degradation-resistant unspliced nuclear mRNA become more prominent. This change, however, is only significant in spleen after 24h and not yet observed in lung and oesophagus for 72h time point.*

*Whilst the reviewer suggests that a single sample would be sufficient, we feel that a larger n would be required to draw robust conclusions, which is not feasible within a reasonable time frame.*

4.3

The Fig. 2 should be re-ordered to have the summary first and subsequently the spleen-specific analyses.

*Thank you for the suggestion. We have re-ordered the Figure 2 and text accordingly.*

4.4

The definition of empty drops and debris is challenging and thresholds need to be adjusted per sample. As the distribution is sequencing depth dependent a fixed cutoff could result in cells assigned to debris in lowly sequenced datasets. Can this threshold be defined automatically per sample, e.g. through the automatic detection of the inflection points (with EmptyDrops; Lun et al., 2018).

*The reviewer has a valid point, and we agree that the distribution is dependent on sequencing depth. Although experimentally we aimed for the same depth, there will be variability in the results. We have now normalised the UMI counts across the manuscript, and prepared new Figure 3 and Supplementary Figure 7 to match with the new analysis. The results are similar, and the previously described changes in the spleen are still statistically significant.*

*Unfortunately we did not think the automatic detection can work with this data. The curves of UMI distribution within droplets are specific to runs and correspond to time especially in spleen. In many earlier timepoints, we notice that the curve containing “debris” is merged with “ambient RNA”, which will make automatic detection difficult for some time points.*

4.5

The mean number of UMIs in debris increased at 72h. What about the total UMI numbers in empty drops? More informative would be to show the number of reads in debris and empty drops as this directly relates to the "wasted" sequencing budget. Also, the figure does not fit the text (e.g. no Figure 3d).

*While the proportion of droplets containing 0 or 1 counts is really high (Figure below, a), we acknowledge that it would be helpful to know whether the increased sequencing cost is affected. We performed analysis to compare the proportion of reads in non-cellular droplets (ambient RNA and debris). Figure b below shows that on average between 10 and 30 percent of reads are “wasted” for non-cellular droplets. While there seems to be an increase with storage time in spleen, it is not statistically significant. We also tried to remove the ambient RNA from analysis and compare the proportion of reads in debris only, but this also yielded no significant results between spleen 0h and 72h samples (c). This could be due to low number of replicas. We do not report this non-significant result in the manuscript.*

*We have ensured that Figure 3 subpanels and corresponding text match, thank you for noticing this.*

a

*b*

c

4.6

The results of the viability analysis might be a good fit as introduction of the cell composition results. At the moment they seem to be a bit disconnected from the results, also because lung tissue is mentioned as affected.

*We had an internal discussion regarding the viability results and considered both locations: 1) the current location together with the discussions of increasing debris and ambient RNA contamination, and 2) as introduction of the cell composition results. We considered the benefits of both, and argue that the current location would allow for better possibilities to discuss the results in the context of cellular death and decided that this is a better fit for the flow of the manuscript. This is further supported by the comment from Reviewer 2 who expressed interest in further viability studies in the chapter with ambient RNA and debris.*

4.7

As the focus of the work is on the preservation test, the description of the tissue composition might be reduced. Important is the cell type proportion between time points and donors and not the detailed annotation.

*In order to detect every cell-type specific change in time, we needed to annotate the cell types as finely as possible. While often the cell type annotations take up a lot of space in the manuscript text due to explanation of marker genes, we have reduced this to a minimum while annotating altogether 72 populations from three tissues. We agree that it is not directly relevant to the current study to discuss the cell type composition between donors and adjacent tissue sections, however multiple of our collaborators pointed out the practical importance of this knowledge with regards to the glands in the oesophagus, immune infiltration or endothelial cells. Due to a lack of studies with multiple donors and comparison of adjacent tissue slices, we regarded this as valuable information for the community, and decided to include this in the discussion section of the manuscript. Cell type annotation is also available via the portal, increasing the usefulness of the data.*

*We have now performed statistical analysis on the changing cell type proportions in time, and included this in the manuscript, as well as new Supplementary Figures 12 and 13.*

4.8

The composition analysis and the cell type signatures should come directly after the presentation of tissue heterogeneity and annotation. Like this the work keeps the focus on the changes induced by timing. All extra analysis can be shifted to the end.

*Thank you for the suggestion. We originally tried this logic, however we decided to change it to the current structure in the course of writing and assembling figures. Namely, the analysis and visualisation in Figures 1-3 can be referred to as a QC, which was independent of cell type annotation and in practice is performed before the cell type and the signature analysis. We then proceeded to cell type annotation and analysis of cell-type specific effects with time.*

4.9

The mapping of cell types between tissues in certainly of interest, but seems to be a bit off-topic in this work. These valuable datasets will allow more specific analyses that go beyond this work and the authors better focus on the key messages of this study related to the conservation of gene expression profiles and tissue composition.

*We agree that the key messages of our manuscript are related to the changes with cold storage time. We conclude that the vast majority of cell types do not significantly decrease or change their transcriptome with cold storage time. This analysis and conclusion can only be drawn after careful and detailed cell type annotation. As pointed out earlier, we kept cell type descriptions as brief as possible, but believe that this is an integral and important part of our manuscript.*

*The small section analysing cell types across tissues allowed us to examine any effects of time with increased power arising from analysing similar cells from three tissues. Furthermore we wanted to anticipate the reader’s interest in the similarity of cell types isolated from different tissues. The tissue adaption signatures are of a wide interest, and as the reviewer pointed out, we hope that these datasets will allow the scientific community to perform further analysis.*

4.10

The conservation of cell type composition is a key message. Can the authors add more evidence (statistical tests) to support their conclusions that a) cell type composition is conserved over time points and b) the cell type variability is higher between donors than time points.

*Thank you for bringing our attention to this.*

*a) We have now looked further into the cell type composition changes in time and performed additional statistical tests. We found that that there were no significant changes in cell type proportion with time when performing a student’s t-test with Benjamini & Hochberg multiple testing correction (new Supplementary Table 5). There are trends of increasing B-cell proportions in spleen and decreasing T-cell proportions in lung and spleen (new Supplementary Figures 12 and 13). By increasing power through combining T0, 12h and 24h timepoints for comparison with 72h, the student’s t-test results were significant for two out of 72 populations: the fraction of Lung CD4 and CD8 T-cells decreased at 72h.*

*b) We performed one way ANOVA tests to compare effects between donors. The analysis indicated 29 out of 72 cell type-tissue combinations that have statistically significantly different proportion in at least one of the donors compared to the rest (BH corrected p-value < 0.05) (New Supplementary Table 5). The cell type most differentially retrieved between donors was Mast cells in Lung, for which the variability between donors is even visible on the main Figure 4.*

*We added Supplementary Figure 12a for demonstrating the variability between donors, and Supplementary Figure 12b for variability between timepoints.*

*We have added the previously mentioned supplementary table and figures to the revised manuscript, and added explanatory text to the manuscript.*

4.11

This is personal taste, but the details of the analyses (e.g. use of packages and software) can be moved to the Methods

*We agree with the reviewer and aimed to move all packages and software to the methods section.*

4.12

The title could be re-phrased as tissue stability can refer to many different parameters.

*We evaluated the title as it is, and although a longer title could improve accuracy, it would lose comprehensibility due to length. We think the current title is not misleading in its current form, specifying “cold preservation” and “scRNA-seq” with regards to the aspect of stability.*

4.13

It is mentioned twice in the beginning that the samples are also snap frozen for bulk RNAseq.

*Yes, the text indicates flash frozen bulk samples two times. In the first instance we refer to the lung samples for clinic time point (the earliest available), and for the other flash frozen samples we refer to all tissues and all time points. We have now added text to make this distinction clearer for the reader*

**Second round of review**

**Reviewer 2**

The authors have now substantially improved the quality of the manuscript, and they have clarified several issues. They explain well the interest of HypoThermasol. This solution has indeed already been tested by many laboratories, and having a correct demonstration of its usefulness would be appropriate. However, I also believe that the demonstration needs to be extremely strong to deserve a publication in Genome Biology.
At this point, I believe that there are still some last clarifications that are needed before making the article suitable for publication.
My first concern regards the title of the article: for me, the demonstration that Lung, spleen and oesophagus tissue remains stable for scRNA-seq in cold preservation is still incomplete, because many different cell types have not been described. In lung only, there are just three epithelial cell types that are identified (AT1, AT2 and ciliated cells). This is clearly insufficient to claim that the whole tissue is stable for scRNA-seq, and I am afraid that a definitive demonstration will not be possible. A situation in which the protocol would miss systematically some cell types would be extremely problematic for the Human Cell Atlas. My suggestion is to modify the title and become less affirmative, as in "scRNA-seq assessment of lung, spleen and oesophagus stability after cold preservation" or equivalent. In the evaluation of the the lung cells, it is said that there are only three epithelial cell types, which is insufficient. I feel that the epithelial group has been insufficiently characterized and indeed contains other cell types. This is for instance suggested by the detection of secretoglobin positive cells. An alternative would be to better characterize these cells.

Supplementary Figures 12b and Supplementary Figure 13 are very difficult to read. I suggest to replace them by tables, or to provide associated tables that can be easier to analyze.

The authors claim that in Figure 4, UMAP plots did not reveal an obvious effect of time on any tissue. I don't understand why the spleen is not behaving in a different manner by comparison with the other samples. This can be expected according to figures 1-4. The authors should clarify that point.

**Reviewer 4**

I have no further comments.

**Authors Response**

**Point-by-point responses to the reviewers’ comments:**

Comment

*Response*

**Reviewer 2**

2.1 My first concern regards the title of the article: for me, the demonstration that Lung, spleen and oesophagus tissue remains stable for scRNA-seq in cold preservation is still incomplete, because many different cell types have not been described. In lung only, there are just three epithelial cell types that are identified (AT1, AT2 and ciliated cells). This is clearly insufficient to claim that the whole tissue is stable for scRNA-seq, and I am afraid that a definitive demonstration will not be possible. A situation in which the protocol would miss systematically some cell types would be extremely problematic for the Human Cell Atlas. My suggestion is to modify the title and become less affirmative, as in "scRNA-seq assessment of lung, spleen and oesophagus stability after cold preservation" or equivalent. positive cells. An alternative would be to better characterize these cells.

*We have now changed the title to "scRNAseq assessment of human lung, spleen and oesophagus tissue stability after cold preservation" as suggested by the reviewer*

2.2 In the evaluation of the lung cells, it is said that there are only three epithelial cell types, which is insufficient. I feel that the epithelial group has been insufficiently characterized and indeed contains other cell types. This is for instance suggested by the detection of secretoglobin.

*We have now added Supplementary Figure 11 to show the expression of Club cell markers such as secretoglobins, and added a sentence about this to the cell type identification chapter “Lung club cell marker genes are detected in a small number of cells, but our clustering algorithm did not recognise them as a separate cluster (Supplementary Figure 11).”*

2.3 Supplementary Figures 12b and Supplementary Figure 13 are very difficult to read. I suggest to replace them by tables, or to provide associated tables that can be easier to analyze.

*We have added the cell type proportions from Supplementary Figures 12 and 13 (Supplementary Figures 13 and 14 with updated labels) to Supplementary Table 2 and updated the manuscript accordingly.*

2.4 The authors claim that in Figure 4, UMAP plots did not reveal an obvious effect of time on any tissue. I don't understand why the spleen is not behaving in a different manner by comparison with the other samples. This can be expected according to figures 1-4. The authors should clarify that point.

*UMAP plots show general differences between the cell populations, but are usually not strongly affected by a small number of genes, especially if these genes are not part of the "highly variable genes" selection that are used to generate the UMAP plot. In our variance analysis in Figure 4j, we find that only a few genes are deregulated as a result of cold storage. Furthermore, the effect of mitochondrial percentage was regressed out in the analysis in order to avoid cells clustering by the percentage of mitochondria rather than by the cell type. We have updated text as follows in order to avoid misunderstandings among the readers: “Notably, UMAP plots that were calculated on highly variable genes did not reveal an obvious effect of time (Figure 4g-h).”*
